# De novo development of small cyclic peptides that are orally bioavailable

**DOI:** 10.1038/s41589-023-01496-y

**Published:** 2023-12-28

**Authors:** Manuel L. Merz, Sevan Habeshian, Bo Li, Jean-Alexandre G. L. David, Alexander L. Nielsen, Xinjian Ji, Khaled Il Khwildy, Maury M. Duany Benitez, Phoukham Phothirath, Christian Heinis

**Affiliations:** 1 Institute of Chemical Sciences and Engineering, School of Basic Sciences, Ecole Polytechnique Fédérale de Lausanne (EPFL), Lausanne, Switzerland; 2 Center of Phenogenomics, School of Life Sciences, Ecole Polytechnique Fédérale de Lausanne (EPFL), Lausanne, Switzerland

**Keywords:** Combinatorial libraries, Drug delivery, Combinatorial libraries, Peptides, Screening

## Abstract

Cyclic peptides can bind challenging disease targets with high affinity and specificity, offering enormous opportunities for addressing unmet medical needs. However, as with biological drugs, most cyclic peptides cannot be applied orally because they are rapidly digested and/or display low absorption in the gastrointestinal tract, hampering their development as therapeutics. In this study, we developed a combinatorial synthesis and screening approach based on sequential cyclization and one-pot peptide acylation and screening, with the possibility of simultaneously interrogating activity and permeability. In a proof of concept, we synthesized a library of 8,448 cyclic peptides and screened them against the disease target thrombin. Our workflow allowed multiple iterative cycles of library synthesis and yielded cyclic peptides with nanomolar affinities, high stabilities and an oral bioavailability (%F) as high as 18% in rats. This method for generating orally available peptides is general and provides a promising push toward unlocking the full potential of peptides as therapeutics.

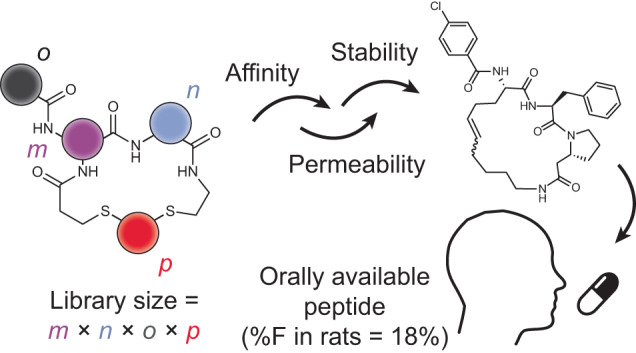

## Abstract (web summary)

Cyclic peptides show promise for modulating difficult disease targets; however, they often cannot be administered orally. The authors developed a method to synthesize and screen large libraries of small cyclic peptides while enabling the simultaneous interrogation of activity and permeability. This approach was applied to the disease target thrombin to discover peptides with high affinity, stability and oral bioavailability of up to 18% in rats.

## Main

One of the biggest bottlenecks in therapeutic development is that a large proportion of the proteins can currently not be targeted by classical small molecules administered orally^1^. Challenging targets include proteins with flat, featureless surfaces to which small molecules can hardly bind. Even targets such as enzymes, receptors or ion channels, which have pockets or clefts for ligand binding, can occasionally be challenging, for example, when selectivity for a specific protein homolog is required. The much larger biologics that can selectively target these interactions require injection^2^. A solution could potentially be offered by cyclic peptides due to their ability to bind challenging targets with high affinity while being relatively small and, thus, in reach of being orally available^3,4^. In fact, a small number of peptides derived from natural sources developed into oral drugs indicate the feasibility of orally available peptides, including cyclosporine A, desmopressin and a somatostatin analogue^5,6^. However, the vast majority of natural and de novo developed peptides have a large size and polar surface, or are proteolytically not stable, which forces administration by injection. If it was possible to develop target-selective peptides that are orally available, several unmet medical needs could be addressed^7,8^.

Toward the generation of oral peptides to new targets, an in-depth comparison of physicochemical parameters from orally available versus non-available examples pointed to the molecular weight (MW), the number of hydrogen bond donors (HBDs) and the polar surface area (PSA) as the most decisive factors^9,10^. Most oral peptides have a molecular weight below 700 Da, a polar surface smaller than 200 Å^2^ and five or fewer H-bond donors^5,11^. This is not a hard rule, though, as shown by the much larger cyclosporine A (CsA; 1,203 Da) that has many *N*-methylated amide bonds and can adapt a conformation to limit its polar surface. Studies with model peptides showed that *N*-methylation can be applied strategically to reach an oral availability as high as 30% for cyclic hexapeptides^12–14^. Most recently, computationally designed cyclic peptides with as many as 12 amino acids, with amide groups engaging in internal hydrogen bonding interactions for shielding the polar surface, were shown to permeate cellular membranes and be orally available in murine models^15^. However, as the strategically *N*-methylated model peptides, these peptides still need to be furnished with target-binding properties for drug development. Taken together, there are increasing data showing that cyclic peptides can be orally available, but the de novo generation of target-specific, orally available peptides still poses a formidable challenge.

For generating cyclic peptides that are target specific and have the required physicochemical boundaries, large libraries were screened by phage or mRNA display^16,17^. For instance, we recently applied phage display to generate cyclic peptides to withstand the proteolytic pressure of the gastrointestinal tract, but even our smallest peptides were still too large (9-mer; 1,134 Da), were too polar and had almost no oral bioavailability in mice (0.2%)^18^. Overall, biological display libraries appear to have an insufficient chemical and structural diversity to develop short peptides (for example, 6-mers) that have a high affinity, especially if the libraries are limited to mostly canonical amino acids. DNA-encoded libraries (DELs) allow a much wider choice of hundreds of unnatural amino acids and other chemical building blocks and were used for screening cyclic peptide and macrocycle libraries^19^. Although the synthesis of cyclic peptide DELs is challenging due to the large number of sequential coupling and deprotection steps in the presence of DNA, promising results have been achieved in recent years^20,21^. With the goal of generating chemically and structurally highly diverse libraries that can be screened by functional assays instead of affinity selections, we developed strategies in which cyclic peptides are combinatorially synthesized in microwell plates at a nanomole scale via acoustic dispensing and can be rapidly screened as crude products in the same plates using diverse types of assays^22–24^. For example, we generated 20,000 cyclic peptides by diversifying around 200 disulfide-cyclized peptides carrying an amino group with 100 carboxylic acids and identified nanomolar binders^24^. However, the labile disulfide bridge in these peptides rendered them unsuitable for oral application, but no other suitable cyclization chemistries were readily compatible with this rapid, one-pot synthesis and screening technology.

**Fig. 1:**
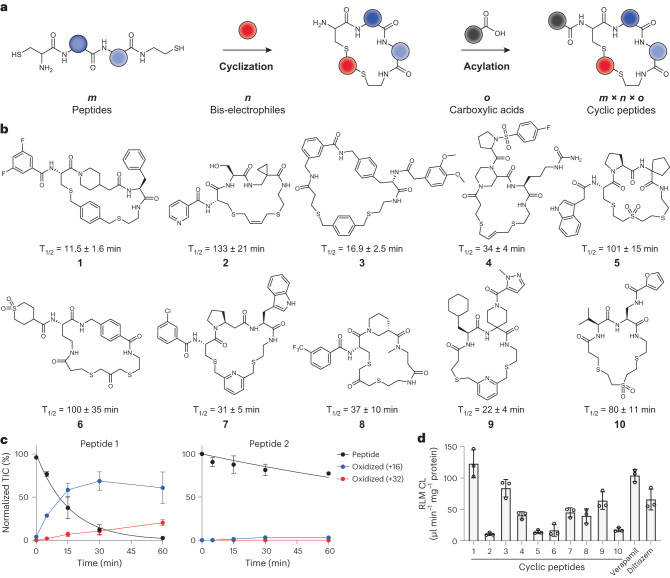
Synthesis and metabolic stability of dithioether cyclic peptides. **a**, General depiction of the library synthesis strategy. Linear dithiol peptides are cyclized with bis-electrophiles and are *N*-acylated with carboxylic acids to generate large cyclic peptide libraries. **b**, Chemical structures of random cyclic peptides **1–10** and half-lives in RLMs. Mean and s.d. values of three independent measurements are indicated. **c**, Comparison of metabolic stability in RLMs for a peptide that degrades rapidly (peptide **1**, left) versus slowly (peptide **2**, right). The concentration of intact peptide is shown by the black curve. The concentration of metabolized peptide is shown in blue for the addition of a single oxygen and in red for the addition of two oxygens. Mean and s.d. values of three independent measurements are indicated. **d**, CL for the 10 peptides and two oral drugs in RLMs. Mean and s.d. values of three independent measurements are shown. Source data

In this work, we expanded the above-described nanomole-scale peptide library synthesis to function with thioether-cyclized peptides that are more metabolically stable upon oral administration than the previously used disulfide-cyclized peptides. For synthesizing the libraries, we conceived a two-step combinatorial synthesis strategy in which ‘*m*’ random peptides are cyclized by ‘*n*’ linkers to obtain thioether-cyclized peptides, which are then acylated at a peripheral amine with ‘*o*’ carboxylic acids to yield *m* × *n* × *o* cyclic peptides (Fig. 1a). We first assessed if this peptide format is metabolically stable and then established conditions and procedures to combine the two combinatorial diversification steps. We used this approach to synthesize and screen a large library against the disease target thrombin^25^. Finally, we modified the library screening approach to include readouts for stability and membrane permeability, which we used to iteratively improve these qualities in our hit peptides. In the end, this work culminated in a five-building-block cyclic peptide that displayed an oral availability of 18% in rats. Overall, this work provides a set of synthesis and screening tools and a workflow that can be implemented for any target interaction that has a functional readout to move the field closer to the longstanding goal of developing target-specific peptides that can be administrated orally.

## Results

### Thioether-cyclized peptides can be metabolically stable

We envisioned developing cyclic peptides by reacting linear peptides flanked by thiol groups with a bis-electrophilic linker that would leave two thioether bonds in the final product (Fig. 1a). This cyclization is particularly efficient and clean, which allows for product screening without purification and thus access to large libraries, but thioether bonds are at risk of metabolic oxidation. We, thus, assessed the metabolic stability in detail by synthesizing 10 diverse dithioether cyclic peptides **1–10** that differed in the amino acid composition and contained variable cyclization linkers (Fig. 1b and Supplementary Fig. 1). Because the oxidation stems particularly from contact with cytochrome enzymes of the CYP450 family in the liver, a process called first-pass metabolism^26^, we incubated these peptides with liver microsomes and monitored metabolic stability and breakdown by mass spectrometry (MS). The 10 cyclic peptides varied strongly in their stability, with half-lives ranging from 11 min (peptide **1**) to 133 min (peptide **2**) (Fig. 1b), wherein no obvious correlation between oxidation rate and type of linker was found. For peptides with shorter half-lives, species with masses of M + 16 and M + 32 were detected, indicating oxidation at one or two sites (Fig. 1c and Supplementary Figs. 2 and 3). As a control and benchmark for the stability assay, we used two approved drugs, verapamil and diltiazem, that both have a rather low but sufficiently high stability with a metabolic clearance in humans of around 15 ml min^−1^ kg^−1^ (ref. ^27^). The side-by-side comparison showed that nine of the 10 cyclic peptides were similar or more stable than verapamil and diltiazem, wherein the four most stable ones were much more stable (>4-fold slower clearance (CL), CL in rat liver microsomes (RLMs) <20 μl min^−1^ mg^−1^; Fig. 1d). We, thus, expected that, for many peptides of this format, the thioether may be sufficiently stable for oral application.

### Efficient sequential peptide cyclization and acylation

**Fig. 2:**
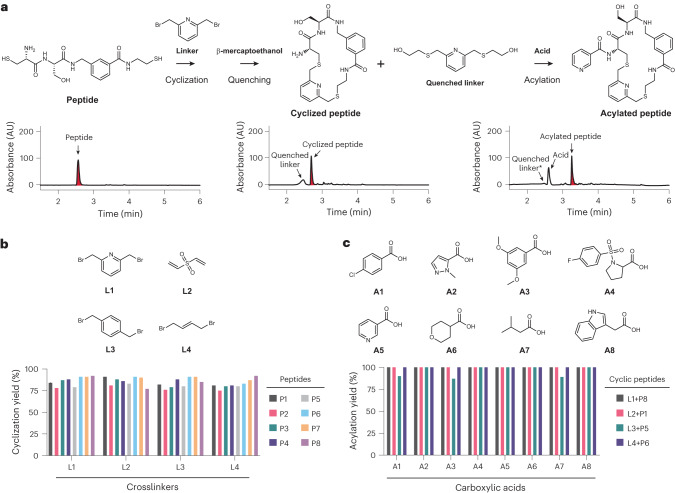
Cyclization and *N*-acylation of peptides for library generation. **a**, Cyclization and acylation reactions shown for an example peptide. The peptide was analyzed by HPLC before and after the reactions measuring absorbance at 220 nm. Peaks for the peptide and the desired products are shown in red. The asterisk marks the peak corresponding to the quenched linker that became smaller after the acylation reaction. **b**, Cyclization of peptides with bis-electrophilic linkers L1–L4. Cyclization yield determined by LC–MS is shown. **c**, Efficiency of *N*-acylation reactions. Cyclic peptides and carboxylic acids activated as NHS esters were transferred by acoustic dispensing. Reaction yield is indicated as percent of cyclic peptide acylated with carboxylic acid and was determined by LC–MS. Source data

**Extended Data Fig. 1:**
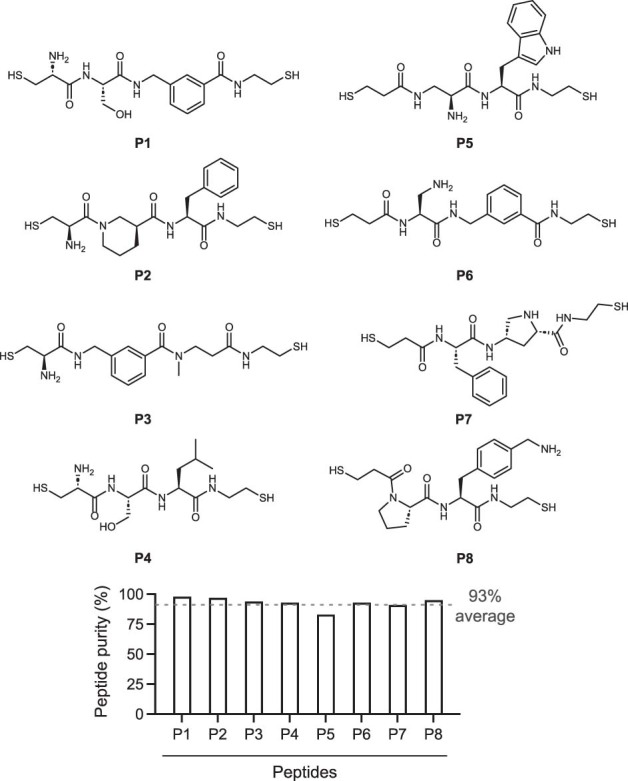
Synthesis of peptides for library generation. Structures of random dithiol peptides P1–P8 synthesized in 96-well plates and purified by precipitation in diethyl ether. The purity assessed by LC-MS is indicated. Source data

For the high-throughput synthesis of cyclic peptide libraries, we planned to avoid intermediate purification steps using sequential thiol-to-thiol peptide cyclizations followed by amine acylations (Fig. 1a) that required only the stepwise addition of reagents to microwell plates. The two reactions were both previously used for efficiently synthesizing and screening crude cyclic peptide libraries^24,28^ but not in combination. To assess the compatibility of the reactions and reagents, we applied them to eight random peptides (P1–P8) all containing thiol groups at both ends and an amino group, and we analyzed the intermediate and final products by liquid chromatography–mass spectrometry (LC–MS) (Fig. 2a). For efficiently accessing hundreds of short peptides containing two thiol groups, we established a synthesis on cysteamine resin in 96-well plates that yielded crude peptides at decent purity by ether precipitation. This was all completed without the need for a chromatographic purification step, as demonstrated by the average purity of 93% for the eight peptides (Extended Data Fig. 1 and Supplementary Fig. 4).

We next tested the efficiency of the cyclization reaction by combinatorically reacting the eight peptides, P1–P8, with four different bis-electrophilic linker reagents, L1–L4, in 96-well plates and analyzed yield and purity (Fig. 2b and Supplementary Fig. 5). To favor intramolecular reactions over intermolecular ones, the reactions were performed at low peptide concentrations (1 mM), in large volumes (1 ml) and using two equivalents of linker reagent over peptide. We observed high cyclization efficiencies across all 32 reactions, averaging 85% of the desired cyclic product. The most frequent side product was disulfide-cyclized peptide, which was found below a 1% yield for most reactions and reached to a maximum of only 15% (Supplementary Fig. 5). The high overall product yield for different sequences and linkers showed that the thiol-to-thiol reaction had a wide substrate tolerance despite the rather short length of the peptides. To prevent unreacted bis-electrophilic linker reagents from interfering with the subsequent acylation reaction, we quenched with β-mercaptoethanol and lyophilized the mixture to remove the excess quencher along with solvents and buffering agents. We obtained dry cyclic peptide containing only quenched cyclization linker as a major side product.

Next, we investigated the acylation efficiency of peripheral amino groups on the cyclized peptides following established procedures for picomole-scale synthesis^24^ and acoustic droplet ejection for reagent transfer^23^. We combinatorially reacted four different peptides that were all cyclized with a different linker at a 500-pmol scale with a four-fold molar excess of eight different carboxylic acids (A1–A8; 2,000 pmol) in 384-well plates. The 50-nl reactions were incubated at room temperature for 8 h, and excess activated acid was quenched by Tris-HCl buffer (Fig. 2c and Supplementary Fig. 6). We observed nearly quantitative *N*-acylation of all cyclic peptides, showing that the acylation reaction is compatible with the reagents of the preceding cyclization reaction and suggesting that the procedures are suitable for the synthesis of large and diverse cyclic peptide libraries.

### Potent thrombin inhibitors from an 8,448-member library

We applied the established synthesis procedure to the development of a selective inhibitor of thrombin, a trypsin-like serine protease that is an important target for controlling blood coagulation in thrombotic disorders^25^. The thrombin inhibitor dabigatran etexilate is an oral drug in clinical use, but its oral availability is relatively low at 6.5% (ref. ^29^). The active form of dabigatran contains two charged groups that are essential for target binding but need to be shielded for oral application in the form of a double pro-drug. In general, the development of non-charged, orally available thrombin inhibitors based on small molecules or cyclic peptides has been challenging^30^. In our previous efforts to generate cyclic peptide thrombin inhibitors, we were only able to identify inhibitors that were charged and, thus, not membrane permeable^22^ or that were disulfide cyclized and, thus, not redox stable for oral application^24^.

**Fig. 3:**
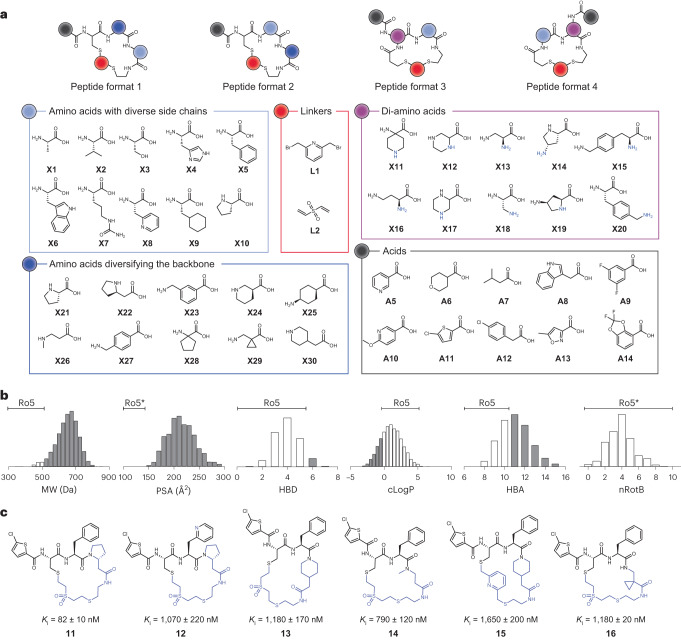
Synthesis of library of 8,448 cyclic peptides and screen against thrombin. **a**, Structures of amino acids, bis-electrophilic cyclization linkers and carboxylic acids used to synthesize the library. **b**, MW, PSA, number of HBDs, cLogP, number of HBAs and nRotBs of the cyclic peptides in the library. Peptides outside the rule-of-five (Ro5) space defined by Lipinski et al.^32^ are shown in gray. Asterisk indicates the physicochemical properties defined by Veber et al.^42^. **c**, Chemical structures and activities of purified cyclic peptides of group 1 indicated in Extended Data Fig. 2. The common core of the structure is colored in black. Mean and s.d. values of three independent measurements are shown. Source data

To overcome these limits, we designed a library of cyclic peptides that lack charged groups and that are cyclized by thioether bonds that cannot be linearized by reduction during transcellular transport (Fig. 3a). The peptides contain six building blocks, of which two are random amino acids (dark blue, light blue or violet); two are fixed as a thiol group, such as cysteine; one is a random cyclization linker (red); and one is a random peripheral carboxylic acid (black). For the acylation reaction, we used 10 structurally diverse carboxylic acids, including A11 that binds weakly to the S1 specificity pocket of thrombin^31^.

**Extended Data Fig. 2:**
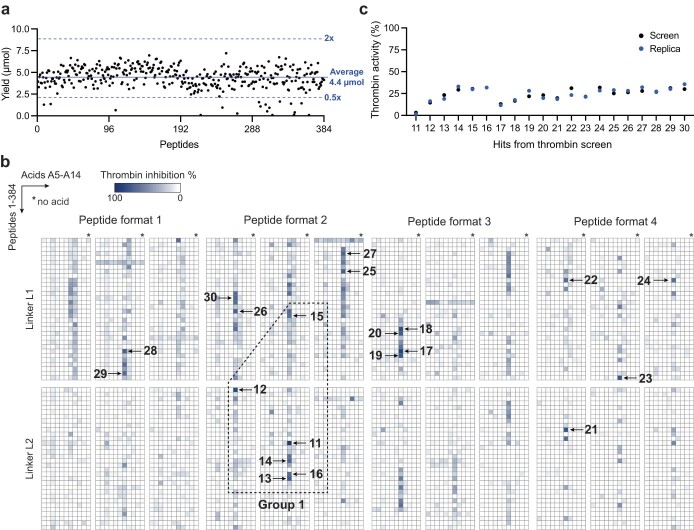
Screening 8,448 cyclic peptides against thrombin. **a**, Quantification of the 384 linear dithiol peptides using Ellman’s reagent. **b**, Heat map with residual thrombin activity measured for each cyclic peptide, divided into the two linkers used for cyclization and the four different generated peptide formats. Individual peptide sequences are aligned on the vertical axis, and the acids used for N-acylation are on the horizontal axis. The screen was carried out at a peptide concentration of 10 μM. Cyclic peptides forming a structurally related cluster and containing the most active hit are highlighted as Group 1. **c**, Thrombin inhibition of replicate reactions for the 20 best hits. Source data

We synthesized 384 short dithiol peptides of the total 400 possible sequences that could theoretically be formed based on the chosen four formats and the random amino acid building blocks (Fig. 3a). We produced the peptides by solid-phase peptide synthesis (SPPS) in 96-well plates and without purification and quantified the yield of all peptides using Ellman’s reagent, which showed a high average yield of 4.4 μmol (88% recovery based on resin loading) (Extended Data Fig. 2a). Using the established cyclization and acylation conditions described above, we cyclized the peptides at a 1-μmol scale and acylated the cyclized peptides at a 0.5-nmol scale in 384-well plates. This furnished us with a 8,448 cyclic peptides that were ready to screen in their plates with no further purification required. An analysis of the physico-chemical properties of the library showed that most of them exceeded the rule-of-five space that generalizes permeability rules^32^, showing that, despite their small size, the cyclic peptides still differed substantially from classical small-molecule drugs (Fig. 3b).

We screened the library against thrombin directly in the synthesis plates by dispensing the enzyme and fluorogenic substrate in aqueous solution to the reaction wells (Supplementary Table 1). This diluted the peptide to a final concentration of 10 μM and reduced the DMSO concentration to 1%, which was compatible with the target. We measured the residual thrombin activity and discovered that 73 out of 8,448 reactions (0.9%) inhibited thrombin by more than 50% (Extended Data Fig. 2b). Arranging the results in a heat map showed that all these most active peptides contained the carboxylic acid A11 or A12 (vertical lines in Extended Data Fig. 2b). Repeating the acylation reactions and thrombin inhibition screen for the 20 most active hits showed essentially the same activities and confirmed the robustness of the synthesis and screening procedures (Extended Data Fig. 2c). A structure–activity relationship analysis of the 20 top hits revealed six groups of peptides with related structures (peptides **11–30**; Fig. 3c and Supplementary Fig. 7). We re-synthesized and purified these cyclic peptides at larger scale to determine their *K*_i_ values, which were in the nanomolar range (Supplementary Fig. 8). The best candidate was cyclic peptide **11**, with a *K*_i_ of 82 ± 10 nM for human α-thrombin. Group 1 contained the most active peptides (cyclic peptides **11–16**), which shared the amino acid motif Cys-Phe (or Cys-Pal), and the amino group of cysteine was modified with chlorothiophene (Fig. 3c).

### Cyclic peptides display high proteolytic stability

**Fig. 4:**
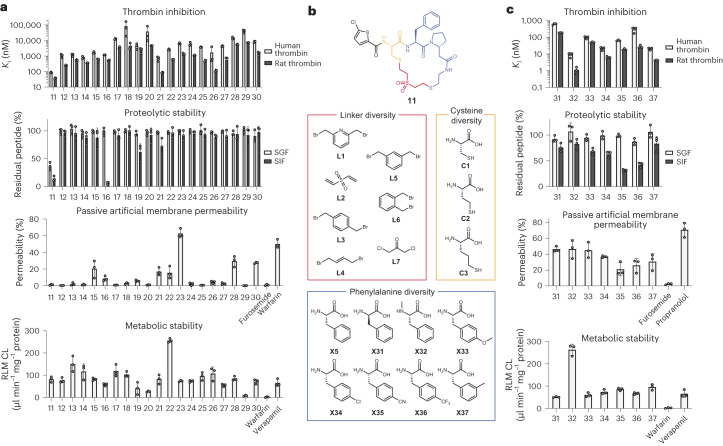
Thrombin inhibitor characterization and sub-library screen. **a**, In vitro characterization of peptides **11–30**. *K*_i_ and s.d. values of three independent measurements are shown. Proteolytic stability was measured by incubating peptide in SGF and SIF for 8 h at 37 °C. Mean and s.d. values of three independent measurements are shown. Passive membrane permeability was measured by PAMPA wherein cyclic peptides (50 μM) were added to the donor wells and incubated for 18 h. The permeability is indicated as the concentration in the acceptor well relative to the concentration in the wells at equilibrium (in %). Mean and s.d. values for three independent measurements are shown. logP_app_ is shown in Supplementary Table 2. Metabolic stability was measured by incubating the peptides with RLMs at 37 °C, and the CL was calculated based on the half-life for the disappearance of the peptide. Mean and s.d. values for three independent measurements are shown. **b**, Amino acids and cyclization linkers used for the synthesis of a sub-library based on cyclic peptide **11**. **c**, In vitro characterization of peptides **31–37** as in **a**. For all four parameters, mean and s.d. values for three independent measurements are shown. Permeabilities were measured by incubating the PAMPA plates for 12 h. Source data

We next assessed in vitro if the peptides display key properties for oral availability, such as proteolytic stability, membrane permeability and metabolic stability. As most peptide drugs are rapidly degraded by gastrointestinal proteases upon oral administration^33^, we first tested the proteolytic stability by exposing the peptides to either porcine pepsin at pH 1.2 (simulated gastric fluid (SGF)) or porcine pancreatin at pH 6.8 (simulated intestinal fluid (SIF)) for 8 h at 37 °C, which closely simulates the proteolytic pressure found in the human gastrointestinal tract. Most peptides had a high stability, which we attributed to their composition of mostly non-natural amino acids as well as their small size and, thus, limited conformational flexibility (Fig. 4a). We next assessed the membrane permeability in a parallel artificial membrane permeability assay (PAMPA)^34^ (Fig. 4a and Supplementary Table 2), which measures the diffusion of a compound from a donor to an acceptor well. The 20 cyclic peptides showed a large spread in passive membrane permeability, with some rapidly diffusing to the acceptor well and others being essentially impermeable (Fig. 4a; flux expressed in percent relative to concentration reached in the acceptor well at equilibrium). The data were converted into apparent permeability (logP_app_) values, which ranged from around −8 for completely impermeable compounds to −5 for fully permeable ones, with the best six peptides ranging from −6.0 ± 0.2 to −5.2 ± 0.1, which is close to well-permeable warfarin (logP_app_ = −5.3 ± 0.1), used as a reference in the same assay. We next assessed the metabolic stability via the rat liver microsomal assay, which tests the intrinsic clearance of compounds through the subcellular fractions of membrane-bound liver enzymes. In this assay, five peptides had a slower clearance and, thus, higher stability than verapamil, an oral drug that has a relatively fast hepatic clearance that we considered as the lowest acceptable stability for our cyclic peptides. Taking the in vitro characterization data together, we concluded that, in each of the in vitro assays, several of the peptides performed well, but no peptide had sufficient performance in all properties.

### Sub-library screen yields membrane permeable peptides

**Extended Data Fig. 3:**
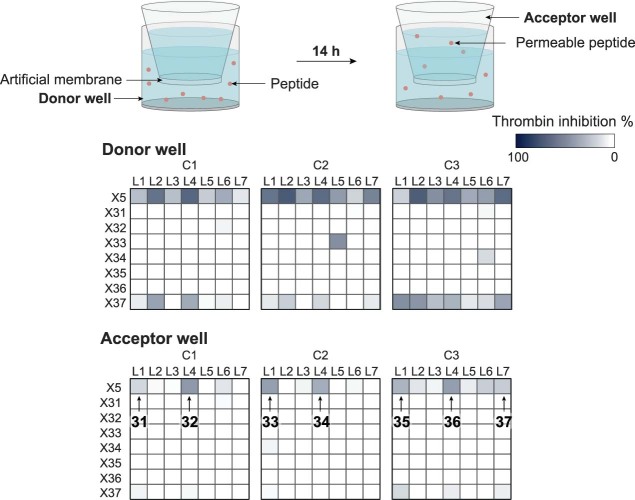
Thrombin inhibitor sub-library screen. Combined screening for permeability (PAMPA, 14 h, 5 μM) and thrombin inhibition. The heat maps show thrombin inhibition in donor (upper) and acceptor wells (lower). The screen was carried out at a theoretical equilibrium concentration of 1 μM. Source data

We sought to improve the permeability and metabolic stability starting from the most potent peptide **11**. Our efficient cyclic peptide synthesis procedure allowed us to build a new 168-member library with minimal effort. From the parent peptide **11**, we kept the β-homoproline and the chlorothiophene groups constant and combinatorially replaced the cysteine, phenylalanine and cyclization linker with analogous building blocks (Fig. 4b). The physico-chemical properties of the peptides in the sub-library again exceeded the rule-of-five space (Supplementary Fig. 9). To directly screen for inhibition and permeability, we next developed a procedure in which the crude reactions were applied to PAMPA wells, and the thrombin inhibition activities in the donor and acceptor wells were measured using the fluorogenic substrate (Extended Data Fig. 3). The inhibition data from the donor wells showed that replacing phenylalanine greatly reduced the activity but that changes in the cysteine side chain and cyclization linker were well tolerated. Data from the acceptor wells showed that peptides cyclized with linkers L1 and L4 were much more permeable than peptides with the parental sulfone linker L2. Peptides with linker L4 performed best in terms of activity and permeability.

We next prepared and purified cyclic peptides **31**–**37** and assessed their in vitro properties (Fig. 4c and Supplementary Fig. 10). The most potent cyclic peptide was **32**, which inhibited human and rat thrombin with *K*_i_ values of 8.9 ± 2.3 nM and 1.10 ± 0.41 nM, respectively, for a 10-fold improvement over its parent compound **11**. Most of the seven tested cyclic peptides had a high proteolytic stability and were able to withstand an 8-h incubation in the simulated gastrointestinal fluids (Fig. 4c). All peptides also showed a good passive diffusion, with logP_app_ values ranging from −5.6 ± 0.1 to −5.2 ± 0.1 (Supplementary Table 2). We assessed the metabolic stability of peptides **31**–**37** in RLMs, which cleared at a rate similar to the reference compound verapamil. We considered these peptides as sufficient except for the most potent peptide **32**, which was an outlier that cleared rapidly (CL = 260 ± 20 μl min^−1^ mg^−1^). We concluded that inhibition activity and passive membrane permeability were now sufficient, but the metabolic stability was too low for in vivo testing.

### Thioether bond replacement improves metabolic stability

**Fig. 5:**
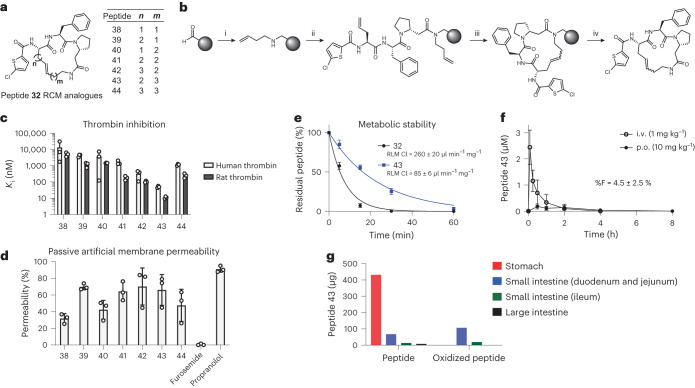
Peptides cyclized by hydrocarbon linkers (based on peptide 32). **a**, Structures of peptides **38**–**44** cyclized by RCM. The configuration of the alkene linker was not determined. **b**, Synthesis of peptides via RCM on solid phase. (i) Reductive amination: amine, NaBH_3_CN, DMF, AcOH 1%, 14 h at RT; (ii) SPPS: amino acid, HATU, DIPEA, DMF, 1–14 h at RT; (iii) RCM: 1st Generation Grubbs Catalyst (30 mol%), 1,2-dichloroethane, 2 h at RT under inert atmosphere; (iv) Cleavage: TFA:DCM:TIPS (50:49:1), 1 h at RT. **c**, Inhibition of α-thrombin. Mean and s.d. values of three independent measurements are shown. **d**, PAMPA of cyclic peptides (50 μM) incubated for 13 h. Mean and s.d. values for three independent measurements are shown. **e**, Comparison of the metabolic stability in RLMs of peptide **32** and RCM analogue **43**. Mean and s.d. values for three independent measurements are shown. **f**, Oral availability of peptide **43**. Plasma samples were analyzed by LC–MS after p.o. and i.v. administration to rats (*n* = 3; 10 mg kg^−1^). s.d. values are indicated. **g**, In vivo gastrointestinal stability of peptide **43**. Organs were harvested 30 min after p.o. administration to a rat (*n* = 1), and the quantity of peptide was analyzed by LC–MS. Oxidized peptide refers to a +16 mass adduct on the LC–MS. RT, room temperature. Source data

An LC–MS analysis of cyclic peptide **32** treated with RLMs showed two distinct +16 and +32 mass peaks that increased with longer incubation times, pointing to oxidation and possibly sulfoxide formation on the thioether bonds (Supplementary Fig. 11). We, thus, replaced both thioether bonds in **32** by cyclizing the peptide with all-hydrocarbon linkers (Fig. 5a), following a strategy based on ring-closing metathesis (RCM) on solid phase (Fig. 5b). Of the seven cyclic peptides **38**–**44**, which have structurally highly variable linkers, **43** was the most potent, inhibiting human and rat thrombin with *K*_i_ values of 50 ± 6 nM and 11.1 ± 1.4 nM, respectively (Fig. 5c and Supplementary Fig. 12). All seven cyclic peptides displayed high passive membrane permeability in the PAMPA (Fig. 5d and Supplementary Table 2). We next compared parent compound **32** with cyclic peptide **43**, which had the best thrombin inhibition, resistance to proteolytic degradation and a high permeability. In the microsomal assay, **43** had a threefold improved stability over **32** (RLM CL = 85 ± 6 μl min^−1^ mg^−1^; Fig. 5e and Supplementary Fig. 13).

We next tested the oral availability of **43** in rats (*n* = 3, application by gavage). Here, a blood sample analysis showed that the cyclic peptide reached the circulation, with a maximal plasma concentration (C_max_) of 183 ± 96 nM after 30 min and a corresponding oral bioavailability (%F) of 4.5 ± 2.5% (Fig. 5f). As a positive control and reference for the oral availability, we used the cyclic hexapeptide 1NMe3, designed by the Lokey laboratory to be membrane permeable and which showed an oral availability of 27 ± 4% (*n* = 3), corresponding to the reported range^12^ (Supplementary Fig. 14). To track peptide **43** and its potential degradation products, we again orally administrated **43** to rats (10 mg kg^−1^), killed them after 30 min and analyzed the peptide and potential metabolic products in the stomach, small intestine (duodenum and jejunum collected separately from the ileum) and large intestine. We found a large fraction of the peptide fully intact, although we also identified some oxidized peptide (+16 and +32 mass adducts) already in the gastrointestinal tract (Fig. 5g). We concluded that there is potential to improve the bioavailability if the metabolic stability could be further improved.

### Rapid diversification cycle improves oral availability

**Fig. 6:**
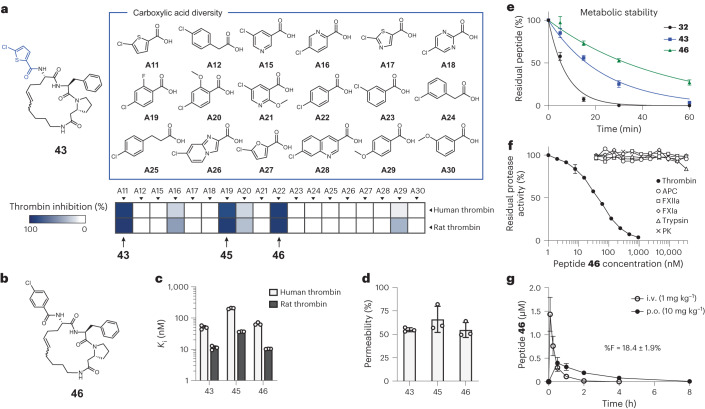
Cyclic peptides with improved metabolic stability and oral bioavailability. **a**, Sub-library based on **43** in which the carboxylic acid chlorothiophene (shown in blue) was substituted with the 17 analogues shown on the right using the acoustic dispensing library synthesis strategy. Heat map of human and rat thrombin inhibition of the sub-library is shown below. The screen was carried out at a peptide concentration of 10 μM. **b**, Chemical structure of peptide **46**. The molecule occurs in a 1:2 mixture of E:Z isomers, as determined by NMR (Supplementary Note 1). **c**, Inhibition of α-thrombin. Mean and s.d. values of three independent measurements are shown. **d**, Passive membrane permeability of the cyclic peptides (50 μM) was measured by PAMPA (12-h incubation). Mean and s.d. values of three independent measurements are shown. **e**, Metabolic stability of **46** in RLMs compared to the precursor compounds **32** (dithioether, chlorothiophene group) and **43** (RCM, chlorothiophene group). Mean and s.d. values for three independent measurements are shown. **f**, Specificity profiling of **46**. APC, activated protein C; PK, plasma kallikrein. Mean values of three independent measurements are shown. s.d. values are indicated for thrombin. **g**, Oral availability of **46** in rats (*n* = 3; 10 mg kg^−1^). s.d. values are indicated. Source data

Finally, we sought to eliminate the remaining oxidation site in **43**. MS attempts to identify the specific oxidation site in **43** were not successful due to failure in identifying an oxidized fragment, but we suspected that the chlorothiophene could be a vulnerable group (blue in Fig. 6a). We, thus, performed a rapid cycle of library synthesis and screening in which we varied the scaffold of **43** with structurally diverse carboxylic acids via the peripheral amino group (Fig. 6a). Given that **43** was likely binding in the S1 specificity pocket of thrombin, we chose 17 carboxylic acids that could also fit there, in addition to chlorothiophene that served as a positive control. We identified cyclic peptides **45** and **46** that inhibited thrombin with similar *K*_i_ values as the parent **43** (Fig. 6a). The slightly more potent **46** (Fig. 6b) contained 4-chlorobenzoic acid (A22) and inhibited human and rat thrombin with *K*_i_ values of 65 ± 5 nM and 10.3 ± 0.1 nM, respectively (Fig. 6c and Supplementary Fig. 15). Peptide **46** also displayed a high proteolytic stability and PAMPA permeability (Fig. 6d, Supplementary Fig. 16a and Supplementary Table 2) and was around twofold more stable in RLMs (Fig. 6e and Supplementary Fig. 16b). A specificity profiling and analysis of the inhibition mode showed that **46** binds selectively to thrombin and is a competitive inhibitor (Fig. 6f and Supplementary Fig. 17). We subsequently measured the oral bioavailability of peptide **46** in rats and found a C_max_ after oral administration of 400 ± 120 nM, which was twofold higher than peptide **43** (Fig. 6g). The oral bioavailability (%F) of peptide **46** increased as well to 18.4 ± 1.9%, approaching that of the designed reference peptide 1NMe3 that had an oral bioavailability (%F) of 27 ± 4%^12^.

## Discussion

Having first-hand experience of the excellent binding properties and therapeutic opportunities offered by peptides^16^ but, at the same time, being restricted by their parenteral administration, we established, in this study, a general method for de novo generation of small cyclic peptides that are orally available. A key step in this development was the choice of a dithioether cyclic peptide format that is stable enough to resist gastrointestinal proteases, small enough to enable gastrointestinal absorption and, notably, combined the thiol-to-thiol macrocyclization and the subsequent amine acylation. Together with acoustic dispensing technology, this peptide format allows for the efficient synthesis and screening of thousands of different peptides for identifying ligands to targets of interest as well as rapid iterative cycles of sub-library synthesis and screening. The unique format of our rapidly synthesized sub-libraries allowed us to screen simultaneously for two in vitro properties—membrane permeability and target binding—to more rapidly identify de novo peptide drug candidates. The peptide format based on a thioether cyclization was essential for efficiently accessing and screening large libraries: only the efficient and nearly quantitative cyclization enabled by thiol group/electrophile reaction allowed screening of crude products and, thus, large libraries.

We remain ambiguous whether the two thioether bonds in our cyclic peptides can be directly used as drugs or the thioether linkages need to be replaced at the end. On the one hand, thioether bonds are found on several orally available FDA-approved drugs^35^ and were also used in a recent example of model cyclic peptides (not binding to a target) that were orally available in rat or dog^36^. Additionally, several of our test peptides that showed only little oxidation upon exposure to liver cells had a substantially higher metabolic stability than approved oral drugs, such as verapamil or diltiazem. On the other hand, the stability of our most active cyclic peptides turned out to be limited by the thioether, which needed to be replaced. In the end, it could just be that much of the stability depends on the specific peptide sequence, and, at present, this will need to be evaluated on a case-by-case basis. An attractive future solution could be to cyclize the peptides from the beginning by RCM, as this would omit the linker replacement, which is cumbersome and bears the risk of losing binding affinity. Although much information is already available for RCM cyclization of peptides^37–40^, some development work is required to access large numbers of peptides that contain terminal alkene groups, to ensure efficient cyclization of diverse peptides and to assess if reagents used for RCM are compatible with the subsequent acylation reaction needed for peptide diversification.

Although most thrombin inhibitors contain a charged group for binding to the thrombin S1 specificity pocket, the cyclic peptides generated in this study are not charged, which is of great advantage for achieving oral availability. For example, the clinically approved thrombin inhibitor drug dabigatran contains two charges that were shielded by ethyl and carbamate esters to make an orally absorbed pro-drug (dabigatran etexilate). Not surprisingly, our thrombin inhibitor has a 4.5-fold higher oral availability in rats than dabigatran etexilate^41^. This offers substantial improvement over our previous attempts to generate thrombin inhibitors by screening combinatorial libraries of cyclic peptides, which produced inhibitors containing either charged groups that hindered oral application^22^ or a reducible disulfide bridge that limited stability^24^. So far, we have used only commercial building blocks to develop this thrombin inhibitor, but optimizations using custom-made building blocks will likely allow its further improvement to reach the sub-nanomolar potency required for pharmacological activity and therapeutic application.

The target-specific peptide developed de novo in this study has a decent oral availability. Our presented approach of synthesizing large numbers of small cyclic peptides with stable backbones is general and can be directly applied to other targets. A particularly attractive application may be the development of selective inhibitors or activators of enzymes, receptors or ion channels. Although these target classes are generally accessible to classical small molecules, achieving selectivity can occasionally be difficult but may be more easily achieved by cyclic peptides due to their constrained conformation. Of high interest are also challenging targets, such as protein–protein interactions, many of which are intractable to classical small molecules. Such targets likely need the screening of larger libraries, which is possible using our technique; the nanomolar scale, the combination of two diversification steps and the automated procedures should easily allow the synthesis and screening of hundreds of thousands or even millions of cyclic peptides. For challenging targets, it may be necessary to use larger cyclic peptides that can form more molecular contacts, although the peptides must not be too large so that membrane permeability and oral availability are still assured. We expect that the simplicity and robustness of the presented concepts and methods, combined with the proof that they can deliver orally available cyclic peptides, will encourage broad application and hopefully lead to the development of drugs addressing unmet medical needs.

## Methods

### General considerations

Reagents and solvents were purchased from commercial sources and used without purification, unless stated otherwise. Abbreviations: DMSO, dimethyl sulfoxide; HATU, *O*-(7-azabenzotriazol-1-yl)-*N*,*N*,*N*′,*N*′-tetramethyluronium hexafluorophosphate; DIPEA, *N*,*N*-diisopropylethylamine; NMM, 4-methylmorpholine; DMF, *N*,*N*′-dimethylformamide; DCM, dichloromethane; NMP, 1-methyl-2-pyrrolidinone; TFA, trifluoroacetic acid; EDT, 1,2-ethanedithiol; TIPS, triisopropylsilane; HSTU, *N*,*N*,*N*′,*N*′-tetramethyl-*O*-(*N*-succinimidyl)uronium hexafluorophosphate; HEPES, 4-(2-hydroxyethyl)-1-piperazine ethanesulfonic acid; BDT, 1,4-butanedithiol; TEA, triethylamine; and DCE, 1,2-dichloroethane.

### Preparative high-performance liquid chromatography

Peptides were purified by reversed-phase high-performance liquid chromatography (RP-HPLC) on either on a Waters HPLC system (Prep LC2535) using a Waters Sunfire Prep C18, 100 Å, 10 μm, 19 × 250 mm column at a flow rate of 16 ml min^−1^ or a Thermo Fisher Scientific UHPLC system (DIONEX UltiMate 3000) using a Waters Nova-Pak C18, 60 Å, 6 μm, 7.8 × 300-mm column at a flow rate of 4 ml min^−1^. Typically, a linear gradient of 10–60% or 30–80% solvent B in 35 min was applied. Solvent A: 99.9% (v/v) H_2_O + 0.1% (v/v) TFA; solvent B: 99.9% (v/v) acetonitrile + 0.1% (v/v) TFA.

### Analytical HPLC

Purified cyclic peptides were analyzed by RP-HPLC on an Agilent Technologies 1260 HPLC system using a C18 reversed-phase column (ZORBAX 300SB-C18, 300 Å, 5 μm, 250 × 4.6 mm, Agilent) and detection at 220 nm. A linear gradient of 0–100% (v/v) solvent B in 15 min was applied at a flow rate of 1 ml min^−1^. Solvent A: 94.9% (v/v) H_2_O + 5% (v/v) acetonitrile + 0.1% (v/v) TFA; solvent B: 99.9% (v/v) acetonitrile + 0.1% (v/v) TFA.

### Single quadrupole LC–MS

Peptide samples were analyzed by LC–MS using a Shimadzu LC–MS 2020 single quadrupole liquid chromatography mass spectrometer using a C18 reversed-phase column (Kinetex C18, 100 Å, 2.6 μm, 50 × 2.1 mm, Phenomenex). A linear gradient of 0–60% (v/v) solvent B or 10–100% (v/v) solvent B in 5 min was applied at a flow rate of 1 ml min^−1^ (solvent A: 99.95% (v/v) water + 0.05% (v/v) HCOOH; solvent B: 99.95% (v/v) acetonitrile + 0.05% (v/v) HCOOH). Masses were acquired by electrospray ionization mass spectrometry in positive ion mode. Data were analyzed by LabSolutions (version 5).

### High-resolution mass spectrometry

The masses of the important cyclic peptides were determined at high resolution (Supplementary Note 2). High-resolution mass spectrometry (HR-MS) analyses were performed on the Exploris 240 mass spectrometer (Thermo Fisher Scientific) interfaced with a chip-based nanoESI source (TriVersa Nanomate, Advion Biosciences), controlled by Chipsoft 8.3.1 software (Advion Biosciences). Samples were prepared in a solution of MeCN:H_2_O:HCOOH (50:49.9:0.1) at a final concentration of 100 nM. MS scans were acquired in the positive mode in the mass range 100–2000 *m*/*z* and at a resolution set to 120,000. Data analysis was carried out using XCalibur software version 4.1 (Thermo Fisher Scientific).

### On-resin synthesis of acylated peptides

Peptides were synthesized via a disulfide linker on solid phase. In brief, 263 mg of thiol-substituted resin (Polystyrene A SH resin, 200–400 mesh, 0.95 mmol g^−1^ loading, Rapp Polymere) was placed into a fritted 20-ml polypropylene syringe. The resin was washed twice with 10 ml of DCM and then swollen in 10 ml of MeOH/DCM 7:3 for 20 min. A quantity of 262.5 mg of 2-(2-pyridyldithio)ethylamine hydrochloride (1.1 mmol, 4.4 eq.) was dissolved in 2.9 ml of MeOH before addition of 6.6 ml of DCM and 50 μl of DIPEA. The resulting solution was added to the resin and let to react for 3 h at room temperature under agitation. The resin was then washed three times with 10 ml of MeOH/DCM 7:3 and three times with 10 ml of DMF to afford 250 μmol of cysteamine-PS resin.

The cysteamine-PS resin was suspended in DMF and split into 10 equal parts (26.3 mg, 25 μmol) in fritted 5-ml polypropylene syringes. Solid-phase peptide synthesis was carried out using standard Fmoc chemistry, and the individual coupling cycles were performed manually. Amino acids (0.1 mmol, 4 eq.) were activated with HATU (0.1 mmol, 4 eq.) and DIPEA (0.25 mmol, 10 eq.) in 400 μl of DMF and let to react with the resin for 1 h. The resin was washed six times with 4 ml of DMF, and the Fmoc protecting group was removed by incubating the resin twice with 4 ml of a 20% piperidine solution in DMF for 5 min. The resin was washed six times with 4 ml of DMF before proceeding to the next coupling cycle. For coupling of carboxylic acids onto a cysteine, Fmoc deprotection was followed by addition of the carboxylic acid (0.1 mmol, 4 eq.) activated with HATU (0.1 mmol, 4 eq.) and DIPEA (0.25 mmol, 10 eq.) in 400 μl of DMF for 1 h. For coupling of acids onto diamino acids, the Boc protecting group was removed on solid phase by adding TFA:TIS 19:1 for 1.5 h while the Fmoc protecting group was still attached to the second amine. Resin was washed three times with 4 ml of DCM and three times with 4 ml of DMF before proceeding to the coupling with activated carboxylic acids as described above. After completing the last coupling cycle, the resin was washed six times with 4 ml of DCM, and the resin was dried under vacuum for 14 h.

### On-resin deprotection and release of acylated peptides

Side chains were deprotected directly on the solid phase by adding a solution of TFA:TIPS:H_2_O (38:1:1) for 2 h. Resin was washed three times with 5 ml of DCM and twice with 5 ml of DMF. Peptides were cleaved from solid phase by reducing the disulfide bond with 1 ml of a solution of BDT (100 mM) and TEA (100 mM) in DMF, and the solution incubated with the resin for 14 h at room temperature. The resin was filtered off, and the solution containing the peptide was acidified by addition of 1.4 ml of a 1% TFA solution in DMF (2 eq. of acid relative to TEA). The solvent was then evaporated in a rotary vacuum concentrator (RVC 2–33 CDplus IR, Christ) for 14 h to afford the linear dithiol peptides as dry residues.

### Cyclization of acylated peptides

Dried linear dithiol peptides (25 μmol) were dissolved in 5 ml of acetonitrile mixed with 17.5 ml of a 60 mM NH_4_HCO_3_ buffer (pH 8.0). Peptides were cyclized by adding 2.5 ml of a 20 mM solution of the respective bis-electrophilic linker reagent (0.05 mmol, 2 eq.) in acetonitrile and incubation for 2 h at room temperature under gentle agitation. The excess reactive linker reagent was quenched by adding 1.25 ml of a 168 mM solution of 2-mercaptoethanol in Milli-Q water. After 1 h of quenching, the reaction products were analyzed by LC–MS. Cyclic peptides were dried in a rotary vacuum concentrator (RVC 2–33 CDplus IR, Christ) for 14 h before purification by preparative RP-HPLC.

### Calculation of physico-chemical properties of cyclic peptides

Structures of the acylated cyclic peptides were generated in ChemDraw 20.1 and SMILES strings exported as SD files. DataWarrior software (www.openmolecules.org, version 5.2.1) was used to calculate or estimate the following physico-chemical properties: MW, number of HBDs, number of HBAs, water/octanol partitioning coefficient (cLogP), PSA and number of rotatable bonds (nRotBs).

### Metabolic stability in RLMs

Metabolic stability was measured by incubating the peptides (10 μM) with RLMs. Peptides to test were dissolved in DMSO (4 mM), and 1 μl of the resulting solution was added to 365 μl of 0.1 M sodium phosphate buffer (pH 7.4). The solution was pre-warmed at 37 °C for 5 min before starting the reaction by addition of 24 μl of NADPH cofactor solution (22 mM) and 11 μl of RLMs (20 mg ml^−1^ protein concentration; Thermo Fisher Scientific, RTMCPL). The DMSO concentration in the microsomal incubation was 0.25% (v/v). Samples of 50 μl were removed at the following timepoints: 0 min, 5 min, 15 min, 30 min and 60 min, and microsomal activity was immediately stopped by addition of 150 μl of cold acetonitrile containing an internal standard peptide (1 μg ml^−1^). Samples were vortexed, sonicated for 5 min and centrifuged at 16,000*g* for 10 min. Supernatant was analyzed by LC–MS to quantify the disappearance of the compound and formation of degradation products. The half-life in RLMs was calculated using a one-phase decay function in GraphPad Prism 9 and converted to a clearance according to equation (1).

1 $${\rm{RLM\; CL}}=\frac{\mathrm{ln}(2)}{{{\rm{c}}\times {\rm{t}}}_{1/2}}$$

where c is the protein concentration in the incubation, and t_1/2_ is the half-life.

### Proteolytic cleavage and high-resolution tandem mass spectrometry analysis of peptides

Peptides were linearized by proteolytic cleavage to facilitate high-resolution tandem mass spectrometry (HR-MS/MS) and fragmentation analysis. For linearization with proteinase K (Promega, V302B), the enzyme was first reconstituted at 1 mg ml^−1^ in 100 mM Tris-HCl buffer (pH 8.0) supplemented with 10 mM CaCl_2_. For linearization with α-chymotrypsin (Sigma-Aldrich, 1023070001), the enzyme was reconstituted at a concentration of 2 mg ml^−1^ in 1 mM hydrochloric acid. Then, 2 μl of 4 mM cyclic peptide DMSO solution was diluted in 93 μl of 100 mM Tris-HCl (pH 8.0) containing 10 mM CaCl_2_, and 5 μl of the proteinase K or chymotrypsin solution was added at a final concentration of 50 μg ml^−1^ or 100 μg ml^−1^, respectively. The samples were incubated for 20 h at 30 °C or 37 °C for chymotrypsin and proteinase K, respectively, before quenching the protease activity by adding five volumes of methanol containing 0.1% (v/v) TFA and centrifuging the quenched protease samples at 14,000*g* for 10 min. Proteolytic cleavage efficiency was measured by injecting 10 μl of the resulting supernatant into the LC–MS and analyzing the area under the curve (AUC) ratio of cyclic to linear peptide in the total ion chromatogram (TIC).

HR-MS/MS of linearized peptides before and after treatment with liver microsomes was carried out using a Xevo G2-XS QTof (Waters); a hybrid, quadrupole, orthogonal acceleration, time-of-flight (TOF) MS system, which incorporates StepWave ion optics to achieve high levels of sensitivity; and QuanTof technology for high mass resolution. The mass analyzer is interfaced with ACQUITY UPLC pumps and sample manager systems. FastDDA mode was chosen with the following parameters: MS survey range 50–900 Da, five MS/MS ions per MS survey scan, accumulated TIC threshold 1 × 10^5^, injection time 250 ms and isolation window 3 Da.

For fragmentation analysis, 2 μl of samples was injected and applied on an ACQUITY UPLCBEH C18 reversed-phase column (1.7 μm, C18, 130 Å, 50 × 2.1 mm) (Waters) using a linear gradient of solvent B (0.05% (v/v) formic acid in acetonitrile) over solvent A (0.05% (v/v) formic acid in water) from 10% to 90% in 4 min at a flow rate of 0.5 ml min^−1^. Positive mode was used for mass data analysis. For fragmentation patterns of protonated target products, the isolated compounds were fragmentated in the collision cell. The collision gas was nitrogen, and the collision energy was controlled by collision energy ramp mode: LM CE Ramp range 6–9 V and HM CE Ramp range 20–60 V. Chemical identities were assigned to the fragment ions with a maximum mass error tolerance of 10 ppm. Mass Frontier Spectral Interpretation Software (Thermo Fisher Scientific, version 8) was used to predict fragmentation paths for the linearized peptides and cross-validated the proposed fragments.

### Peptide synthesis in 96-well plates

Peptides were synthesized on a MultiPep RSi parallel peptide synthesizer (Intavis) using standard Fmoc-based solid-phase synthesis on cysteamine-SASRIN resin (Bachem, D-2165, 0.4 mmol g^−1^ loading, 5-μmol scale). Coupling was carried out twice in a fritted 96-well solid-phase synthesis plate (Orochem, OF1100). Amino acids (26.5 μmol, 5.3 eq.) were activated with HATU (25 μmol, 5 eq.) and NMM (50 μmol, 10 eq.) in 114 μl of DMF and 5 μl of NMP and let to react with the resin for 45 min. Resin was washed once with 225 μl of DMF, and unreacted amines were capped with 5% acetic anhydride and 6% 2,6-lutidine in 100 μl of DMF for 5 min. The resin was washed six times with 225 μl of DMF, and Fmoc protecting groups were removed by reacting the resin twice with 120 μl of a 20% piperidine solution in DMF for 5 min. The resin was washed six times with 225 μl of DMF before the next coupling cycle. After completing the last coupling cycle, the resin was washed six times with 200 μl of DCM.

Peptides were cleaved from solid support by incubating the resin twice in 200 μl of reducing cleavage solution (TFA:H_2_O:EDT:TIPS, 94:2,5:2.5:1) for 1 h at room temperature. The resin was removed by filtration, and the combined flow-through was collected in a Nunc 96-well DeepWell polypropylene plate (Thermo Fisher Scientific, 278752). The cleavage solution volume was reduced to approximately 100 μl using a stream of N_2_ and subsequently cooled down on ice. Peptides were precipitated by adding approximately 1.5 ml of ice-cold diethylether and incubating at −20 °C overnight. Peptides were collected by centrifugation at 3,500*g* for 15 min at 4 °C. The pellet was washed by resuspension in 1.5 ml of ice-cold diethylether and collected again by centrifugation at 3,500*g* for 15 min at 4 °C. Peptides were dissolved in 1 ml of a 50% (v/v) acetonitrile solution in water.

### Peptide quantification with Ellman’s reagent

For quantification, 2 μl of the peptide solution in 50% (v/v) acetonitrile in water was mixed with 198 μl of a 0.2 mM solution of Ellman’s reagent 5,5′-dithio-bis-(2-nitrobenzoic acid) in 0.1 M sodium phosphate buffer (pH 8) and 1 mM EDTA in a clear 96-well flat-bottom plate (Greiner, 655101) and incubated at room temperature for 15 min. Absorbance was measured at 412 nm on a Tecan Infinite M200 Pro microplate reader. Peptide concentrations were determined using a dithiothreitol (DTT) calibration curve prepared according to the instructions above. Volumes corresponding to 1 μmol of peptide were transferred to a new Nunc 96-well DeepWell polypropylene plate and lyophilized.

### Peptide cyclization in 96-well plates

Peptides (1 μmol, 1 eq.) were dissolved in 700 μl of 60 mM NH_4_HCO_3_ buffer (pH 8.0) mixed with 200 μl of acetonitrile. A volume of 100 μl of a 20 mM solution of the respective linker reagent (2 μmol, 2 eq.) in acetonitrile was added to the peptide solution and incubated for 2 h at room temperature while shaking. Excess linker reagent was quenched by addition of 50 μl of a 168 mM solution of 2-mercaptoethanol (8 μmol, 8 eq.) and incubation for 1 h at room temperature. Cyclic peptides were analyzed by LC–MS and lyophilized.

### Acylation of cyclic peptides with acoustic droplet ejection

In contrast to previous protocols in which we used *N*,*N*,*N*′,*N*′*-*tetramethyl-*O*-(1*H*-benzotriazol-1-yl)uronium hexafluorophosphate (HBTU) to activate the carboxylic acid for the acylation of disulfide-cyclized peptides, we instead activated the acids using *N*,*N*,*N*′,*N*′-tetramethyl-*O*-(*N*-succinimidyl)uronium hexafluorophosphate (HSTU), which yielded *N*-hydroxysuccinimide (NHS) esters that were less prone to hydrolysis for a more robust protocol.

Acylation of cyclic peptides with carboxylic acids was carried out on the Echo 650 acoustic liquid handler (Labcyte). Cyclic peptides (1 μmol, 1 eq.) were dissolved in 50 μl of a DMSO solution containing 100 mM HEPES sodium salt. A volume of 10 μl of the resulting solution was transferred to an Echo Qualified 384-well Low Dead Volume microplate (Labcyte). Carboxylic acid (80 mM, 4 eq.), HSTU (80 mM, 4 eq.) and HEPES sodium salt (100 mM, 5 eq.) were dissolved in DMSO, and 10 μl of the resulting activated carboxylic acid solution was transferred to an Echo Qualified 384-well Low Dead Volume microplate (Labcyte).

Acoustic droplet ejection technology on the Echo 650 liquid handler was used to transfer 25 nl of both the cyclic peptide and the activated carboxylic acid solutions in a combinatorial fashion to a Nunc 384 shallow-well plate (Thermo Fisher Scientific, 264705). Plates were centrifuged at 1,800*g* for 1 min and incubated for 8 h at room temperature. Excess activated carboxylic acids were quenched for 14 h by adding 0.45 μl of DMSO and 4.5 μl of a 100 mM Tris-HCl buffer (pH 7.4) using a CERTUS FLEX liquid dispenser (Gyger).

The reaction progress was analyzed by quantifying unreacted cyclic peptide and acylated products on LC–MS. Approximately 0.5 nmol of the cyclic peptide was injected into the system, and a linear gradient of 10–100% (v/v) solvent B in 5 min was applied at a flow rate of 1 ml min^−1^ (solvent A: 99.95% (v/v) H_2_O + 0.05% (v/v) HCOOH; solvent B: 99.95% (v/v) acetonitrile + 0.05% (v/v) HCOOH).

### Thrombin library synthesis and screening

The peptides of the library have four different formats: two in which the carboxylic acids are linked to an N-terminal cysteine (formats 1 and 2) and two in which they are linked to a diamino acid (formats 3 and 4). For the random positions, we chose 10 α-amino acids with diverse side chains (light blue), 10 amino acids with diverse backbones (dark blue) and 10 structurally diverse diamino acids (violet). For the cyclization, we used linker reagents L1 and L2.

A quantity of 4.8 g of cysteamine-SASRIN resin (Bachem, D-2165, 0.4 mmol g^−1^ loading) was suspended in DMF to reach a volume of 40 ml. A volume of 100 μl of this suspension (5 μmol) was transferred to each well of four fritted 96-well plates (Orochem, OF1100), and the 384 peptides were synthesized in parallel as described above. After precipitation with diethyl ether and quantification with Ellman’s reagent, 1 μmol of each peptide was transferred in two replicates to new Nunc 96-well DeepWell polypropylene plates and lyophilized. Peptides that were obtained in quantities smaller than 1 μmol were not transferred but, instead, replaced with another peptide of the same library. To avoid duplicating cyclic peptides, a different linker was used for cyclization.

Lyophilized peptides were dissolved in 800 μl of 60 mM NH_4_HCO_3_ buffer (pH 8.0) and 100 μl of acetonitrile. Peptides were cyclized by addition of 100 μl of a 20 mM solution of the respective bis-electrophilic cyclization reagent (2 μmol, 2 eq.) in acetonitrile and incubation for 2 h at room temperature. Excess cyclization linker was quenched by addition of 50 μl of a 168 mM solution of 2-mercaptoethanol (8 μmol, 8 eq.) and incubation for 1 h at room temperature. Cyclic peptides were lyophilized and dissolved in 50 μl of DMSO containing 160 mM HEPES sodium salt to prepare 20 mM stock solutions. Carboxylic acids (4 eq.), HSTU (4 eq.) and HEPES sodium salt (5 eq.) were dissolved in DMSO to prepare 80 mM stock solutions of activated carboxylic acids. Activated carboxylic acid stocks and cyclic peptide stocks were transferred to Echo Qualified 384-well Low Dead Volume microplates (Labcyte).

The cyclic peptides were *N*-acylated with activated carboxylic acids as follows. Volumes of 25 nl of cyclic peptides and 25 nl of activated carboxylic acids were transferred in a combinatorial fashion to 384-well microplates (Greiner, 781076) using an Echo 650 acoustic liquid handler (Labcyte). Plates were centrifuged at 2,000*g* for 2 min and incubated at room temperature for 8 h. Excess activated carboxylic acids were quenched by sequential addition of 0.28 μl of DMSO and 16.33 μl of assay buffer containing 50 mM Tris-HCl (pH 7.4), 150 mM NaCl, 10 mM MgCl_2_, 1 mM CaCl_2_, 0.01% (v/v) Triton X-100 and 0.1% (w/v) BSA using a CERTUS FLEX liquid dispenser (Gyger) and incubation at room temperature for 14 h.

The reaction products were screened for thrombin inhibition as follows. Human α-thrombin (16.67 μl, 6 nM, Molecular Innovations) in assay buffer (50 mM Tris-HCl (pH 7.4), 150 mM NaCl, 10 mM MgCl_2_, 1 mM CaCl_2_, 0.01% (v/v) Triton X-100, 0.1% (w/v) BSA) was dispensed into each well using a CERTUS FLEX liquid dispenser (Gyger). Plates were incubated at room temperature for 10 min, and 16.67 μl of a 150 μM fluorogenic thrombin substrate solution (Z-Gly-Gly-Arg-AMC, Bachem) in assay buffer (50 mM Tris-HCl (pH 7.4), 150 mM NaCl, 10 mM MgCl_2_, 1 mM CaCl_2_, 0.01% (v/v) Triton X-100, 0.1% (w/v) BSA) and 1% DMSO were added to each well. Thrombin inhibition was determined by measuring the fluorescent gain over time. Fluorescence intensity was measured on a Tecan Infinite M200 Pro microplate reader for 30 min at 25 °C with a fluorescent readout every 3 min (excitation: 360 nm, emission: 465 nm). Protease inhibition was calculated by comparing the enzymatic activity to a control without cyclic peptide.

### Calculation of physical and chemical properties of thrombin library

Structures of the carboxylic acids and the cyclized peptides with unmodified amines were generated in ChemDraw 20.1 and exported as two separate SD files. The acylated cyclic peptides were directly generated in DataWarrior software (https://openmolecules.org/, version 5.2.1) using the Enumerate Combinatorial Library tool. The formation of an amide bond was defined to take place between carboxylic acid groups in the SD file for the carboxylic acid library and nitrogen atoms in the SD file for the cyclic peptide library. The nitrogen was thereby defined as ‘being substituted with at least 1 hydrogen’ and ‘not being part of an aromatic ring’. The carbon next to the nitrogen was defined as ‘not being aromatic’ and ‘not containing π electrons’. Additionally, a nitrogen engaged in an amide bond was entirely excluded from the reaction.

Using the prediction tools provided in DataWarrior, the physical and chemical properties MW, number of HBDs, number of HBAs, cLogP, PSA and nRotBs were calculated directly from the Enumerated Combinatorial Library.

### Preparative synthesis and purification of cyclic peptides

Peptides were synthesized on a MultiPep RSi parallel peptide synthesizer (Intavis) by standard Fmoc-based solid-phase peptide synthesis on cysteamine-SASRIN resin (Bachem, D-2165, 0.4 mmol g^−1^ loading, 50-μmol scale) or cysteamine 2-chlorotrityl resin (Sigma-Aldrich, 8.56000, 1.63 mmol g^−1^ loading, 50-μmol scale). Amino acids (0.21 mmol, 4.2 eq.) were activated with HATU (0.2 mmol, 3.9 eq.) and NMM (0.5 mmol, 10 eq.) in 870 μl of DMF and 5 μl of NMP and let to react with the resin for 45 min. Couplings were performed twice for each amino acid. Resin was washed once with 2 ml of DMF, and unreacted amines were capped with 5% acetic anhydride and 6% 2,6-lutidine in 800 μl of DMF for 5 min. The resin was washed six times with 2,000 μl of DMF, and the Fmoc protecting group was removed by incubating the resin twice with 800 μl of a 20% piperidine solution in DMF for 5 min. The resin was washed six times with 2,000 μl of DMF before proceeding to the next coupling cycle. After completing the last coupling cycle, the resin was washed six times with 2 ml of DCM.

Peptides were cleaved from the resin by adding 5 ml of a reducing cleavage solution (TFA:H_2_O:EDT:TIPS, 94:2.5:2.5:1) and incubating for 2 h at room temperature. The resin was removed by filtration, and the cleavage solution volume was reduced to approximately 1 ml under a stream of N_2_. Peptides were precipitated by adding approximately 45 ml of ice-cold diethylether and incubating at −20 °C overnight. Peptides were collected by centrifugation at 3,500*g* for 15 min at 4 °C, and residual ether was evaporated under a stream of N_2_. Pellets were dissolved in 40 ml of 60 mM NH_4_HCO_3_ buffer (pH 8) and 5 ml of acetonitrile.

Peptides were cyclized by adding 5 ml of the respective linker solution (20 mM, 2 eq.) and incubating at room temperature for 2 h. Excess crosslinker was quenched by addition of 500 μl of a 168 mM solution of 2-mercaptoethanol (8 eq.) and incubation for 1 h at room temperature. Cyclic peptides were lyophilized and purified by RP-HPLC. Fractions containing the cyclic peptide were lyophilized.

*N*-acylation of the cyclic peptides was performed as follows. Carboxylic acids (80 μmol, 4 eq.) were activated with HSTU (80 μmol, 4 eq.) and HEPES sodium salt (200 μmol, 10 eq.) in 1 ml of DMSO and added to the cyclic peptide (20 μmol, 1 eq.). The reaction was incubated for 8 h at room temperature, and the product was purified by preparative RP-HPLC as described above.

### Protease inhibition of cyclic peptides

The inhibition constants (*K*_i_) of cyclic peptides were calculated by measuring thrombin inhibition in the presence of different concentrations of the cyclic peptides. Two-fold dilution series within a concentration range from 66.67 μM to 31.79 pM in assay buffer (50 mM Tris-HCl (pH 7.4), 150 mM NaCl, 10 mM MgCl_2_, 1 mM CaCl_2_, 0.01% (v/v) Triton X-100, 0.1% (w/v) BSA) were prepared, and thrombin inhibition was measured as described above. Human α-thrombin (Molecular Innovations) and rat α-thrombin (Molecular Innovations) were used at final concentrations of 2 nM and 3 nM, respectively, and fluorogenic substrate (Z-Gly-Gly-Arg-AMC, Bachem) was used at a concentration of 50 μM. Enzymatic activity was determined by measuring fluorescent gain on a Tecan Infinite M200 Pro microplate reader (excitation: 360 nm, emission: 465 nm) or a PHERAstar FSX microplate reader (excitation: 380 nm, emission: 440 nm) for 30 min at 25 °C with a fluorescent readout every 3 min. The two instruments were controlled with Tecan Infinite M200 Pro instrument software (iControl, version 1.12) and PHERAstar FSX software (version 5.70, edition 8), respectively. Protease inhibition was calculated by comparing the enzymatic activity to a control without cyclic peptide.

Half maximal inhibitory concentration (IC_50_) values were determined using a four-parameter logistic regression model in GraphPad Prism 9 to fit sigmoidal curves to the acquired data points according to equation (2) below.

2 $${{y}}={\rm{b}}+\frac{{\rm{a}}-{\rm{b}}}{1+{10}^{(\log {{\rm{IC}}}_{50}-{{x}}){\rm{p}}}}$$

where a and b represent the measured inhibition maximum and minimum, respectively; *x* is the logarithm of peptide concentration; *y* is the thrombin activity compared to a control without the cyclic peptide; and p is the Hill coefficient.

*K*_i_ values were calculated using the IC_50_ and the Cheng–Prusoff equation (equation (3)).

3 $${K}_{{\rm{i}}}=\frac{{{{IC}}}_{50}}{1+\frac{{\left[{\rm{S}}\right]}_{0}}{\rm{K}_{{\rm{m}}}}}$$

where K_m_ is the Michaelis–Menten constant of the enzyme for the substrate used in the assay, and [S]_0_ is the initial substrate concentration. The K_m_ of human α-thrombin and rat α-thrombin for the substrate Z-Gly-Gly-Arg-AMC was measured to be 168 μM and 62 μM, respectively.

### Passive artificial membrane permeability

Membrane permeability of cyclic peptides was determined using a pre-coated PAMPA plate system (Corning BioCoat, 353015). The plate system consisted of a 96-well microwell plate (donor wells) and a 96-well insert containing a PVDF membrane coated with tri-layers of phospholipids (acceptor wells). Permeability was measured from the bottom plate (donor wells) to the top insert (acceptor wells). Cyclic peptide or reference compound solutions were prepared at a concentration of 50 μM in PBS buffer (pH 7.4) containing 5% DMSO, and 300 μl of the resulting solutions was placed into the donor wells of the PAMPA plate. Acceptor wells were prepared by adding 200 μl of PBS buffer (pH 7.4) containing 5% DMSO. The insert was stacked onto the donor plate, and the system was covered with a lid to avoid evaporation. After incubating the plates for 12–18 h at room temperature, the acceptor solutions were analyzed by LC–MS to determine permeable cyclic peptide concentrations. Additionally, 3:2 dilutions of the 50 μM cyclic peptide stocks were prepared in PBS buffer (pH 7.4) containing 5% DMSO to yield a sample corresponding to the theoretical equilibrium between donor and acceptor wells. The samples were run on the LC–MS to measure the cyclic peptide concentration representing maximum permeability. Permeability percent was calculated using equation (4) below.

4 $${\rm{Permeability}} \% =\frac{\left[{{\rm{Peptide}}}_{{\rm{Acceptor}}}\right]}{\left[{{\rm{Peptide}}}_{{\rm{Equilibrated}}}\right]}\times 100$$

where [Peptide_Acceptor_] represents the AUC measured on the LC–MS for the acceptor wells, and [Peptide_Equilibrated_] represents the AUC measured for the samples diluted to the theoretical equilibrium. Detection limit for the assay was typically at 1% permeability.

P_app_ values were calculated using equation (5).

5 $${{\rm{P}}}_{{\rm{app}}}=\frac{{{\rm{V}}}_{{\rm{D}}}\times {{\rm{V}}}_{{\rm{A}}}}{\left({{\rm{V}}}_{{\rm{D}}}+{{\rm{V}}}_{{\rm{A}}}\right)\times {\rm{A}}\times {\rm{t}}}\times -\mathrm{ln}\left(1-\frac{\left[{{\rm{Peptide}}}_{{\rm{Acceptor}}}\right]}{\left[{{\rm{Peptide}}}_{{\rm{Equilibrated}}}\right]}\right)$$

where V_D_ and V_A_ are the volumes of donor and acceptor wells, respectively; A is the area of the membrane; and t is the incubation time.

### Peptide stability in simulated gastric and simulated intestinal fluid

Simulated gastric and intestinal fluids were prepared according to the States Pharmacopeia specifications (Test Solutions, United States Pharmacopeia 35, NF 30, 2012). SGF: 0.2 g of sodium chloride was dissolved in 50 ml of water, and the pH was adjusted to 1.2 by addition of 0.7 ml of 10 M hydrochloric acid. Then, 0.32 g of porcine pepsin (Sigma-Aldrich, P7125) was dissolved under gentle agitation, and the volume was filled up to 100 ml with Milli-Q water. SIF: 0.68 g of monobasic potassium phosphate was dissolved in 25 ml of Milli-Q water, and the pH was adjusted to 6.8 by adding 7.7 ml of a 0.2 M sodium hydroxide solution. Next, 1 g of porcine pancreatin (Sigma-Aldrich, P3292) was added under gentle agitation, and the volume was adjusted to 100 ml with Milli-Q water to afford a fine suspension. To test the stability in simulated gastrointestinal fluids, 396 μl of SGF or SIF was pre-heated at 37 °C for 10 min, and 4 μl of a 1 mM peptide solution in DMSO was added. Then, 50 μl of samples was removed upon adding the peptide (T_0_) and after 8-h incubation at 37 °C (T_1_), and proteolytic activity was immediately stopped by addition of 150 μl of cold acetonitrile containing an internal standard (1 μg ml^−1^). Samples were vortexed, sonicated for 5 min and centrifuged at 16,000*g* for 10 min. Supernatant was analyzed by LC–MS to measure the peptide concentrations at the different timepoints. Residual peptide was calculated by normalizing the concentrations measured at T_1_ to T_0_.

### Sub-library synthesis

Linear dithiol peptides were synthesized in fritted 5-ml polypropylene syringes on a MultiPep RSi parallel peptide synthesizer (Intavis) by standard Fmoc-based solid-phase peptide synthesis on cysteamine 2-chlorotrityl resin (Sigma-Aldrich, 8.56000, 1.63 mmol g^−1^ loading, 25 μmol scale) as described above with small changes. Amino acids (65 μmol, 2.6 eq.) were activated with HATU (63 μmol, 2.5 eq.) and NMM (145 μmol, 5.8 eq.) in 280 μl of DMF and 5 μl of NMP and let to react with the resin (25 μmol, 1 eq.) for 45 min. Couplings were performed twice for each amino acid. Resin was washed once with 800 μl of DMF, and unreacted amines were capped with 5% acetic anhydride and 6% 2,6-lutidine in 400 μl of DMF for 5 min. The resin was washed six times with 600 μl of DMF, and Fmoc protecting groups were removed by incubating the resin twice with 450 μl of a 20% piperidine solution in DMF for 5 min. The resin was washed six times with 600 μl of DMF before proceeding to the next coupling cycle. After completing the last coupling cycle, the resin was washed six times with 600 ml of DCM. Peptide cleavage and ether precipitation were carried out as described above. After quantification with Ellman’s reagent, 1 μmol of each peptide was transferred in seven replicates to new Nunc 96-well DeepWell polypropylene plates and lyophilized. Peptides were cyclized as described above to afford 20 mM cyclic peptide stock solutions in DMSO containing 160 mM HEPES sodium salt. The amine on cyclic peptides was acylated with 5-chlorothiophene-2-carboxylic acid as follows. A 160 mM solution in 5-chlorothiophene-2-carboxylic acid was prepared in DMSO containing 160 mM HEPES sodium salt, and the acid was activated by mixing in equal volumes with a 160 mM HSTU solution in DMSO. Then, 10 μl of the activated carboxylic acid solutions and the cyclic peptide stocks were placed in Echo Qualified 384-well Low Dead Volume microplates (Labcyte), and 400 nl of cyclic peptide and 400 nl of HSTU-activated 5-chlorothiophene-2-carboxylic acid were transferred combinatorially to 384-well microplates (Greiner, 781076) using the Echo 650 acoustic liquid handler (Labcyte). Plates were centrifuged at 2,000 *g* for 2 min, and the reaction was allowed to proceed at room temperature for 8 h before addition of 39.2 μl of DMSO with a CERTUS FLEX liquid dispenser (Gyger). A volume of 25 μl of the resulting DMSO solutions was diluted with PBS buffer at pH 7.4 containing 5% DMSO in Nunc 96-well DeepWell polypropylene plates to afford 5 μM solutions of the acylated peptides.

### Sub-library screen for active and permeable cyclic peptides

Volumes of 300 μl of the acylated peptides were pipetted into the donor wells of pre-coated PAMPA plates (Corning BioCoat, 353015); 200 μl of PBS buffer at pH 7.4 containing 5% DMSO was added to the acceptor wells; and the insert was stacked on top of the donor plate to allow an exchange between donor and acceptor wells through the artificial lipid membrane. The PAMPA plates were covered with a lid to avoid evaporation and incubated for 14 h at room temperature. Additionally, a diluted donor sample was prepared at the concentration representing theoretical maximum permeability (3 μM). Thereby, 30 μl of the donor sample before incubation was diluted with 20 μl of PBS buffer at pH 7.4 containing 5% DMSO. Volumes of 16 μl of each acceptor well and the diluted donor samples were transferred to a 384-well microplate (Greiner, 781076), and thrombin activity was measured as follows. Human α-thrombin (16 μl, 6 nM, Molecular Innovations) in assay buffer was dispensed into each well using a CERTUS FLEX liquid dispenser (Gyger), and plates were incubated at room temperature for 10 min. Then, 16 μl of a 150 μM fluorogenic thrombin substrate solution (Z-Gly-Gly-Arg-AMC, Bachem) in assay buffer containing 1% DMSO was added to each well, and thrombin inhibition was determined by measuring the fluorescent gain over time. Fluorescence intensity was measured on a Tecan Infinite M200 Pro microplate reader for 30 min at 25 °C with a fluorescent readout every 3 min (excitation: 360 nm, emission: 465 nm). Protease inhibition was calculated by comparing the enzymatic activity to a control without cyclic peptide.

### On-resin synthesis of cyclic peptides via RCM

Peptides were synthesized on solid phase using a modified backbone amide linker (BAL) approach. A 4-formyl-3-methoxyphenyloxymethyl polystyrene resin (Bachem, D-2570, 50 μmol) was placed in 5 ml of fritted polypropylene syringes and washed twice with 4 ml of DMF. Reductive amination was carried out by dissolving the amine (0.5 mmol, 10 eq.) and sodium cyanoborohydride (0.5 mmol, 10 eq.) in 1 ml of DMF containing 1% of acetic acid and reacting the resulting suspension with the resin for 14 h at room temperature. The resin was washed six times with 4 ml of DMF, six times with 4 ml of MeOH and six times with 4 ml of DCM. Fmoc-d-β-homoproline (0.1 mmol, 2 eq.) was activated with HATU (0.1 mmol, 2 eq.) and DIPEA (0.25 mmol, 5 eq.) in 500 ml of DMF and let to react with the resin for 4 h at room temperature. Resin was washed once with 4 ml of DMF, and coupling was repeated for 14 h at room temperature. After completing the coupling, the resin was washed once with 4 ml of DMF, and unreacted amines were capped by addition of 4 ml of a 5% acetic anhydride and 6% 2,6-lutidine solution in DMF for 20 min. Remaining couplings and Fmoc deprotection were done as described above. Modification of the N-terminus with 5-chlorothiophene-2-carboxylic acid on solid phase was done by activating the acid (0.2 mmol, 4 eq.) with HATU (0.2 mmol, 4 eq.) and DIPEA (0.5 mmol, 10 eq.) in 500 ml of DMF and letting it react with the resin for 1 h. After completing the last coupling step, resin was washed six times with 4 ml of DMF and three times with 4 ml of DCM and dried under vacuum. The RCM was carried out as follows. Syringes were capped with a rubber septum, and nitrogen was flushed through via a needle for 10 min to create an inert atmosphere. Then, 30 mol% 1st Generation Grubbs Catalyst (Sigma-Aldrich, 579726) was dissolved in 1.5 ml of degassed DCE and added to the resin. The reaction was allowed to proceed for 2 h while maintaining a low nitrogen flow bubbling through the solution. Resin was then washed twice with 4 ml of DCE, and the reaction was repeated. Completion of the RCM was verified by microcleavage and LC–MS analysis. Thereby, a small amount of resin was placed in approximately 50 μl of TFA and incubated for 1 h. TFA was then evaporated with a nitrogen flow, and the residue was dissolved in 100 μl of a 50% (v/v) solution of acetonitrile in water. Resin was removed by filtration with a 0.22-μm syringe filter, and peptides were analyzed by LC–MS. After completing the RCM, the resin was washed six times with 4 ml of DCE, six times with 4 ml of MeOH, six times with 4 ml of DMF and six times with 4 ml of DCM. Peptides were cleaved from solid phase by incubating the resin with a solution of TFA:DCM:TIPS (50:49:1) for 1 h at room temperature. Resin was filtered off, and solvent was evaporated under nitrogen flow to yield a brown oil. Cyclized peptides were purified by preparative RP-HPLC as described above.

### Animal experiments

All animal experiments were carried out according to the terms of the Swiss animal protection law and were ethically reviewed and approved by the cantonal veterinary services (Canton of Vaud, Switzerland; license no. VD3606 and national license no. 32669).

### Measurement of oral availability in rats

Male Wistar Han rats (250–350 g) obtained at an age of 9–10 weeks from Charles River Laboratories were used for the studies and kept in housing groups of two or three. Experiments were performed at the age of 10–11 weeks. Intravenous (i.v.) administrations were carried out using tail vein injections. Peptides were dissolved in pure DMSO and diluted with 5% Cremophor EL in water (5% DMSO final) at a concentration of 0.5 mg ml^−1^. I.v. administration was carried out at a dose of 1 mg kg^−1^ body weight via the left lateral tail vein of rats (*n* = 3) using an Insyte-W 24 G × 0.75 in. (0.7 mm × 19 mm) temporary peripheral venous catheter (BD Biosciences, 381312). Before injecting the peptide solution, a correct placement of the catheter was confirmed by an apparent reflux of blood through the catheter. Orally (p.o.) administrated peptides were dissolved in DMSO and diluted with 10% Cremophor EL in water (5% DMSO final) at a concentration of 1 mg ml^−1^ and administrated by oral gavage to rats (*n* = 3) at a dose of 10 mg kg^−1^. Rats used for oral administration were fasted overnight for 14 h and provided access to food again 2 h after administration, whereas access to water was not restricted throughout the whole experimental procedure. Volumes of 100 μl of blood samples were collected via the right lateral tail vein at different timepoints after i.v. or p.o. administration into heparinized tubes (Sarstedt, 20.1282); the samples were stored on ice for up to 4 h; and plasma was obtained by centrifugation at 2,000*g* for 5 min at 4 °C. A volume of 40 μl of plasma was added to 120 μl of an acetonitrile:water (9:1) solution containing an internal standard (5 μg ml^−1^). Samples were vortexed for 10 s and sonicated for 10 min, and precipitated plasma proteins were pelleted by centrifugation at 16,000*g* for 15 min at 4 °C. Volumes of 120 μl of supernatant were transferred to a new tube, and solvent was evaporated in a rotary vacuum concentrator (RVC 2–33 CDplus IR, Christ) for 2 h at 40 °C. Dried residues were dissolved in 20 μl of a water:acetonitrile (7:3) (v/v) solution, centrifuged at 14,000*g* for 5 min and analyzed by LC–MS. Peptides were quantified by extracting ion chromatograms corresponding to their respective mass, integrating the area under the peptide peak and calculating the concentrations by normalization to a calibration curve. Calibration curves were prepared by analyzing spiked samples of blood or plasma with known concentrations of peptide. The absolute oral bioavailability was calculated using equation (6).

6 $${\rm{F}}=\frac{{{\rm{AUC}}}_{{\rm{p}}.{\rm{o}}.}}{{{\rm{AUC}}}_{{\rm{i}}.{\rm{v}}.}}\times \frac{{{\rm{Dose}}}_{{\rm{i}}.{\rm{v}}.}}{{{\rm{Dose}}}_{{\rm{p}}.{\rm{o}}.}}\times 100$$

where AUC_p.o._ and AUC_i.v._ are calculated from a plasma concentration versus time curve using the linear trapezoidal rule, and Dose_i.v._ and Dose_p.o._ are the intravenously or orally administrated amount of peptide in mg kg^−1^.

### Measurement of in vivo gastrointestinal stability in rats

Peptide **43** was dissolved in 10% Cremophor EL and 5% DMSO in water at a concentration of 1 mg ml^−1^ and administrated by oral gavage to a rat at a dose of 10 mg kg^−1^. The rat was killed 30 min after the administration using a CO_2_ chamber followed by cervical dislocation. Stomach, small intestine (duodenum/jejunum and ileum were collected separately) and large intestine were removed and immediately frozen in pre-cooled containers on dry ice. Organs and their respective content were collected separately to facilitate homogenization and sample processing. The organs were homogenized using a mortar and pestle on dry ice and liquid nitrogen to avoid thawing of the samples. Peptides were extracted by addition of 2 ml of extraction buffer containing 50 mM Tris-HCl (pH 8.0), 150 mM NaCl, 5 mM EDTA, 0.01% Triton-X, 1 mM PMSF and protease inhibitor cocktail (Sigma-Aldrich, P8340) per gram of organ or organ content weight and sonicating the resulting suspension for 5 min. Samples were centrifuged at 16,000*g* for 10 min, and 40 μl of the resulting supernatant was mixed with 120 μl of a cold acetonitrile:water (9:1) solution containing an internal standard (5 μg ml^−1^). Samples were analyzed and quantified by LC–MS as described above.

### Diversification and screening of RCM cyclic peptides

Cyclic peptide **43** without modification of the N-terminal amine was synthesized on solid phase at a 50-μmol scale using the modified BAL approach described above. The N-terminal amine was Fmoc deprotected after carrying out the RCM by addition of 4 ml of 20% piperidine in DMF. The resin was washed six times with 4 ml of DMF and six times with 4 ml of DCM before cleaving the peptides from solid phase by incubating the resin with a solution of TFA:DCM:TIPS (50:49:1) for 1 h at room temperature. Resin was removed by filtration, and the peptide was purified by RP-HPLC. A 20 mM stock solution of the pure peptide in DMSO containing 100 mM HEPES sodium salt was prepared and reacted in a combinatorial fashion with activated carboxylic acids in 384-well microplates (Greiner, 781076) using the Echo 650 acoustic liquid handler (Labcyte) as described above.

The reaction products were screened at a concentration of 10 μM for thrombin inhibition as follows. Human α-thrombin (16.67 μl, 6 nM, Molecular Innovations) or rat α-thrombin (16.67 μl, 9 nM, Innovative Research) in assay buffer (50 mM Tris-HCl (pH 7.4), 150 mM NaCl, 10 mM MgCl_2_, 1 mM CaCl_2_, 0.01% (v/v) Triton X-100 and 0.1% (w/v) BSA) was dispensed into each well using a CERTUS FLEX liquid dispenser (Gyger). Plates were incubated at room temperature for 10 min, and 16.67 μl of a 150 μM fluorogenic thrombin substrate solution (Z-Gly-Gly-Arg-AMC, Bachem) in assay buffer (50 mM Tris-HCl (pH 7.4), 150 mM NaCl, 10 mM MgCl_2_, 1 mM CaCl_2_, 0.01% (v/v) Triton X-100 and 0.1% (w/v) BSA) and 1% DMSO were added to each well. Thrombin inhibition was determined on a Tecan Infinite M200 Pro microplate reader as described above.

### Nuclear magnetic resonance analysis

Nuclear magnetic resonance (NMR) spectra were recorded on a Bruker Avance II (^1^H NMR recorded at 800 MHz) equipped with a proton-optimized triple-resonance NMR ‘inverse’ TCI cryoprobe and a Bruker Avance III-HD (^13^C NMR recorded at 151 MHz) equipped with a broadband optimized cryoprobe. The peptide **46** (2.2 mg) was prepared in 0.6 ml of CDCl_3_. Spectra were recorded at a temperature of 298 K. Chemical shifts are reported in ppm relative to deuterated solvent as internal standard (δ_H_ CDCl_3_ 7.26 ppm; δ_C_ CDCl_3_ 77.16 ppm). Assignments of NMR spectra are based on two-dimensional correlation spectroscopy (COSY, HSQC and HMBC spectra).

### Determination of target specificity

The protease specificity was assessed by testing the inhibition of a series of trypsin-like serine proteases using fluorogenic or chromogenic substrates. Peptide dilutions ranging from 40 μM to 40 nM were prepared for all proteases except thrombin (1 μM to 1 nM). The following final concentrations of human proteases were used: 2.2 nM thrombin (Molecular Innovations), 0.6 nM activated protein C (APC, Molecular Innovations), 0.25 nM plasma kallikrein (Innovative Research), 1 nM factor XIa (Innovative Research), 0.1 nM trypsin (Molecular Innovations) and 5.3 nM factor XIIa (Molecular Innovations). The following fluorogenic substrates were used at a final concentration of 50 μM: Z-Gly-Gly-Arg-AMC (Bachem) for thrombin and trypsin; Z-Phe-Arg-AMC (Bachem) for plasma kallikrein; Boc-Phe-Ser-Arg-AMC (Bachem) for factor XIa; and Boc-Gln-Gly-Arg-AMC (Bachem) for factor XIIa. For APC, the final concentration of 0.48 mM was used for the substrate pyroGlu-Pro-Arg-pNA-HCl (S-2366, Chromogenix, abcr). Reactions were performed in a volume of 150 μl in 96-well plates. A volume of 50 μl of twofold dilutions of macrocycle in assay buffer was pipetted into the wells of the plates. A volume of 50 μl of protease in assay buffer was added to each well and incubated for 15 min. A volume of 50 μl of substrate in assay buffer containing 3% DMSO was added. Fluorescence intensity was measured for 30 min using an Infinite M200 Pro plate reader (Tecan; excitation at 368 nm, emission at 467 nm), with one measurement every minute. For APC protease, absorbance was measured at 405 nm using the same plate reader for a period of 30 min with one reading every minute. Reactions were performed at 25 °C. Data were analyzed by Excel (version 2016).

### Determination of the Michaelis–Menten kinetics

The activity of thrombin was measured using the same buffer, substrate and enzyme concentration and the same instrument as for the specificity profiling. The thrombin activity was measured at substrate concentrations of 15.6, 31.2, 62.5, 125 and 250 μM. The activity was measured in absence of inhibitor and in presence of 50, 150 and 450 nM inhibitory peptide **46**.

### Reporting summary

Further information on research design is available in the Nature Portfolio Reporting Summary linked to this article.

## Online content

Any methods, additional references, Nature Portfolio reporting summaries, source data, extended data, supplementary information, acknowledgements, peer review information; details of author contributions and competing interests; and statements of data and code availability are available at 10.1038/s41589-023-01496-y.

### Supplementary information

Supplementary Information Supplementary Tables 1 and 2, Figs. 1–17 and Notes 1 and 2.

Reporting Summary

### Source data

Source Data Fig. 1 Raw data of Fig. 1.

Source Data Fig. 2 Raw data of Fig. 2.

Source Data Fig. 3 Raw data of Fig. 3.

Source Data Fig. 4 Raw data of Fig. 4.

Source Data Fig. 5 Raw data of Fig. 5.

Source Data Fig. 6 Raw data of Fig. 6.

Source Data Extended Data Fig. 1 Raw data of Extended Data Fig. 1.

Source Data Extended Data Fig. 2 Raw data of Extended Data Fig. 2.

Source Data Extended Data Fig. 3 Raw data of Extended Data Fig. 3.

## Data Availability

Two supplementary tables, 17 supplementary figures and two supplementary notes are provided in Supplementary Information. Raw data shown in graphics in Figs. 1–6 and Extended Data Figs. 1–3 are provided as source data files. Source data are provided with this paper.

## References

[1] Scott DE, Bayly AR, Abell C, Skidmore J (2016). Small molecules, big targets: drug discovery faces the protein–protein interaction challenge. Nat. Rev. Drug Discov..

[2] Anselmo AC, Gokarn Y, Mitragotri S (2019). Non-invasive delivery strategies for biologics. Nat. Rev. Drug Discov..

[3] Pelay-Gimeno M, Glas A, Koch O, Grossmann TN (2015). Structure-based design of inhibitors of protein-protein interactions: mimicking peptide binding epitopes. Angew. Chem. Int. Ed..

[4] Muttenthaler M, King GF, Adams DJ, Alewood PF (2021). Trends in peptide drug discovery. Nat. Rev. Drug Discov..

[5] Nielsen DS (2017). Orally absorbed cyclic peptides. Chem. Rev..

[6] Naylor MR, Bockus AT, Blanco M-J, Lokey RS (2017). Cyclic peptide natural products chart the frontier of oral bioavailability in the pursuit of undruggable targets. Curr. Opin. Chem. Biol..

[7] Drucker DJ (2020). Advances in oral peptide therapeutics. Nat. Rev. Drug Discov..

[8] Räder AFB (2018). Orally active peptides: is there a magic bullet?. Angew. Chem. Int. Ed..

[9] Over B (2016). Structural and conformational determinants of macrocycle cell permeability. Nat. Chem. Biol..

[10] Pye CR (2017). Nonclassical size dependence of permeation defines bounds for passive adsorption of large drug molecules. J. Med. Chem..

[11] Doak BC, Over B, Giordanetto F, Kihlberg J (2014). Oral druggable space beyond the rule of 5: insights from drugs and clinical candidates. Chem. Biol..

[12] White TR (2011). On-resin N-methylation of cyclic peptides for discovery of orally bioavailable scaffolds. Nat. Chem. Biol..

[13] Wang CK (2014). Rational design and synthesis of an orally bioavailable peptide guided by NMR amide temperature coefficients. Proc. Natl Acad. Sci. USA.

[14] Räder AFB, Reichart F, Weinmüller M, Kessler H (2018). Improving oral bioavailability of cyclic peptides by *N*-methylation. Bioorg. Med. Chem..

[15] Bhardwaj, G. et al. Accurate de novo design of membrane-traversing macrocycles. *Cell* **185**, 3520–3532.e26 (2022). 10.1016/j.cell.2022.07.019 PMC9490236 36041435

[16] Deyle K, Kong X-D, Heinis C (2017). Phage selection of cyclic peptides for application in research and drug development. Acc. Chem. Res..

[17] Huang Y, Wiedmann MM, Suga H (2019). RNA display methods for the discovery of bioactive macrocycles. Chem. Rev..

[18] Kong X-D (2020). De novo development of proteolytically resistant therapeutic peptides for oral administration. Nat. Biomed. Eng..

[19] Plais L, Scheuermann J (2022). Macrocyclic DNA-encoded chemical libraries: a historical perspective. RSC Chem. Biol..

[20] Usanov DL, Chan AI, Maianti JP, Liu DR (2018). Second-generation DNA-templated macrocycle libraries for the discovery of bioactive small molecules. Nat. Chem..

[21] Onda Y (2021). A DNA‐encoded chemical library based on peptide macrocycles. Chemistry.

[22] Kale SS (2019). Thiol-to-amine cyclization reaction enables screening of large libraries of macrocyclic compounds and the generation of sub-kilodalton ligands. Sci. Adv..

[23] Sangouard G (2021). Picomole‐scale synthesis and screening of macrocyclic compound libraries by acoustic liquid transfer. Angew. Chem. Int. Ed..

[24] Habeshian S (2022). Synthesis and direct assay of large macrocycle diversities by combinatorial late-stage modification at picomole scale. Nat. Commun..

[25] Coppens M, Eikelboom JW, Gustafsson D, Weitz JI, Hirsh J (2012). Translational success stories. Circ. Res..

[26] Usmani KA, Karoly ED, Hodgson E, Rose RL (2004). In vitro sulfoxidation of thioether compounds by human cytochrome P450 and flavin-containing monooxygenase isoforms with particular reference to the CYP2C subfamily. Drug Metab. Dispos..

[27] Hermann, P. & Morselli, P. L. Pharmacokinetics of diltiazem and other calcium entry blockers. *Acta Pharm. Toxicol.* **57**, 10–20 (1985). 10.1111/j.1600-0773.1985.tb03570.x 3904330

[28] Kale SS (2018). Cyclization of peptides with two chemical bridges affords large scaffold diversities. Nat. Chem..

[29] Stangier J, Clemens A (2009). Pharmacology, pharmacokinetics, and pharmacodynamics of dabigatran etexilate, an oral direct thrombin inhibitor. Clin. Appl. Thromb. Hemost..

[30] Gustafsson D (2004). A new oral anticoagulant: the 50-year challenge. Nat. Rev. Drug Discov..

[31] Roehrig S (2005). Discovery of the novel antithrombotic agent 5-chloro-N-({(5S)-2-oxo-3-[4-(3-oxomorpholin-4-yl)phenyl]-1,3-oxazolidin-5-yl}methyl)thiophene-2-carboxamide (BAY 59-7939): an oral, direct factor Xa inhibitor. J. Med. Chem..

[32] Lipinski CA, Lombardo F, Dominy BW, Feeney PJ (2001). Experimental and computational approaches to estimate solubility and permeability in drug discovery and development settings. Adv. Drug Deliv. Rev..

[33] Wang J, Yadav V, Smart AL, Tajiri S, Basit AW (2015). Toward oral delivery of biopharmaceuticals: an assessment of the gastrointestinal stability of 17 peptide drugs. Mol. Pharm..

[34] Kansy M, Senner F, Gubernator K (1998). Physicochemical high throughput screening: parallel artificial membrane permeation assay in the description of passive absorption processes. J. Med. Chem..

[35] Scott KA, Njardarson JT (2018). Analysis of US FDA-approved drugs containing sulfur atoms. Top. Curr. Chem..

[36] Golosov AA (2021). Design of thioether cyclic peptide scaffolds with passive permeability and oral exposure. J. Med. Chem..

[37] Jacobsen Ø, Klaveness J, Rongved P (2010). Structural and pharmacological effects of ring-closing metathesis in peptides. Molecules.

[38] Walensky LD, Bird GH (2014). Hydrocarbon-stapled peptides: principles, practice, and progress. J. Med. Chem..

[39] Lau YH, de Andrade P, Wu Y, Spring DR (2015). Peptide stapling techniques based on different macrocyclisation chemistries. Chem. Soc. Rev..

[40] Cromm PM, Spiegel J, Grossmann TN (2015). Hydrocarbon stapled peptides as modulators of biological function. ACS Chem. Biol..

[41] Rupin A (2011). S35972, a direct-acting thrombin inhibitor with high oral bioavailability and antithrombotic efficacy. J. Thromb. Haemost..

[42] Veber DF (2002). Molecular properties that influence the oral bioavailability of drug candidates. J. Med. Chem..

