# Supplementary material for: De novo development of small cyclic peptides that are orally bioavailable
Source: Nat Chem Biol. 2023 Dec 28;20(5):624–33. doi: 10.1038/s41589-023-01496-y (PMC11062899; doi:10.1038/s41589-023-01496-y)
Supplement: Supplementary file 1 — Supplementary Tables 1 and 2, Figs. 1–17 and Notes 1 and 2. [file 41589_2023_1496_MOESM1_ESM.pdf]

nature chemical biology

Article

https://doi.org/10.1038/s41589-023-01496-y

De novo development of small cyclic peptides that are orally bioavailable

In the format provided by the authors and unedited

Table of Contents

Supplementary Figures

Supplementary Figure 1. Physico-chemical properties of cyclic peptides 3

Supplementary Figure 2. Metabolic stability of dithioether cyclic peptides in rat liver microsomes 4

Supplementary Figure 3. Identification of oxidation sites 11

Supplementary Figure 4. LC-MS purity of linear dithiol peptides 12

Supplementary Figure 5. Cyclization of dithiol peptides with bis-electrophilic linker reagents 14

Supplementary Figure 6. *N*-acylation of cyclic peptides using acoustic dispensing 16

Supplementary Figure 7. Structure-activity relationship (SAR) of 20 thrombin inhibitors 21

Supplementary Figure 8. Characterization of thrombin inhibitors 23

Supplementary Figure 9. Physico-chemical properties of cyclic peptides of sub-library based on peptide 11 33

Supplementary Figure 10. Characterization of thrombin inhibitors from the sub-library 34

Supplementary Figure 11. Oxidation of peptide 32 by liver microsome treatment 38

Supplementary Figure 12. Characterization of ring-closing metathesis (RCM) thrombin inhibitors 39

Supplementary Figure 13. Stability of cyclic peptide 43 41

Supplementary Figure 14. Characterization and oral availability of the cyclic peptide 1NMe3 42

Supplementary Figure 15. Characterization of RCM thrombin inhibitors with diverse *N*-acylation 44

Supplementary Figure 16. Stability of RCM thrombin inhibitors with diverse N-acylation 45

Supplementary Figure 17. Binding mode of peptide 46 46

Supplementray Tables

Supplementary Table 1. Cyclic peptide high-throughput screen 47

Supplementary Table 2. In vitro properties of peptides and reference compounds 48

Supplementray Notes

Supplementary Note 1. NMR spectra of peptide 46 49

Supplementary Note 2. High resolution masses of peptides 11, 23, 43 and 46 52

Supplementary Figures

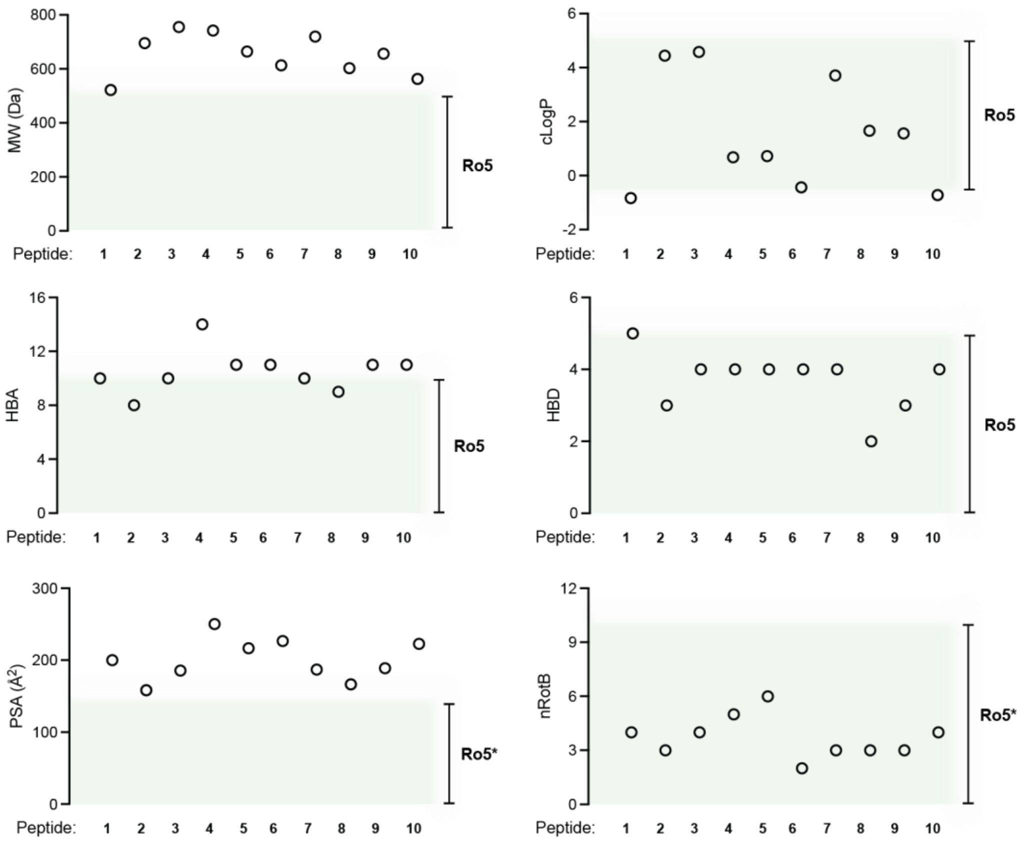

Supplementary Fig. 1 | Physico-chemical properties of cyclic peptides. DataWarrior software was used to calculate the physico-chemical properties. Space defined by Lipinski's Rule of 5 (Ro5) is highlighted in green.[1] For PSA and nRotB that were not defined by Lipinski *et al*., Ro5* indicates the range that is defined by Veber *et al.*[2]. MW represents the molecular weight, cLogP the calculated lipophilicity (octanol/water partition coefficient), HBA the number of hydrogen bond acceptors, HBD the number of hydrogen bond donors, PSA the polar surface area, and nRotB the number of rotatable bonds.

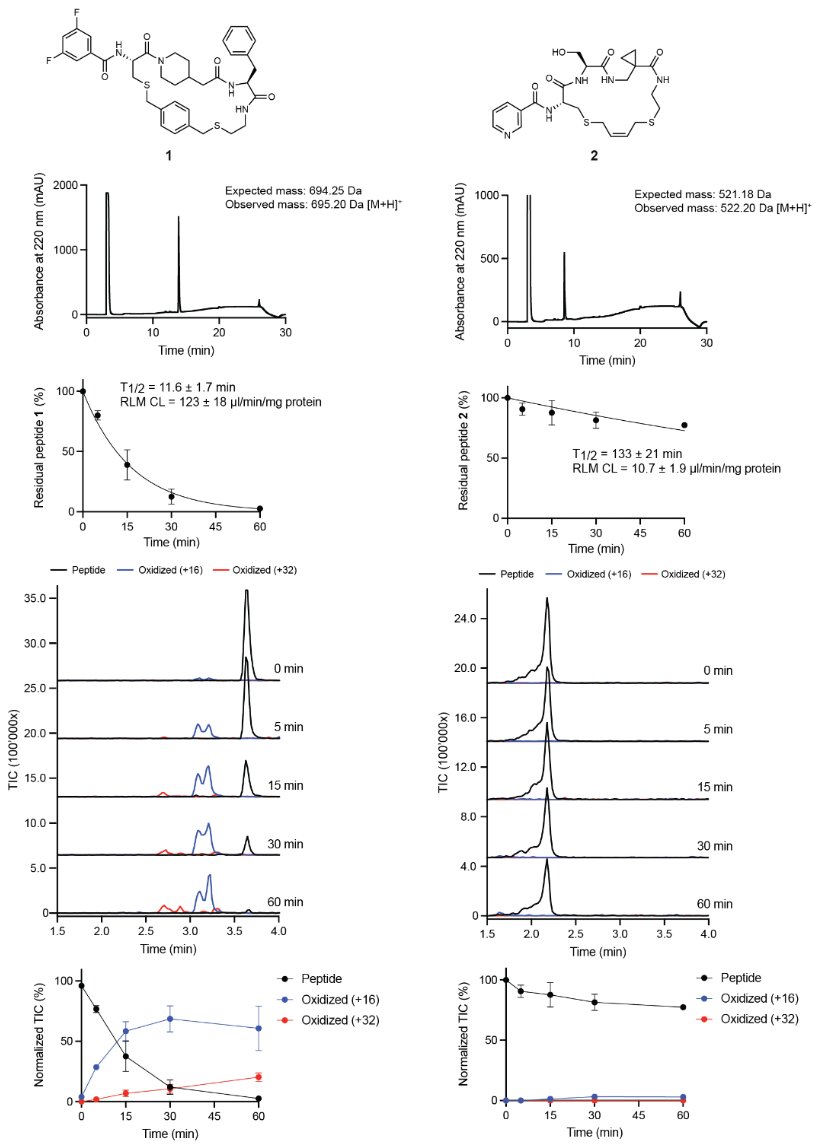

Supplementary Fig. 2 | Metabolic stability of dithioether cyclic peptides in rat liver microsomes. Chemical structures of cyclic peptides and reference drugs are shown on top with their respective RP-HPLC chromatograms below. Metabolic stability was measured by incubating cyclic peptides with rat liver microsomes and analyzing samples at different time points. Half-live ($T_{1/2}$) and clearance (RLM CL) for each cyclic peptide are indicated on the graph. Individual TIC chromatograms acquired at each time point were stacked, whereas the black profile is representing the extracted mass of the intact peptide, and the blue and red profiles represent the metabolized products. Curves with normalized TIC values of a single representative replicate highlighting the disappearance of intact analytes (black curve) and emerging of metabolized products (red and blue curves) are shown on the bottom. Mean values and SDs from three independent measurements are shown for the metabolic stability.

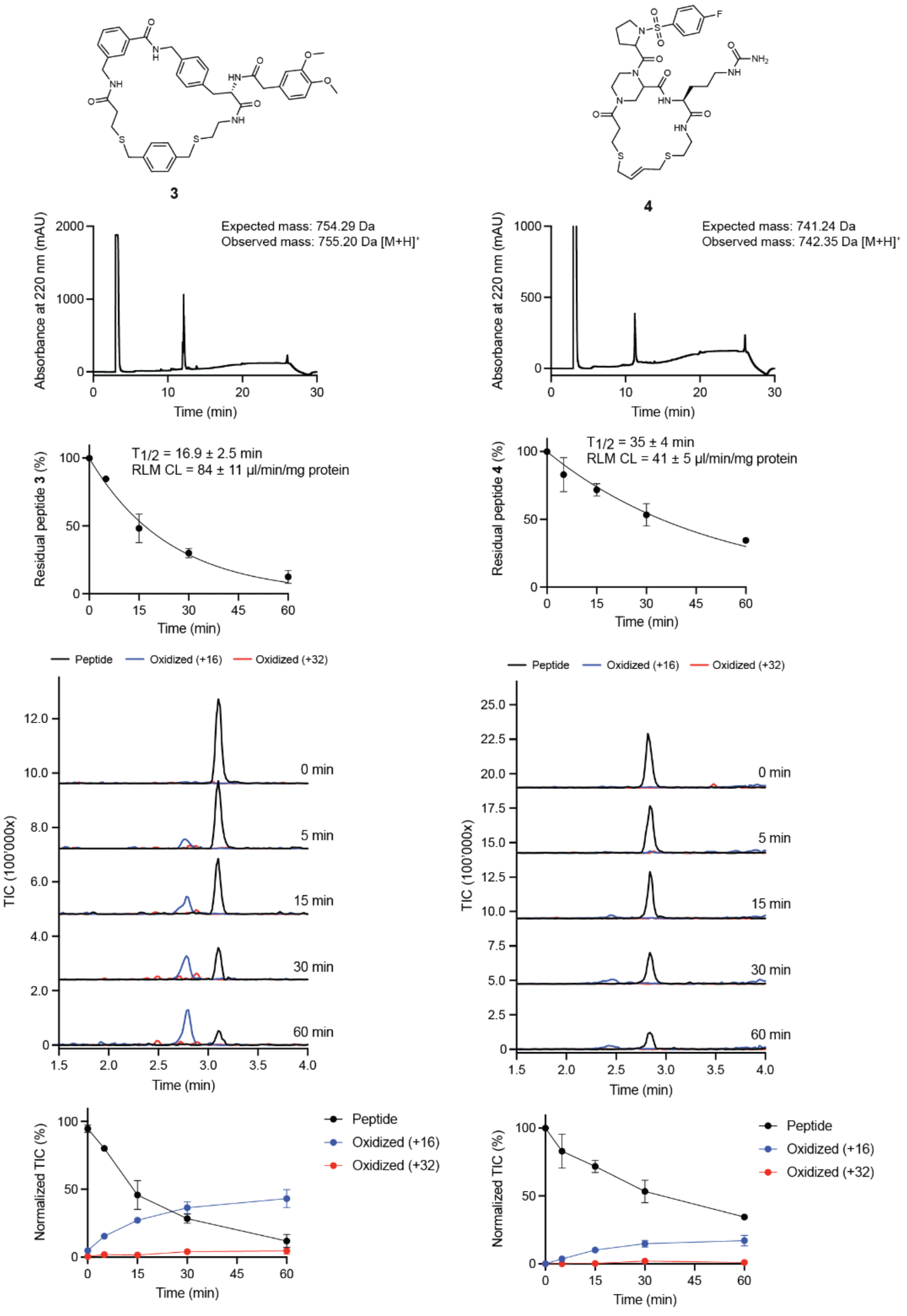

Supplementary Fig. 2 | Continued

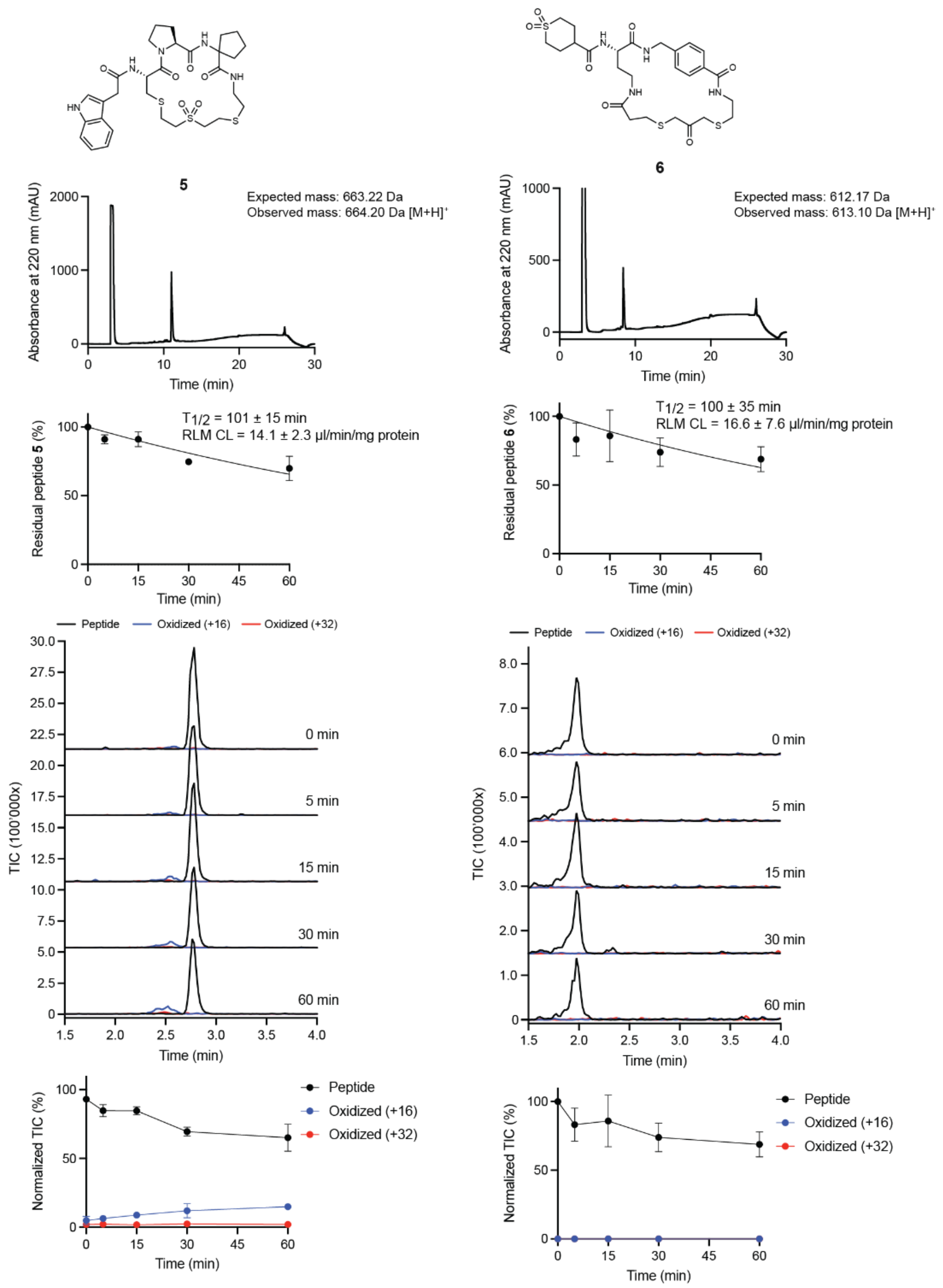

Supplementary Fig. 2 | Continued

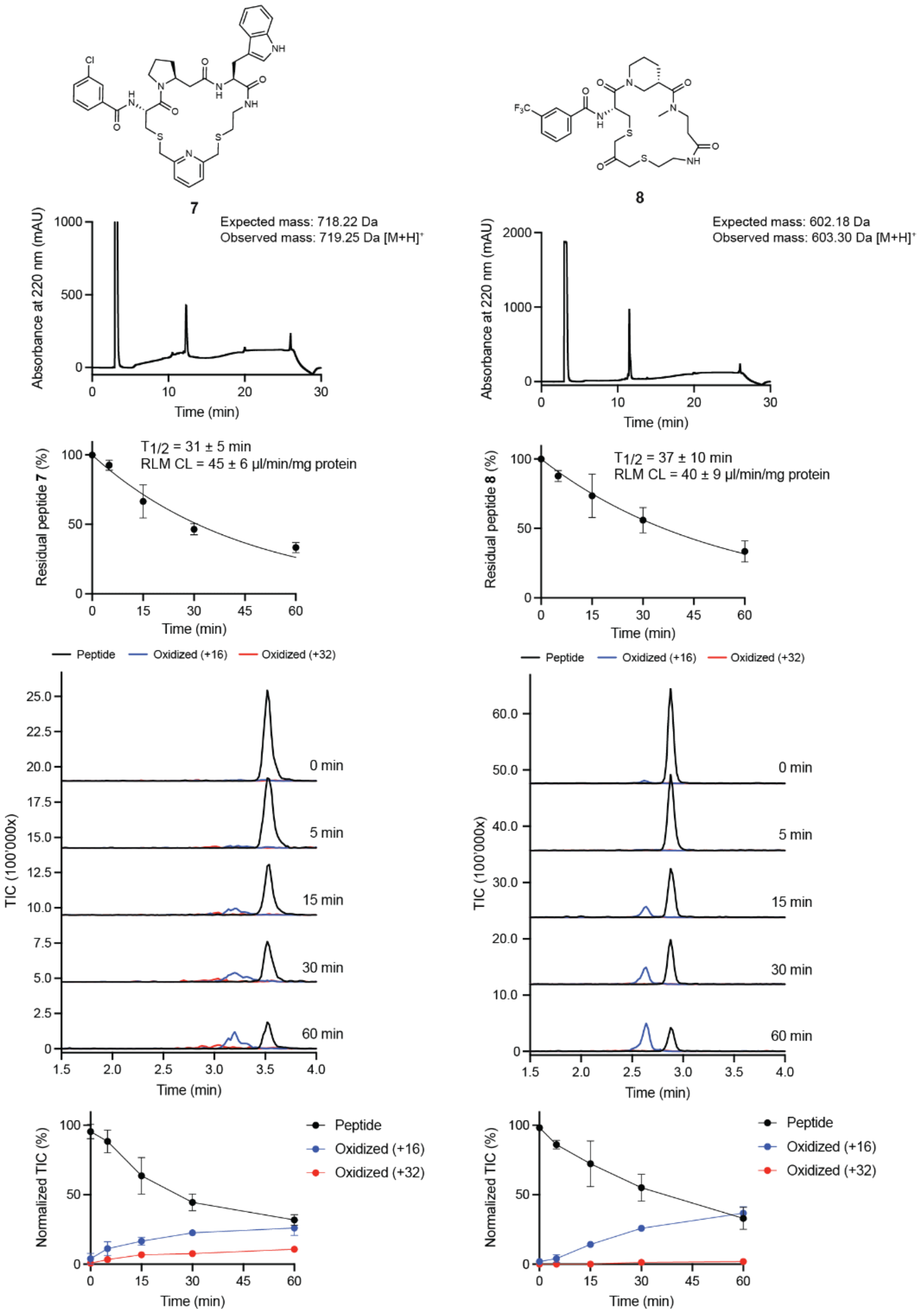

Supplementary Fig. 2 | Continued

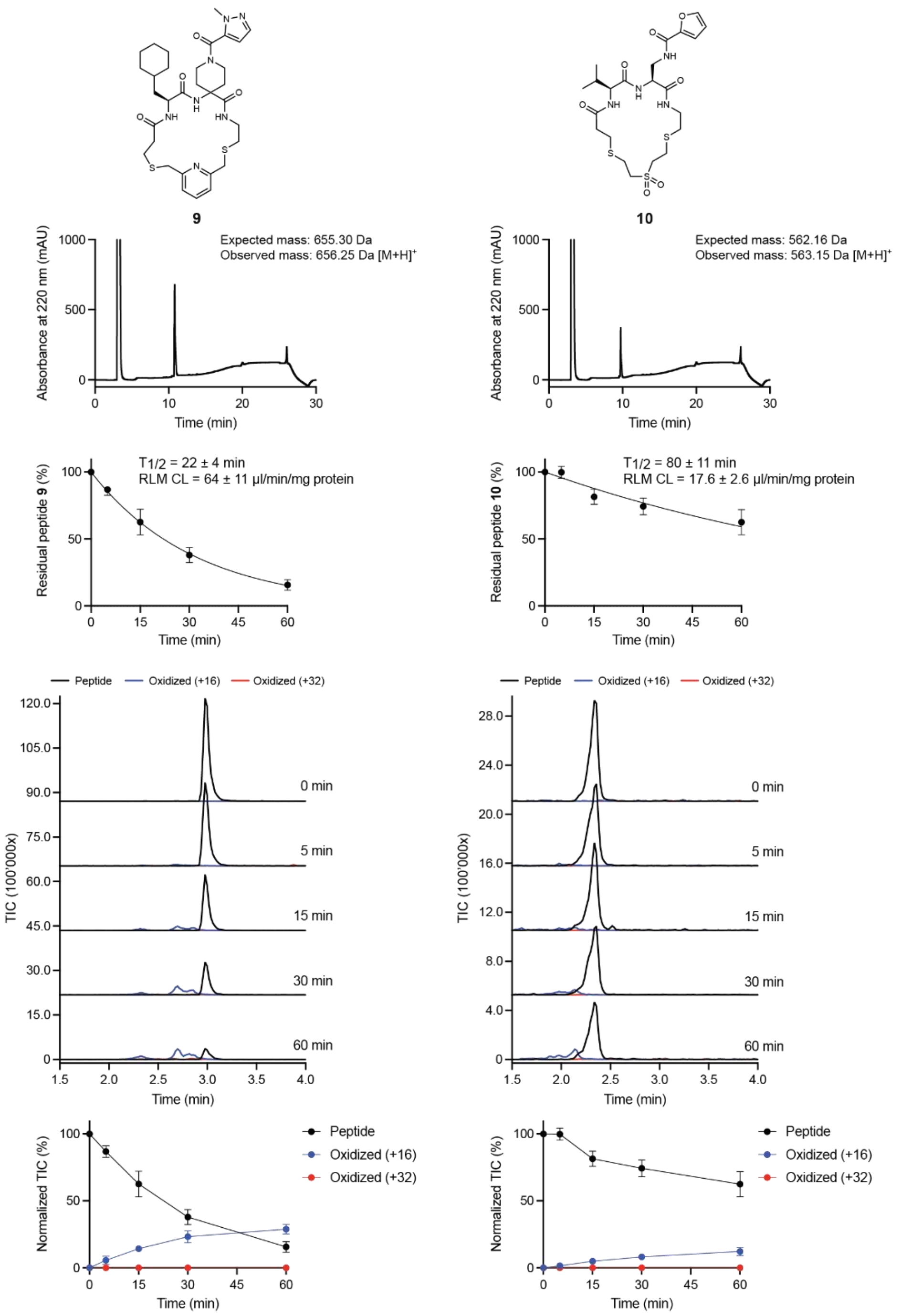

Supplementary Fig. 2 | Continued

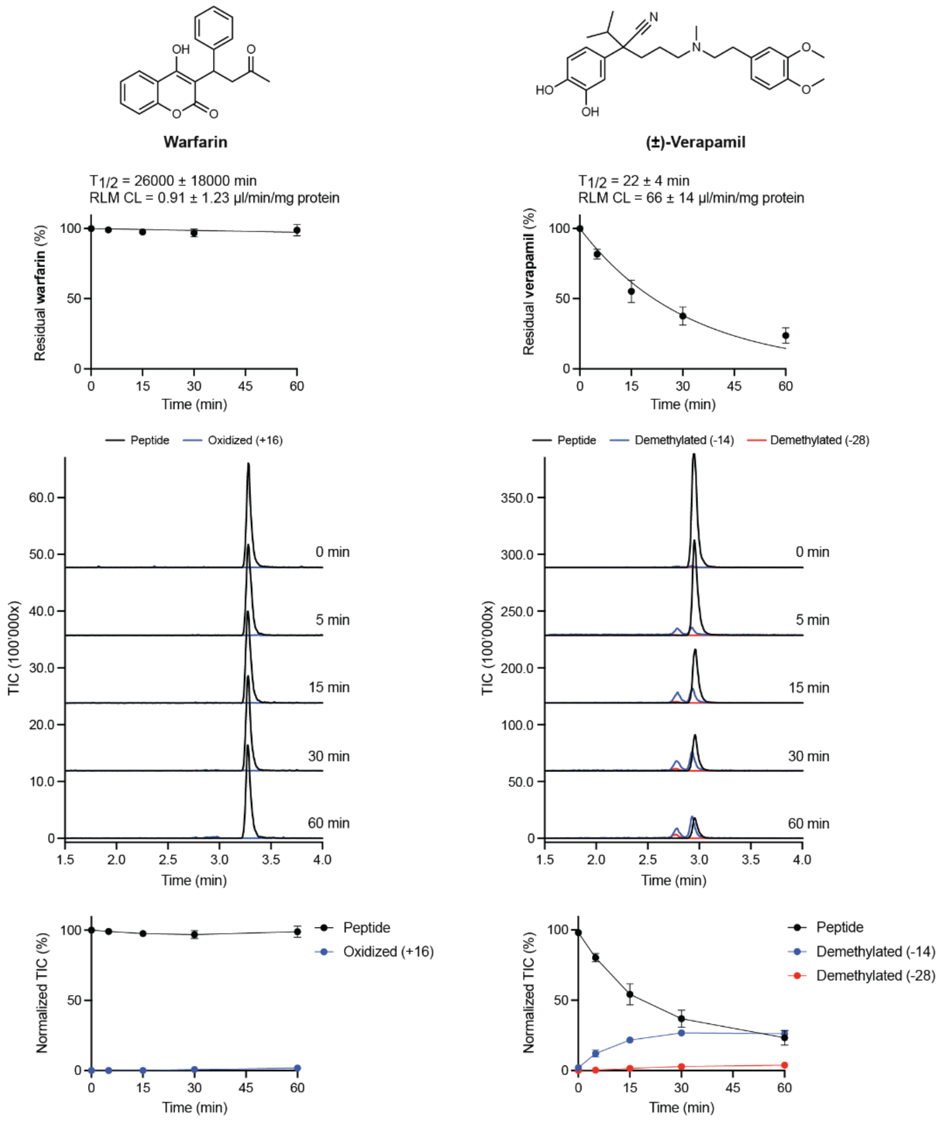

Supplementary Fig. 2 | Continued

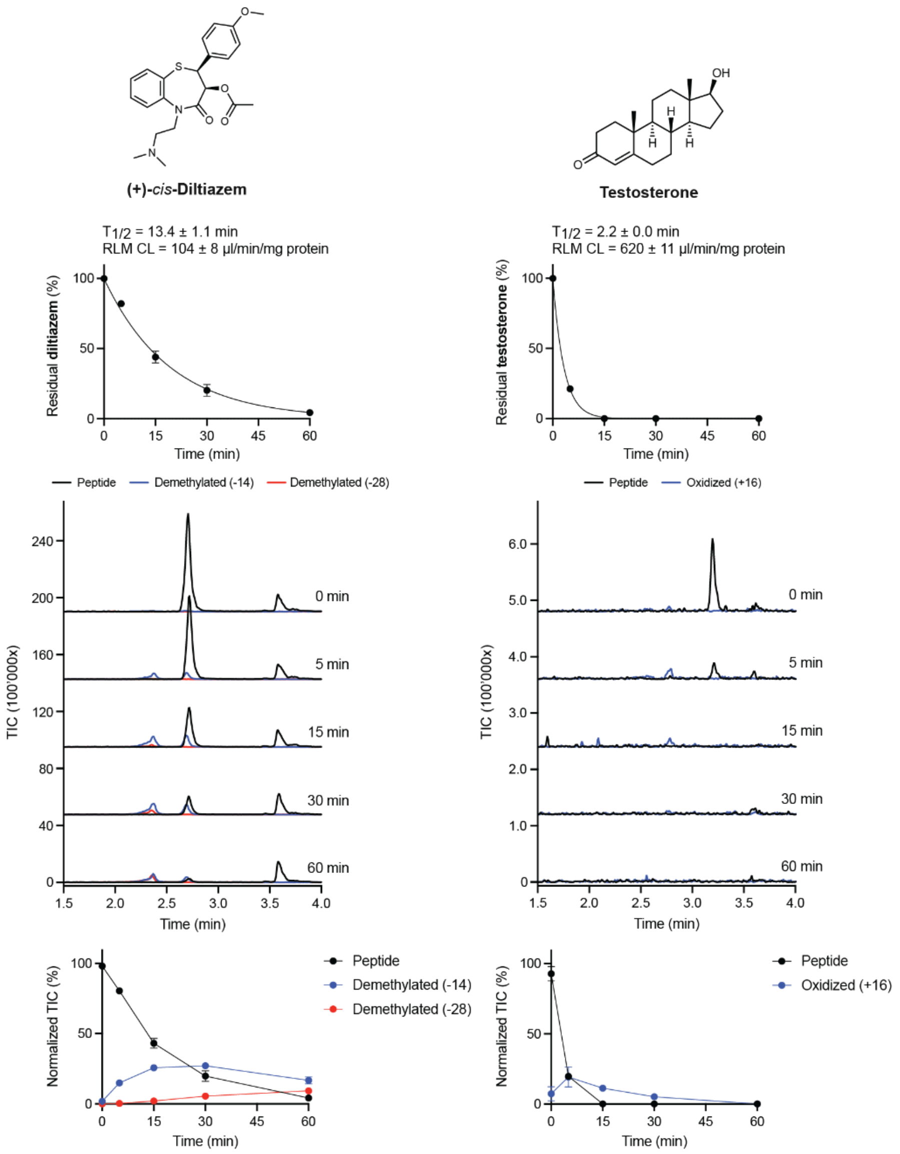

Supplementary Fig. 2 | Continued

a

Peptide	Proteolytic cleavage efficiency: Proteinase K	Chymotrypsin
**1**	26%	97%
**2**	< 1%	0%
**3**	0%	0%
**4**	0%	0%
**5**	2%	0%
**6**	0%	0%
**7**	5%	40%
**8**	0%	0%
**9**	0%	0%
**10**	< 1%	0%

b

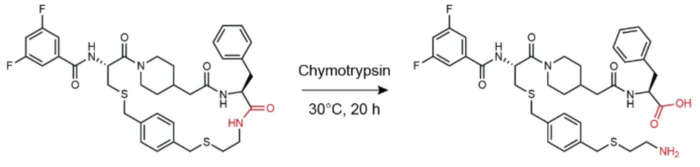

1 **1** (linearized)

c

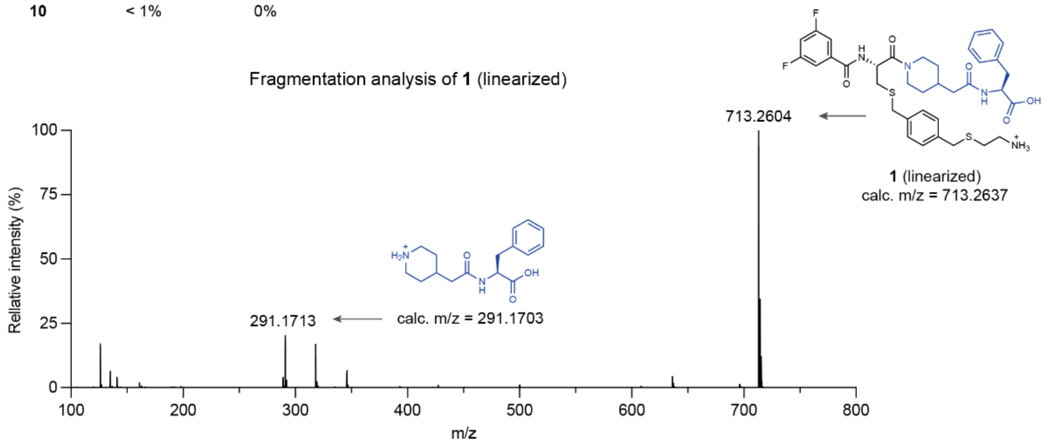

d

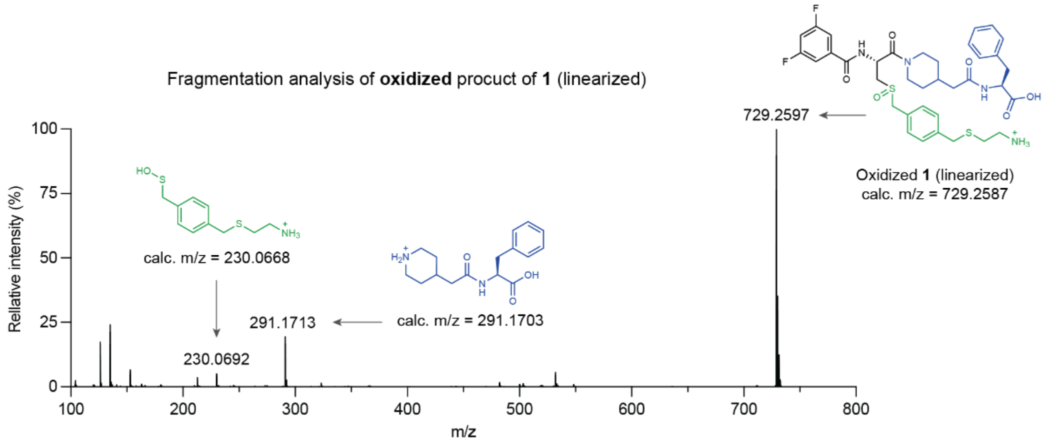

Supplementary Fig. 3 ⎹ Identification of oxidation sites. a, Cleavage and linearization of peptides by proteases. Peptides were incubated either with proteinase K or chymotrypsin in Tris-HCl buffer at pH 8.0 and incubated for 20 h at 37°C or 30°C. Proteolytic cleavage efficiency was measured by analyzing the TIC ratio of linear to cyclic peptide by LC-MS. **b,** Proteolytic cleavage of peptide 1 with chymotrypsin. The sample was incubated overnight for 20 h at 30°C with chymotrypsin to yield >90% linearized peptide 1. **c**, **d,** HR-MS/MS analysis of linearized peptide 1 (**c**) and its oxidized product (**d**) after rat liver microsome incubation. Proposed fragments and their respective calculated and measured masses are directly shown on the MS spectra.

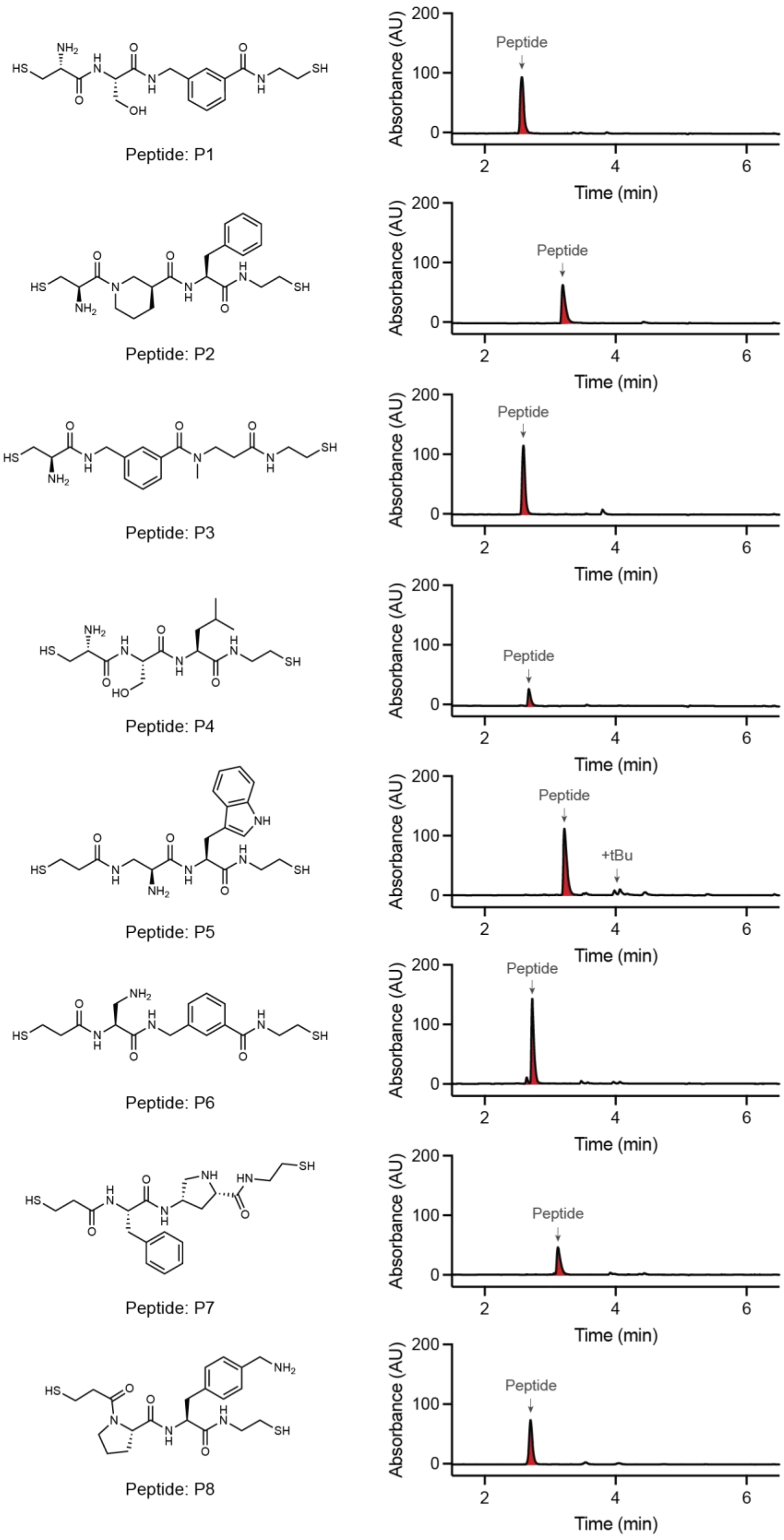

Supplementary Fig. 4 │LC-MS purity of linear dithiol peptides. Peptides were synthesized by automated solid-phase peptide synthesis and cleaved from the resin by adding TFA. After

ether precipitation workup, the peptide purity was analyzed by LC-MS (PDA at 220 nm) using a 0-60% acetonitrile/water (v/v) gradient over 5 min. Peptide product is shown in red color.

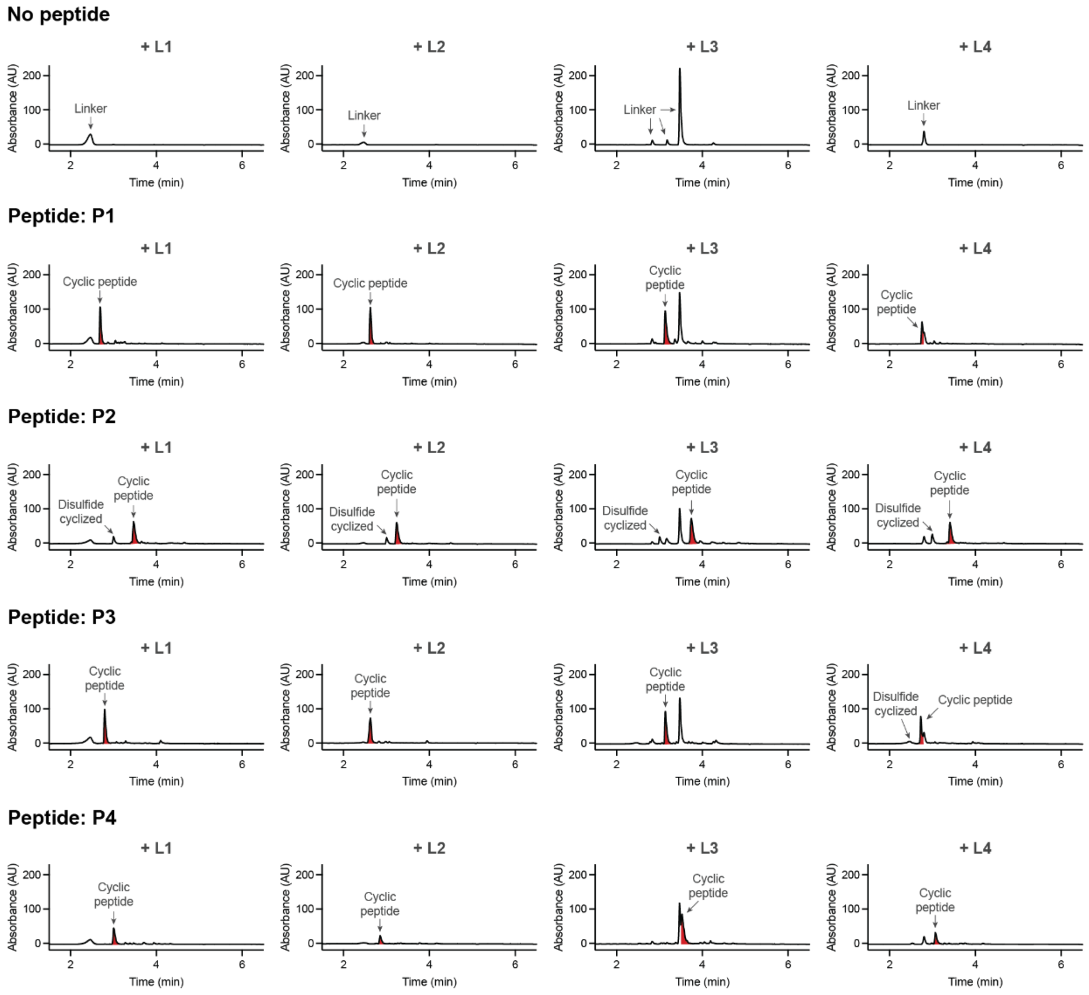

Supplementary Fig. 5 | Cyclization of dithiol peptides with bis-electrophilic linker reagents. Reactions were analyzed by LC-MS (PDA 220 nm) using a 0-60% acetonitrile/water (v/v) gradient over 5 min. Cyclic peptide products (red color) and major side products are shown on the chromatogram. Dashed red color indicates overlapping cyclic peptide and quenched crosslinker peaks. Whenever the species could not be identified, the respective mass was indicated instead.

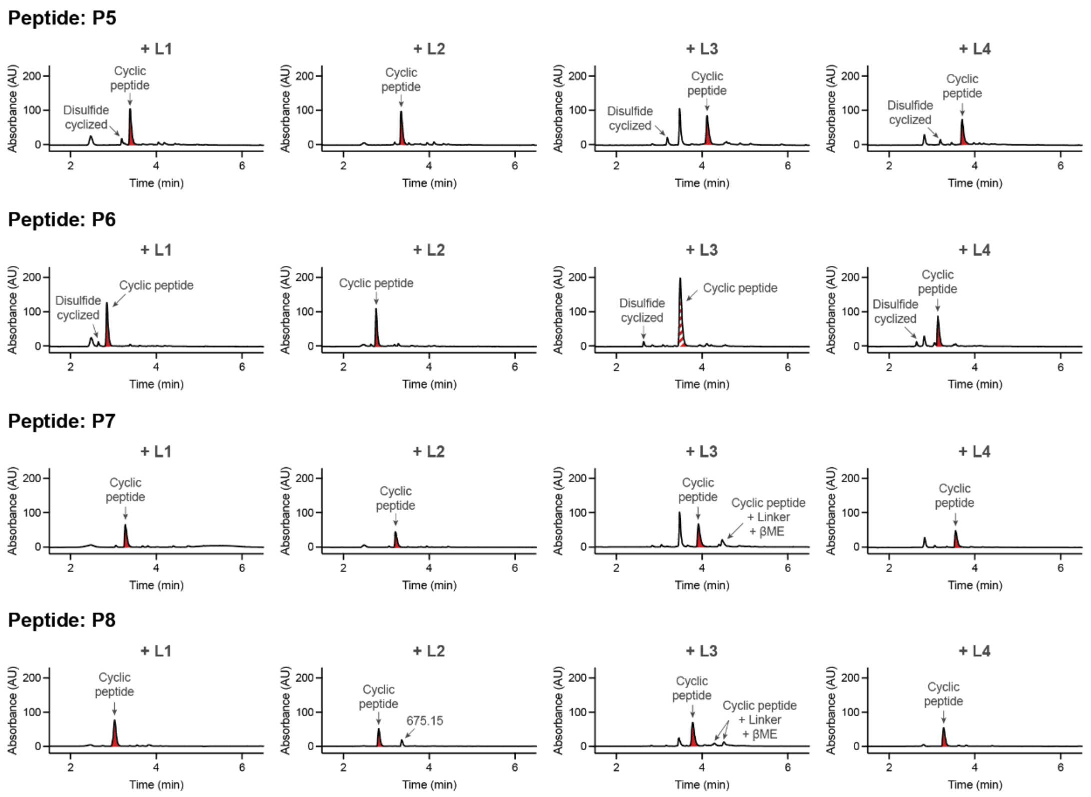

Supplementary Fig. 5 | Continued

No peptide

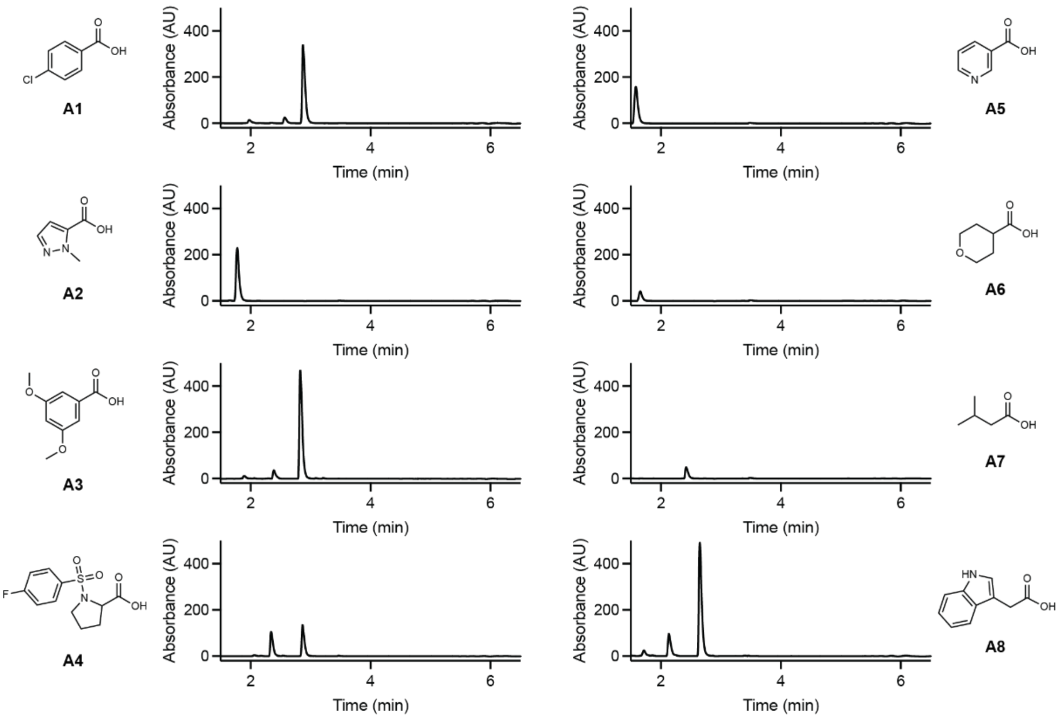

Supplementary Fig. 6 ⎸*N*-acylation of cyclic peptides using acoustic dispensing. Cyclic peptides with peripheral amines were incubated for 8 h with HSTU-activated carboxylic acids and reactions analyzed by LC-MS using a 10-100% acetonitrile/water (v/v) gradient over 5 min. *N*-acylated peptide products are colored in red and cyclic peptides that did not acylate are indicated with “-NH_2”.

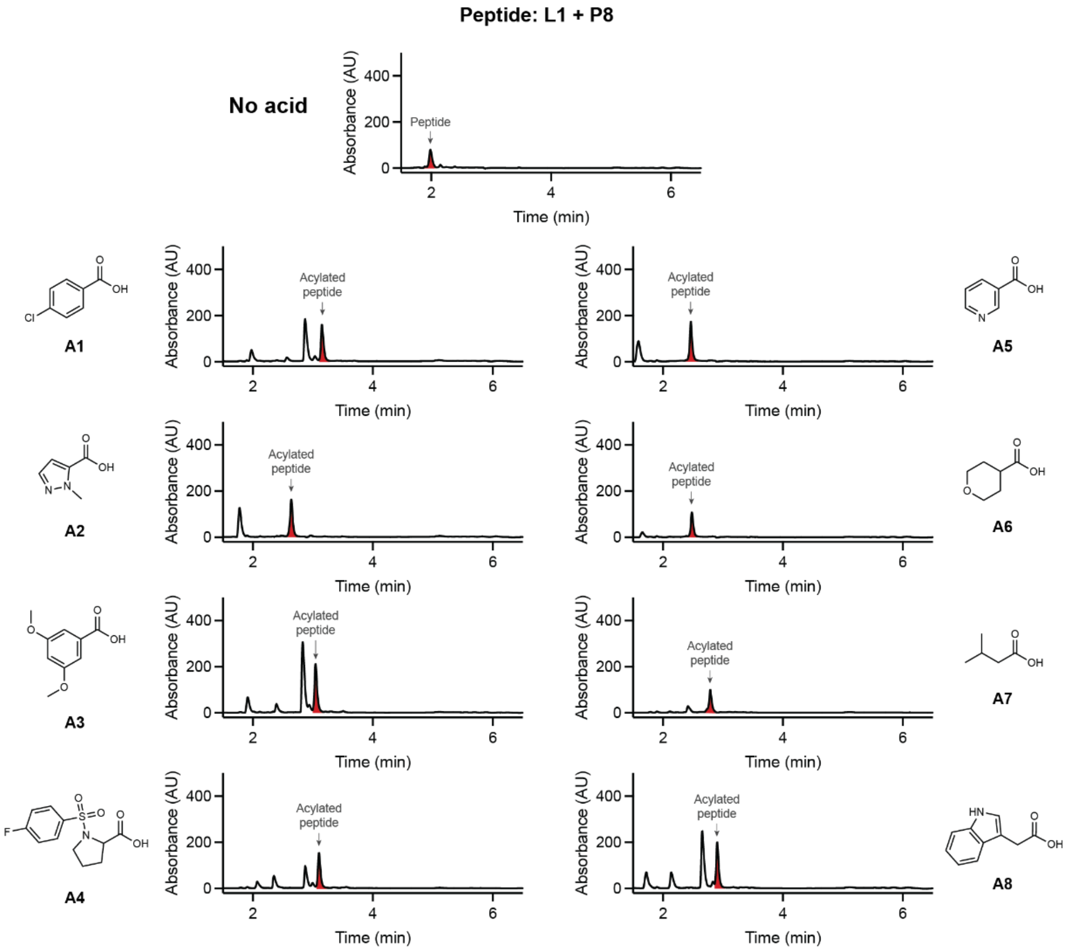

Supplementary Fig. 6 | Continued

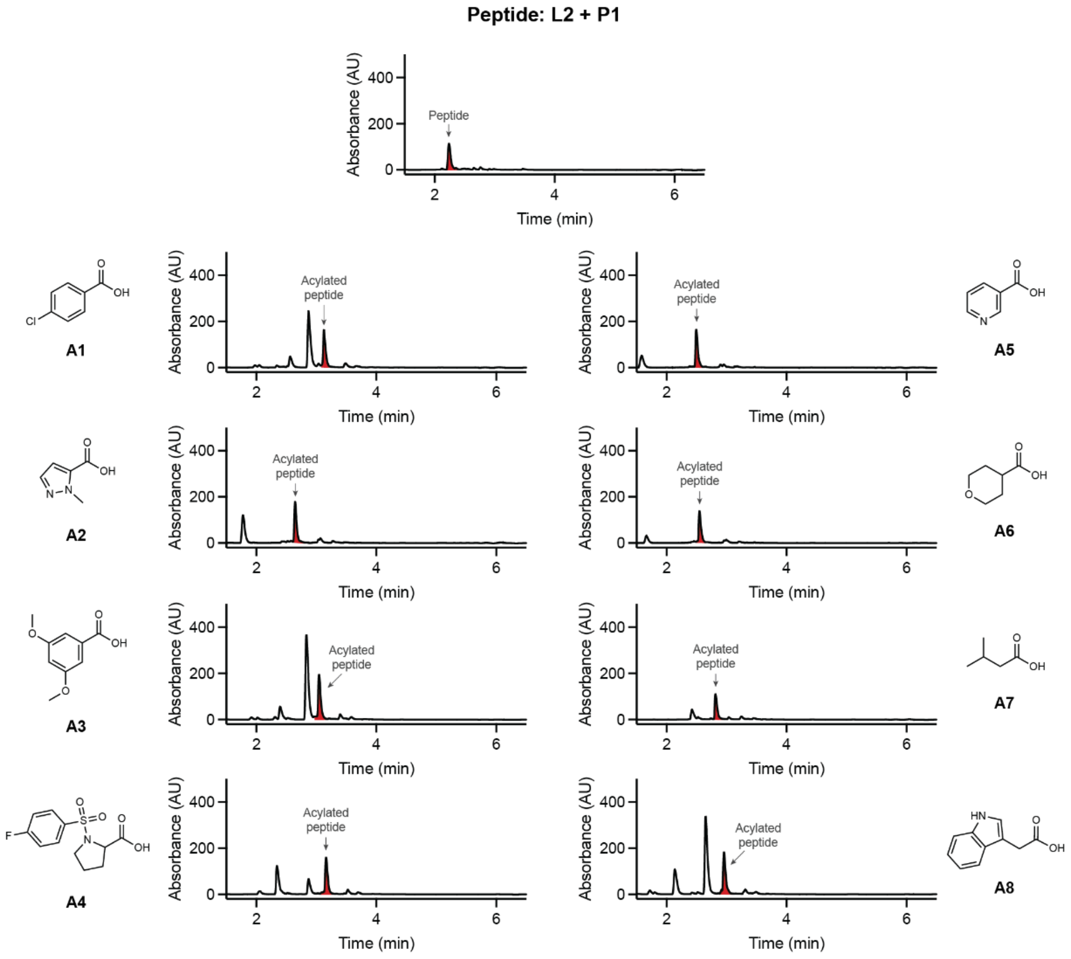

Supplementary Fig. 6 | Continued

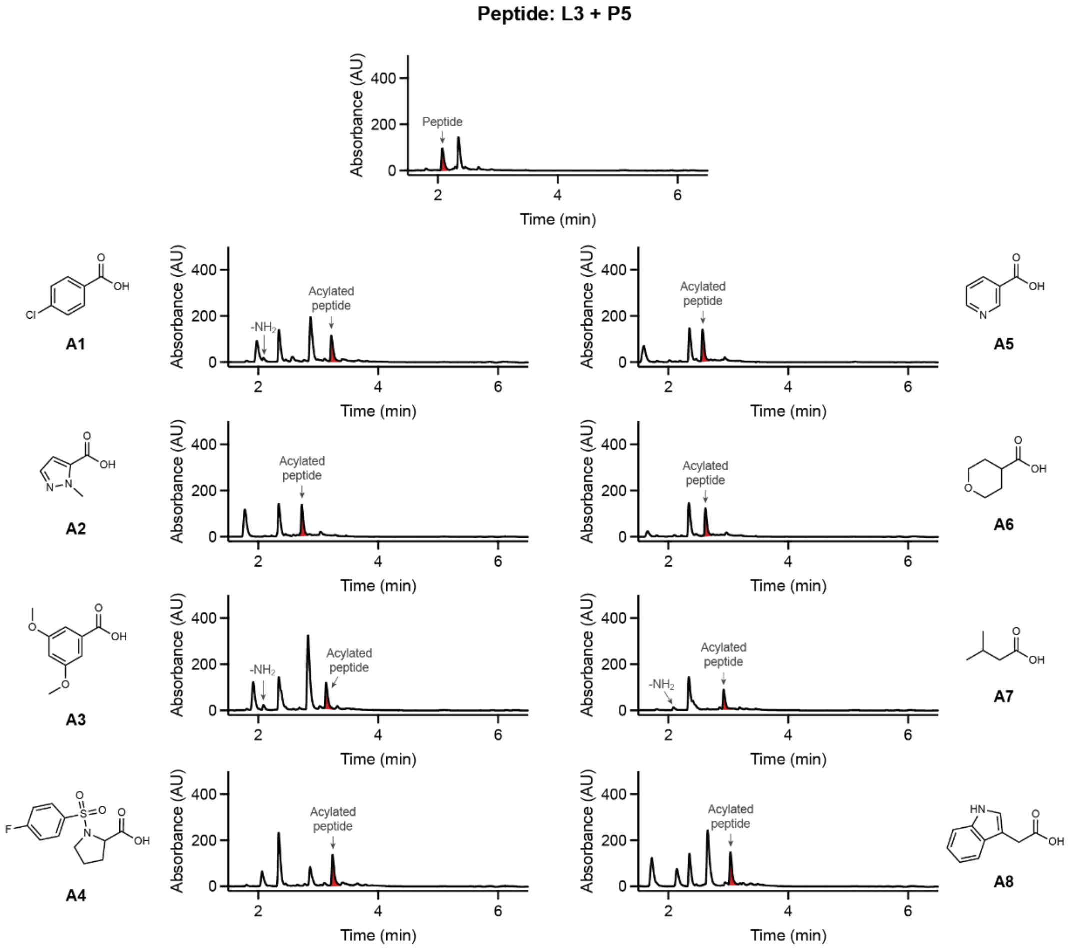

Supplementary Fig. 6 | Continued

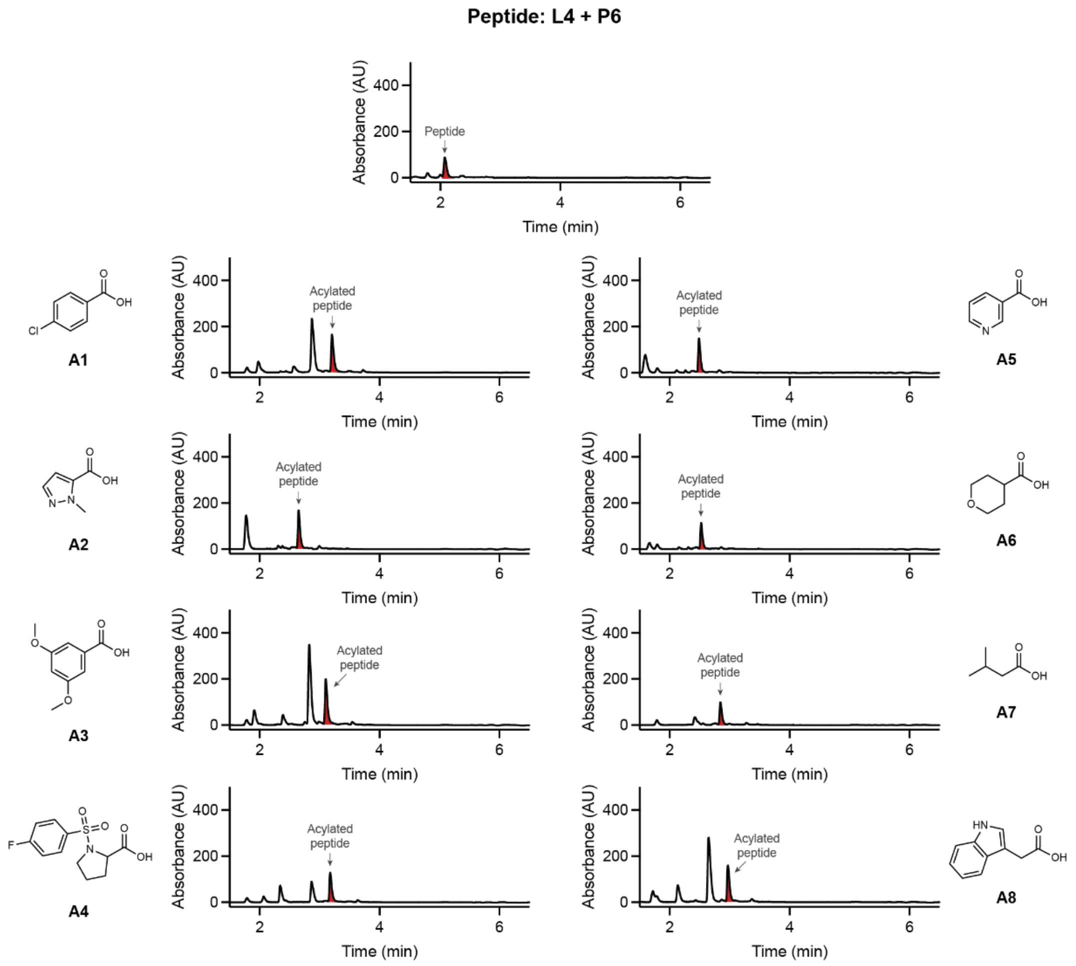

Supplementary Fig. 6 | Continued

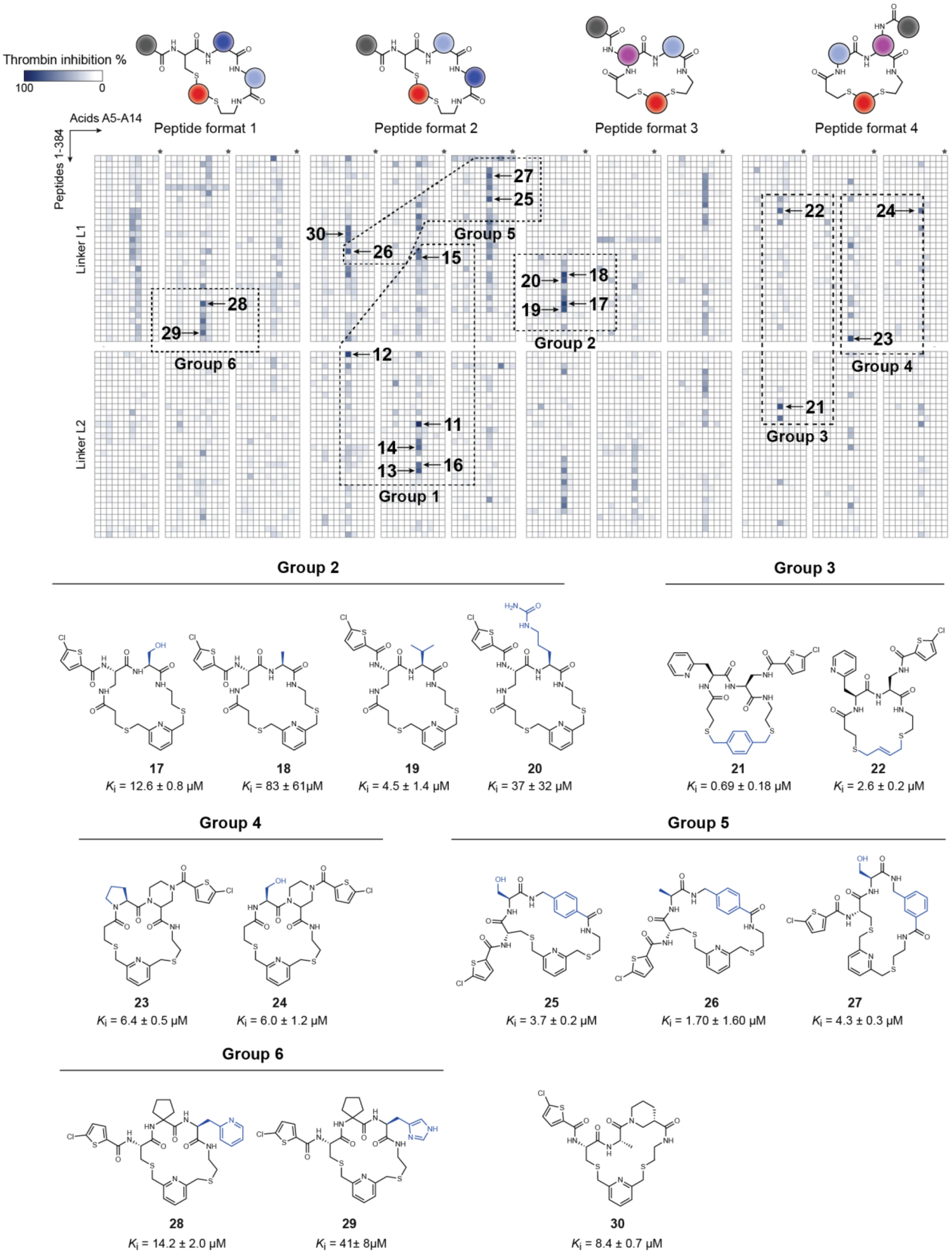

Supplementary Fig. 7 | Structure-activity relationship (SAR) of 20 thrombin inhibitors. Groups 2 – 6 represent the different clusters identified on the heat map. Structures of the cyclic

peptide scaffolds are shown below, with the common core structure of each group highlighted in black.

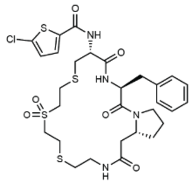

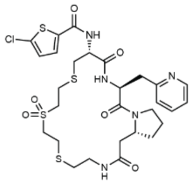

11 **12**

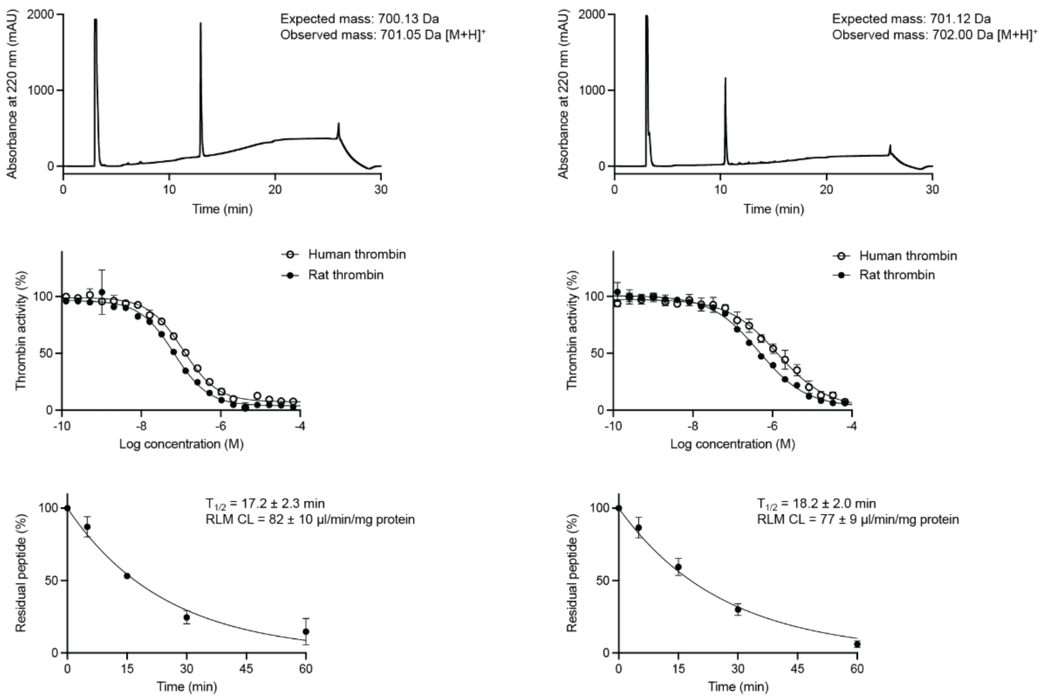

Supplementary Fig. 8 │Characterization of thrombin inhibitors. Chemical structures, RP-HPLC chromatograms, IC_{50} profiles, and microsomal stability curves in rat liver microsomes (top to bottom) for cyclic peptides **11—30** are shown. Mean values and SDs of the thrombin inhibition and metabolic stability were measured in three independent measurements.

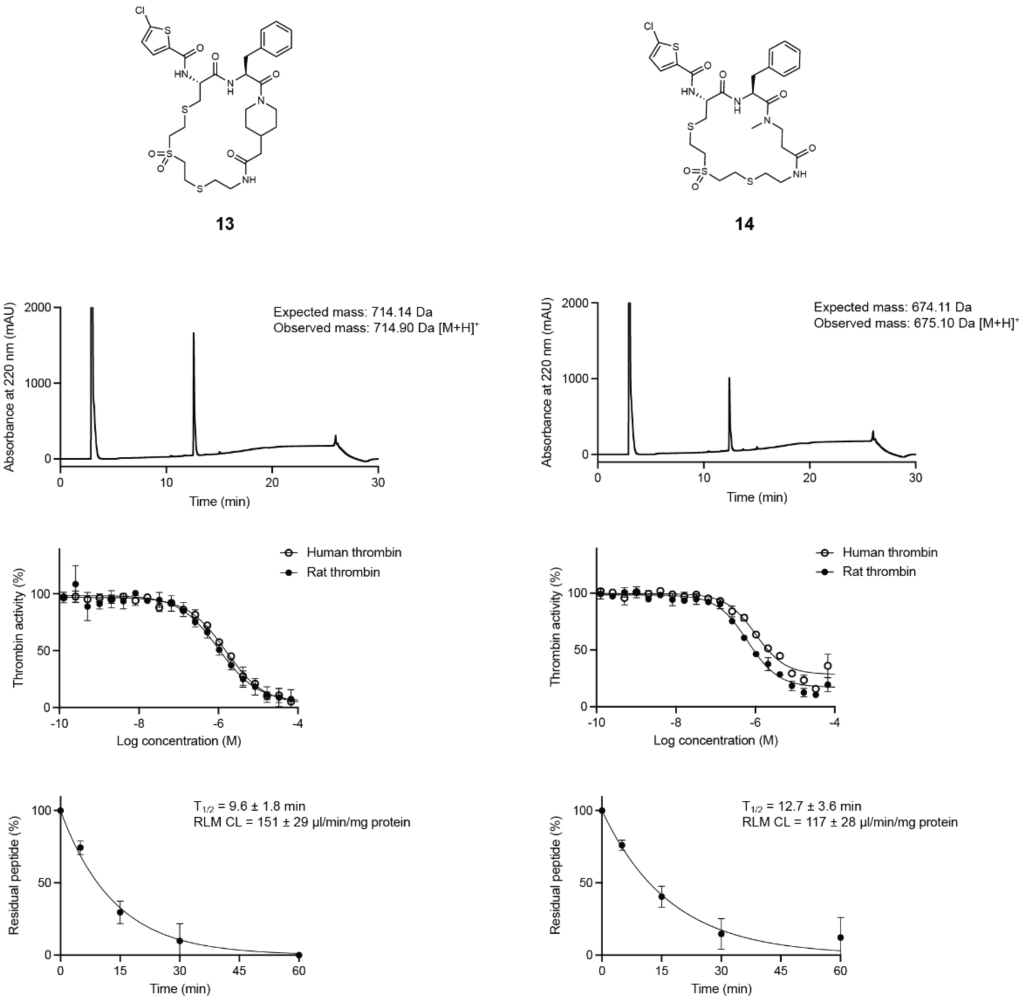

Supplementary Fig. 8 | Continued

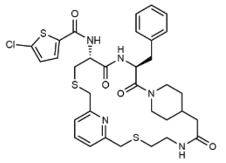

15

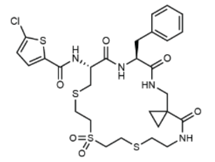

16

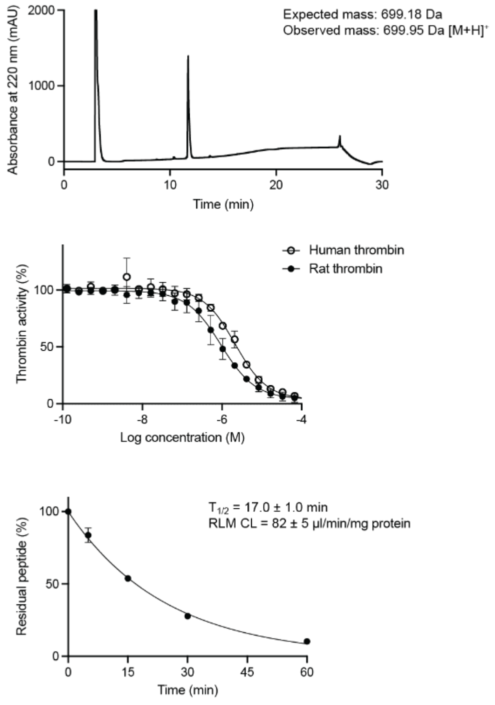

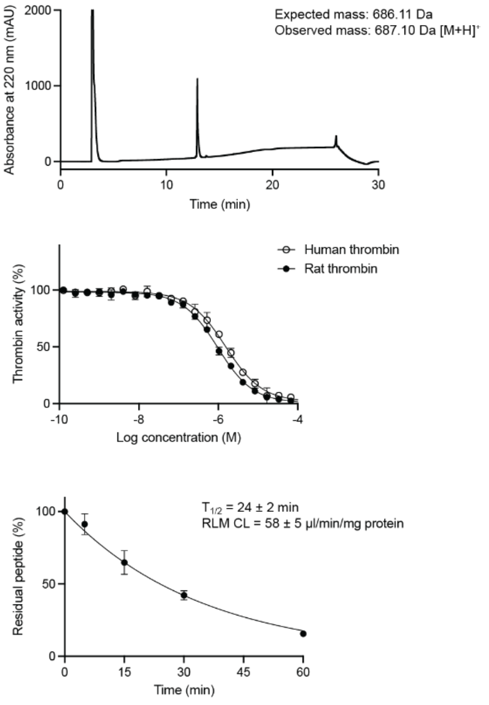

Supplementary Fig. 8 | Continued

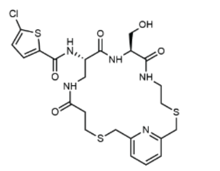

17

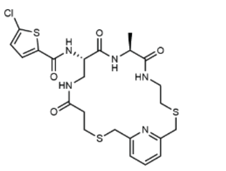

18

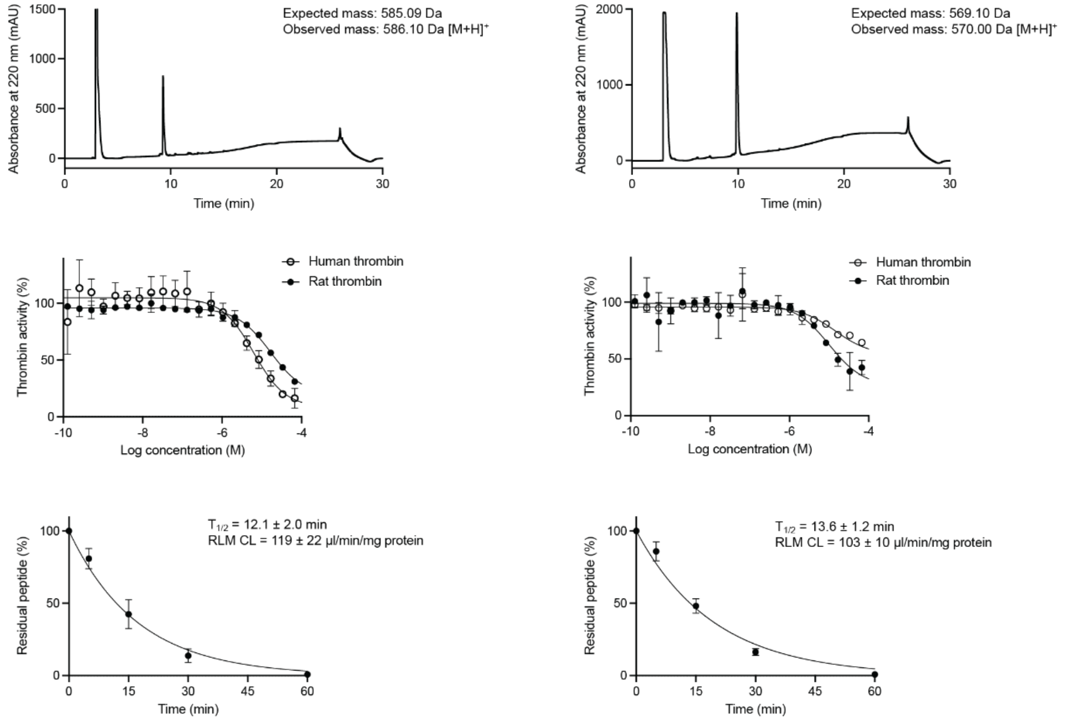

Supplementary Fig. 8 | Continued

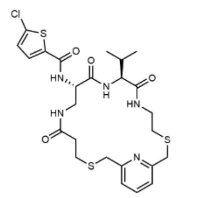

19

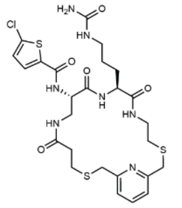

20

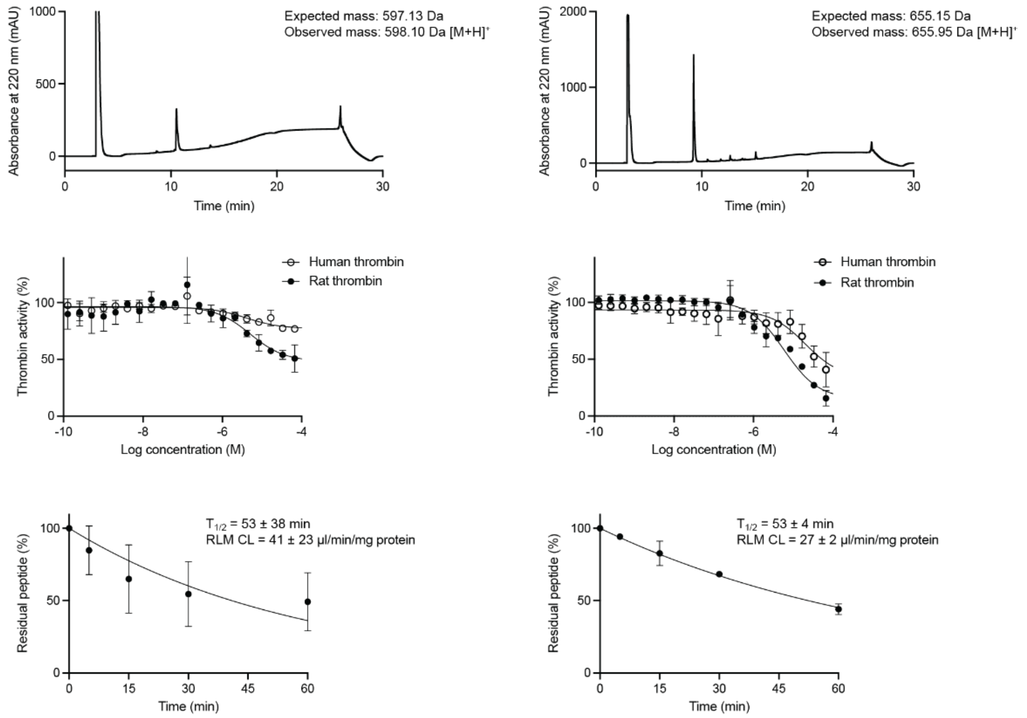

Supplementary Fig. 8 | Continued

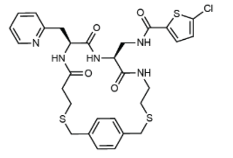

21

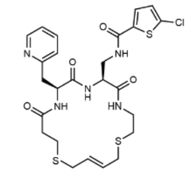

22

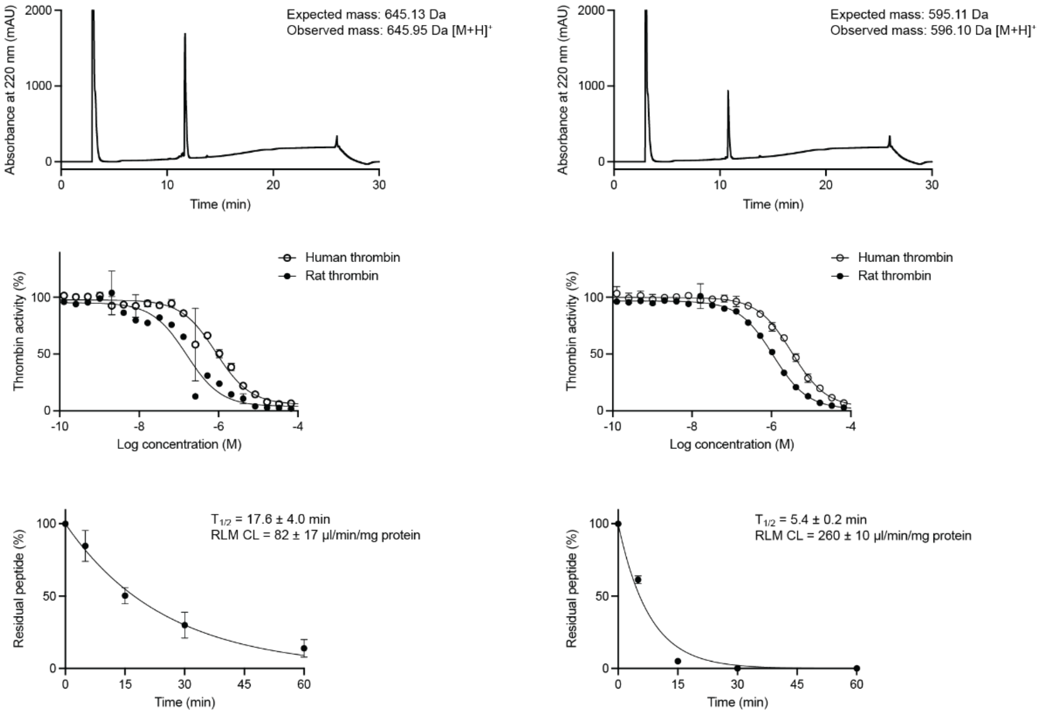

Supplementary Fig. 8 | Continued

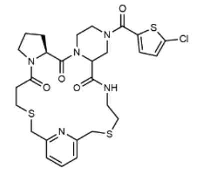

23

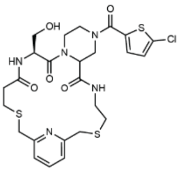

24

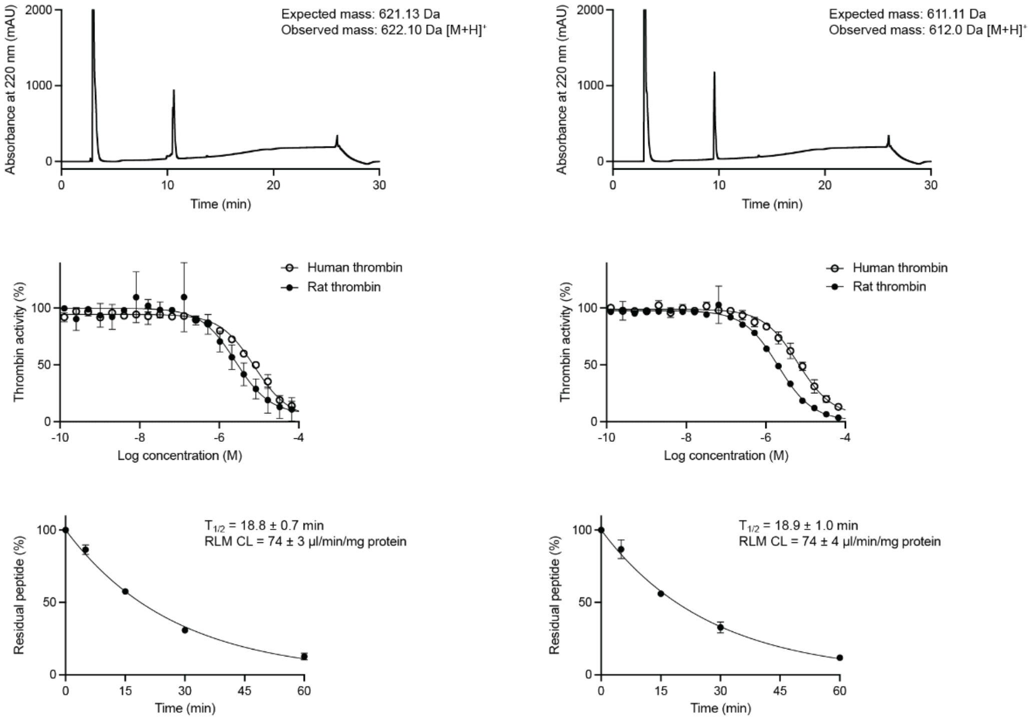

Supplementary Fig. 8 | Continued

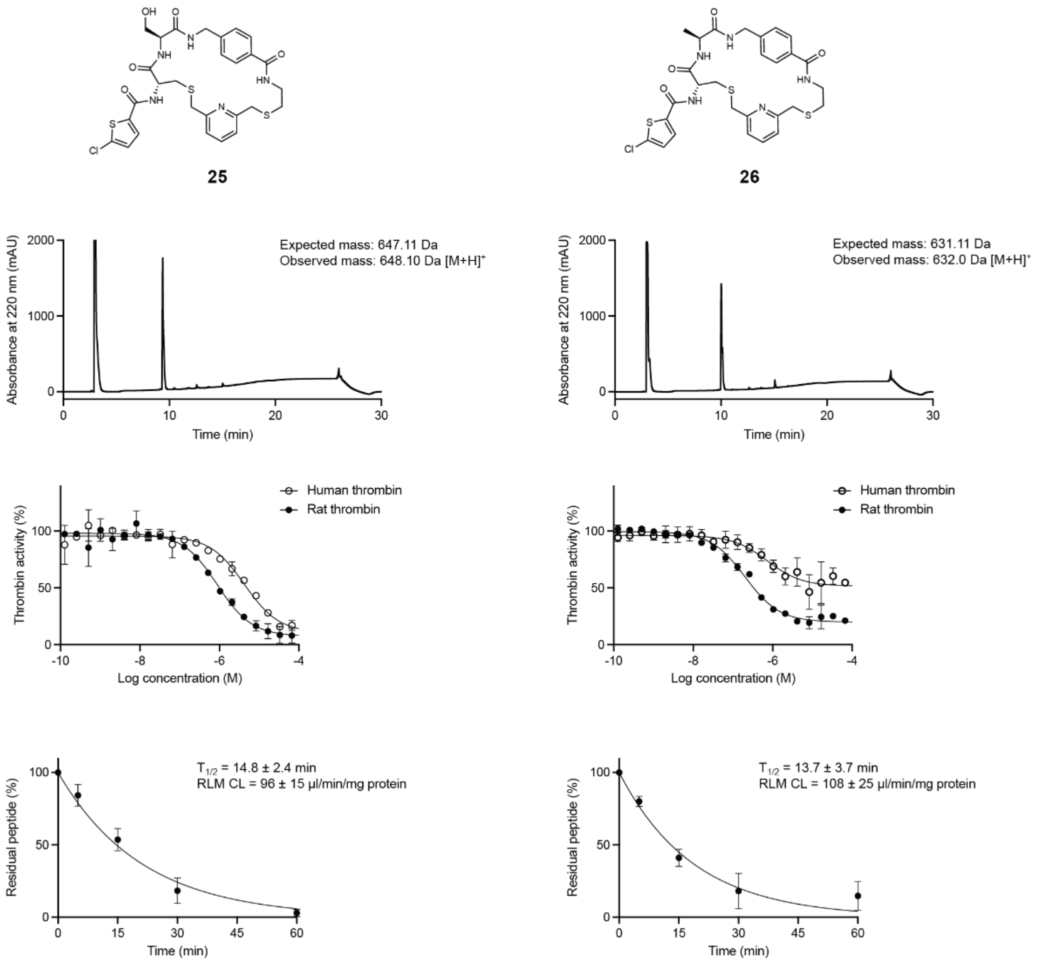

Supplementary Fig. 8 | Continued

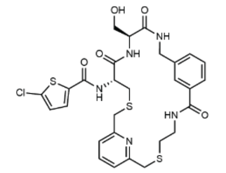

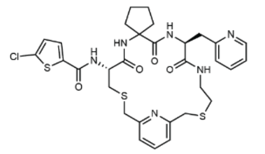

27 **28**

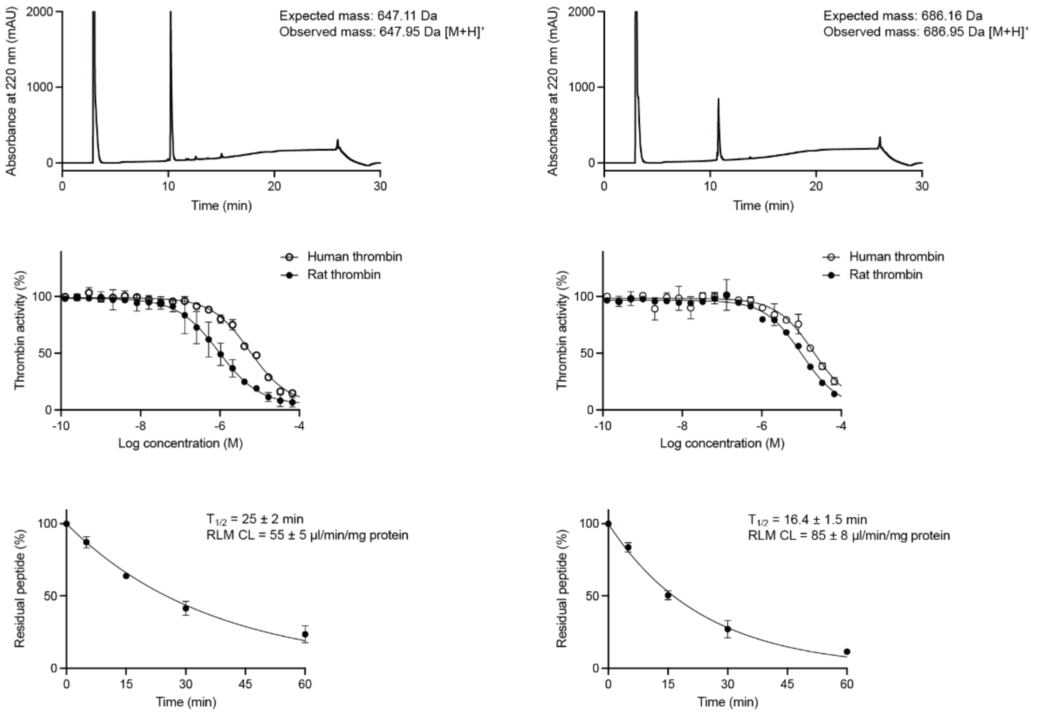

Supplementary Fig. 8 | Continued

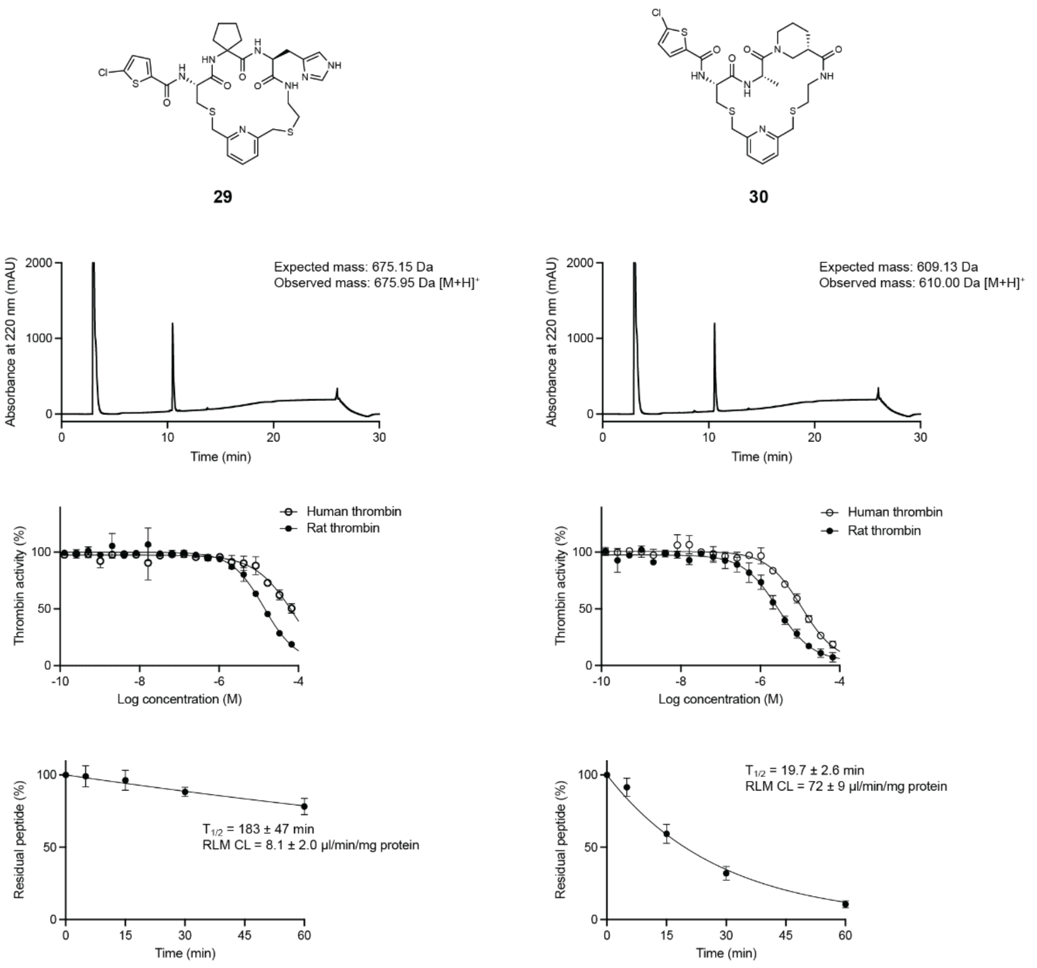

Supplementary Fig. 8 | Continued

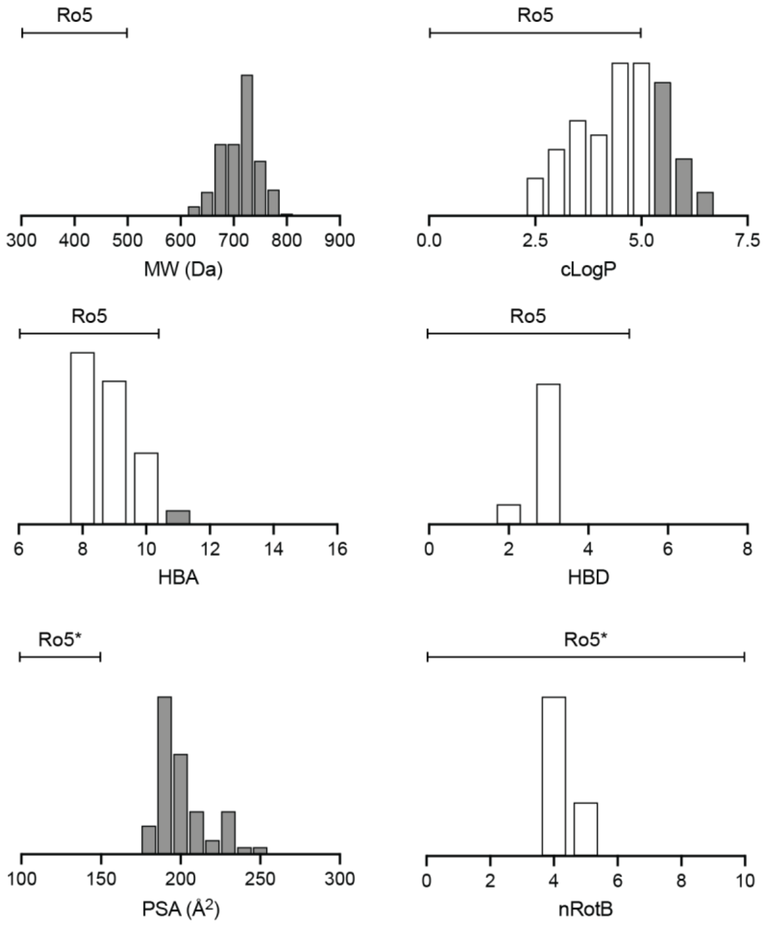

Supplementary Fig. 9 | Physico-chemical properties of cyclic peptides of sub-library based on peptide 11. DataWarrior software was used to calculate the physico-chemical properties. Cyclic peptides outside the Lipinski's rule of 5 (Ro5) space are shown in gray. Ro5* indicates a range for PSA abd bRotB that is defined by Veber *et al*. MW represents the molecular weight, cLogP the calculated lipophilicity (octanol/water partition coefficient), HBA the number of hydrogen bond acceptors, HBD the number of hydrogen bond donors, PSA the polar surface area, and nRotB the number of rotatable bonds.

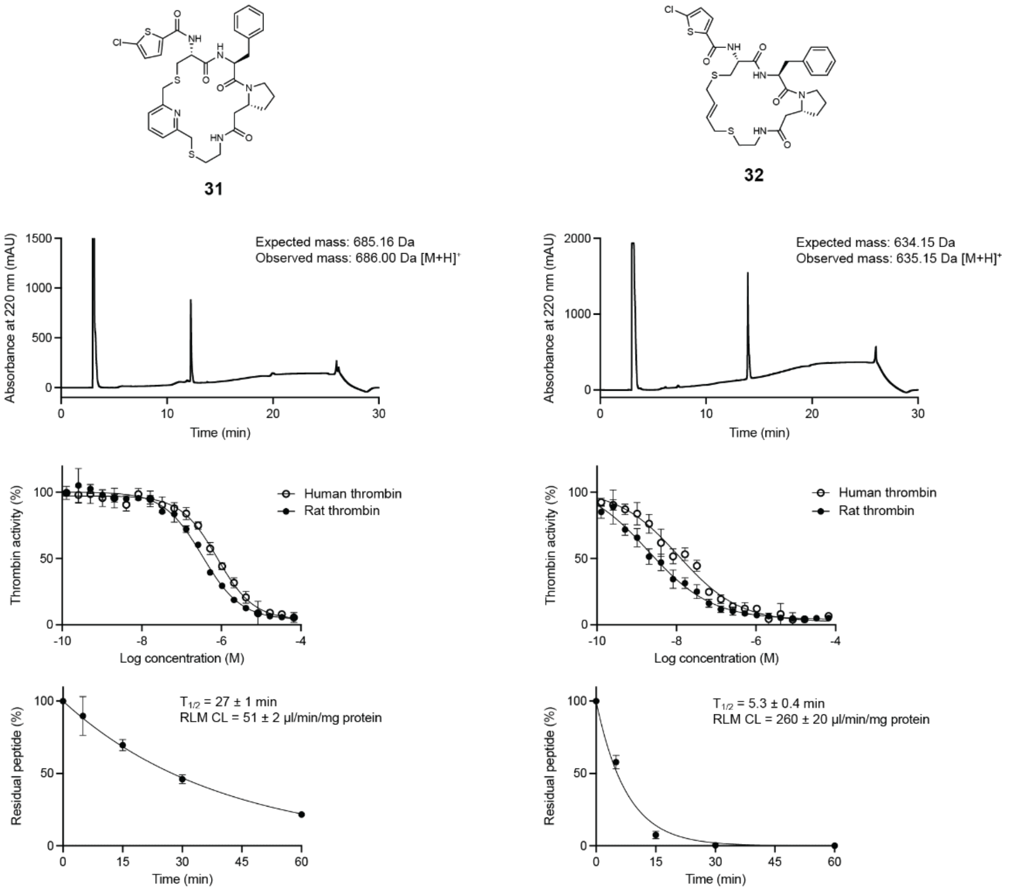

Supplementary Fig. 10 | Characterization of thrombin inhibitors from the sub-library. Chemical structures, RP-HPLC chromatograms, IC_{50} profiles, and microsomal stability curves in rat liver microsomes (top to bottom) for cyclic peptides **31-37** are shown. Mean values and SDs of the thrombin inhibition and metabolic stability were measured in three independent measurements.

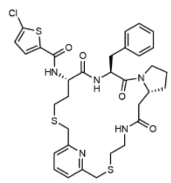

33

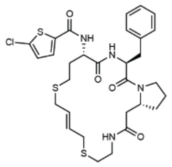

34

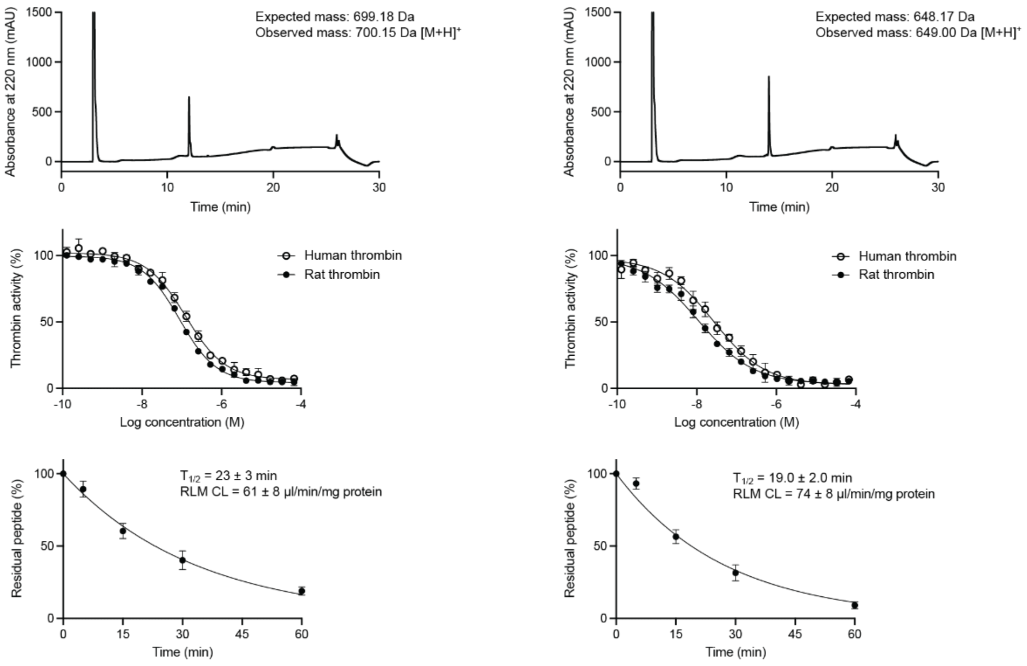

Supplementary Fig. 10 | Continued.

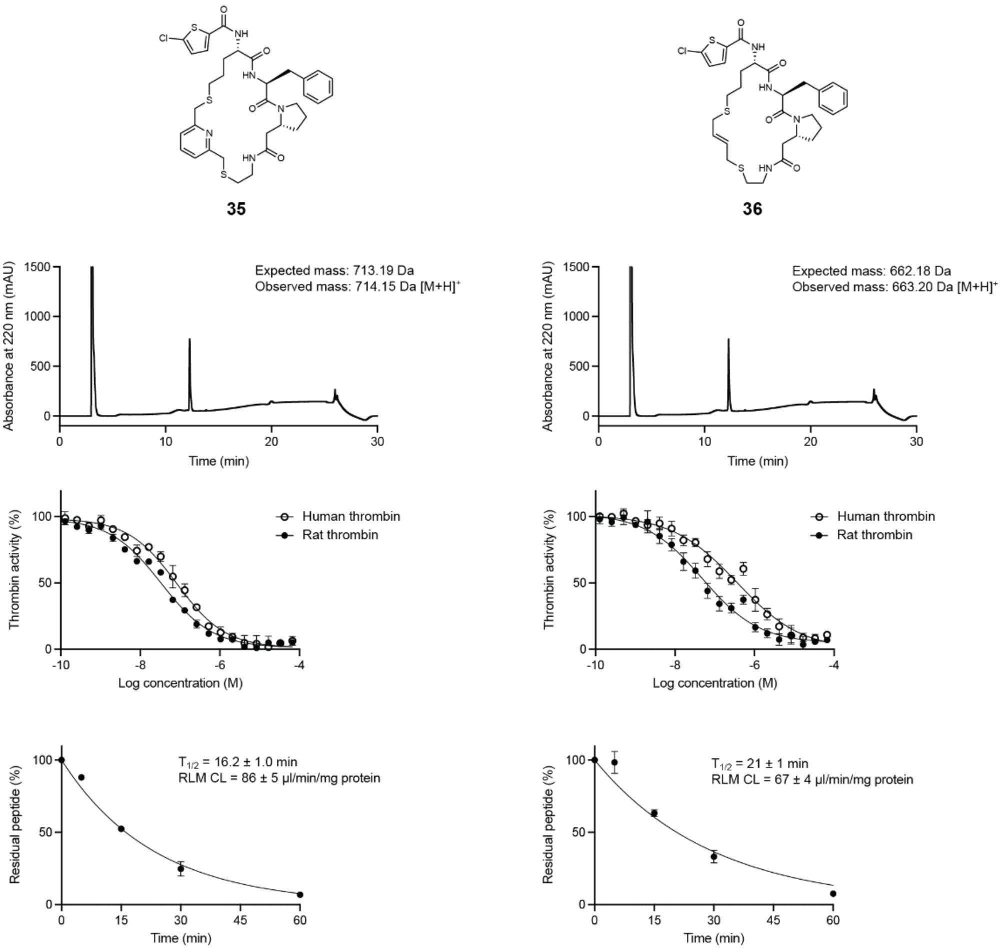

Supplementary Fig. 10 | Continued.

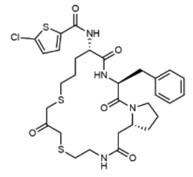

37

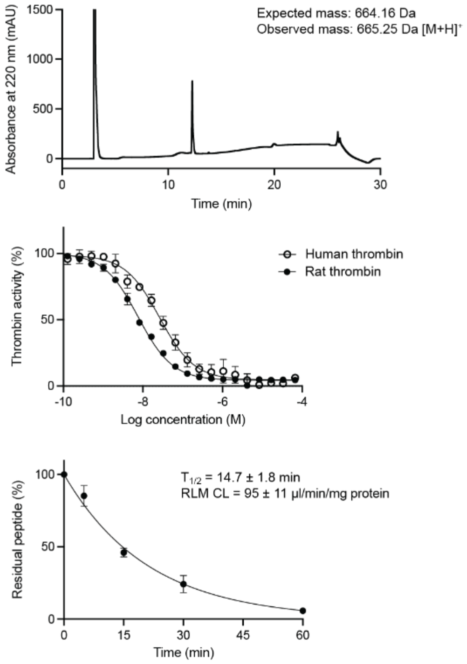

Supplementary Fig. 10 | Continued.

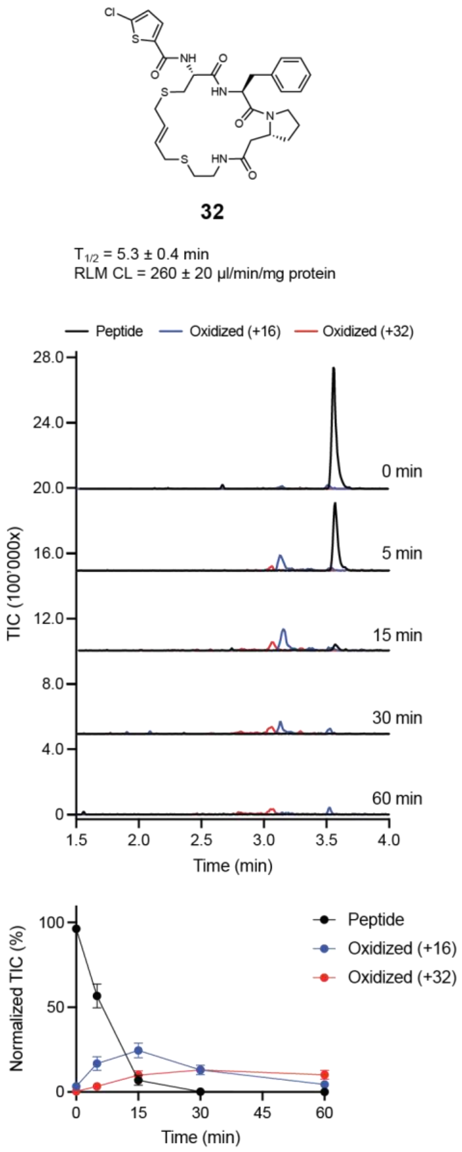

Supplementary Fig. 11 │Oxidation of peptide 32 by liver microsome treatment. Chemical structure of cyclic peptide 32 is shown on top. Half-live ($T_{1/2}$) and clearance (RLM CL) was measured in rat liver microsome incubations and residual peptide analyzed at different time points. Stacked TIC chromatograms acquired for a single replicate at the different time points and curves with normalized TIC values are shown. Emerging metabolized products are indicated by the blue and red colored profiles and curves. Mean values and SDs from three independent measurements are shown for the metabolic stability.

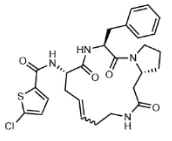

38

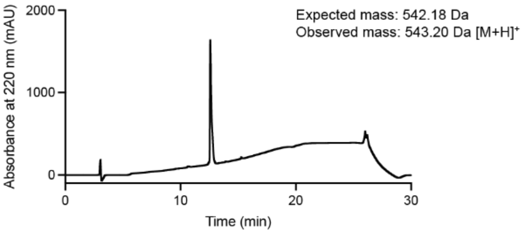

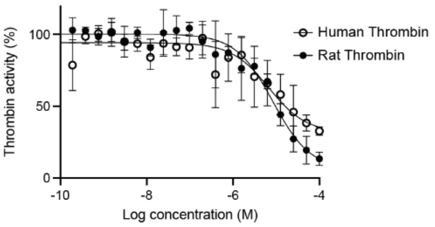

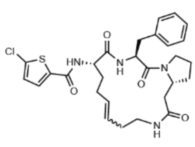

39

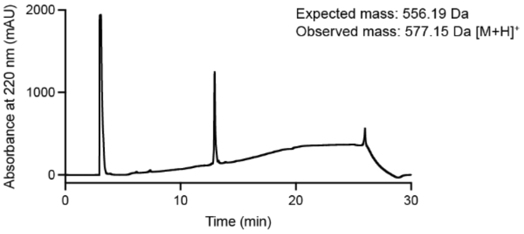

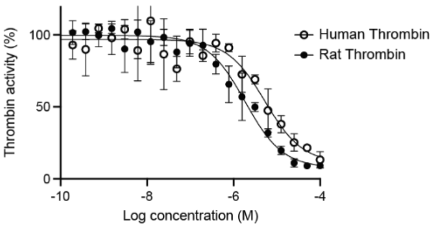

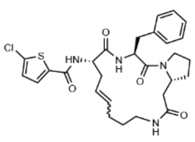

40

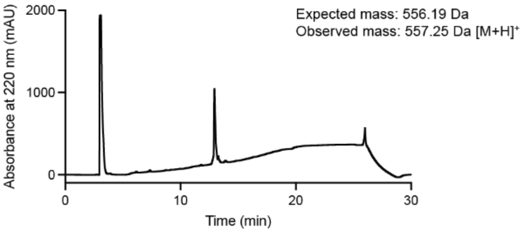

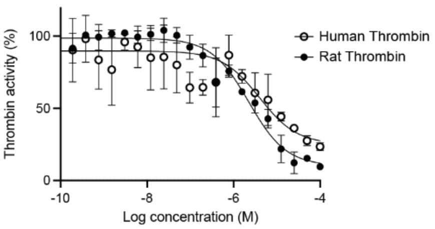

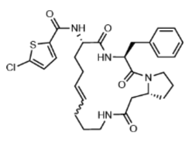

41

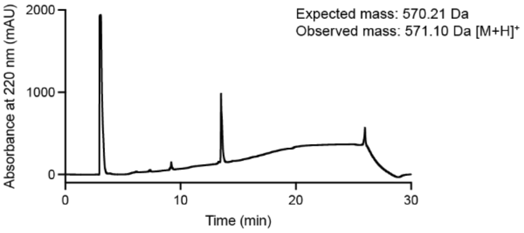

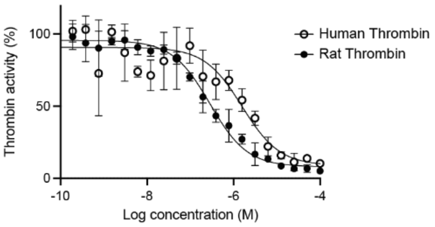

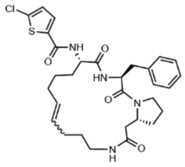

42

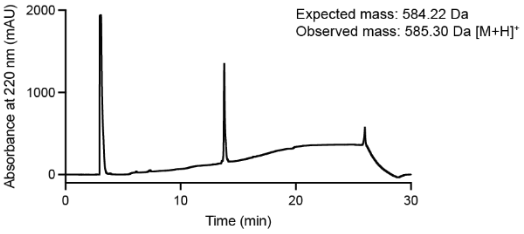

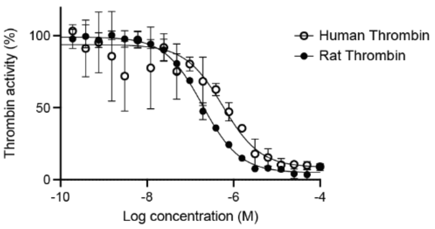

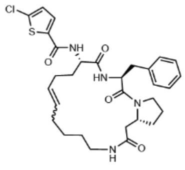

43

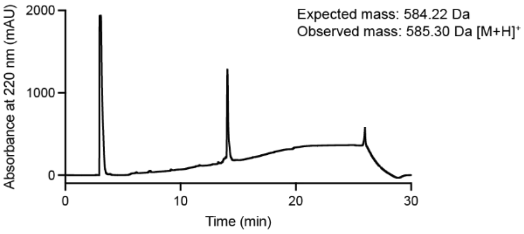

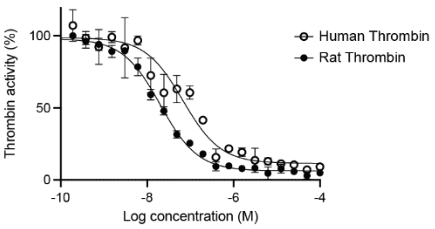

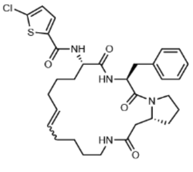

44

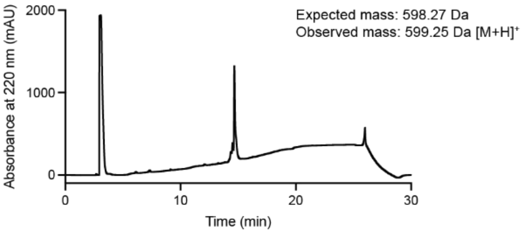

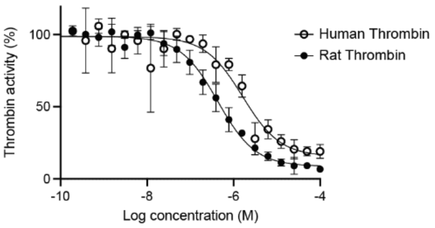

Supplementary Fig. 12 | Characterization of ring-closing metathesis (RCM) thrombin inhibitors. Chemical structures, RP-HPLC chromatograms, and IC_{50} profiles (left to right) of cyclic peptide scaffolds **38-44**. Mean values and SDs from three independent measurements of the thrombin inhibition are shown.

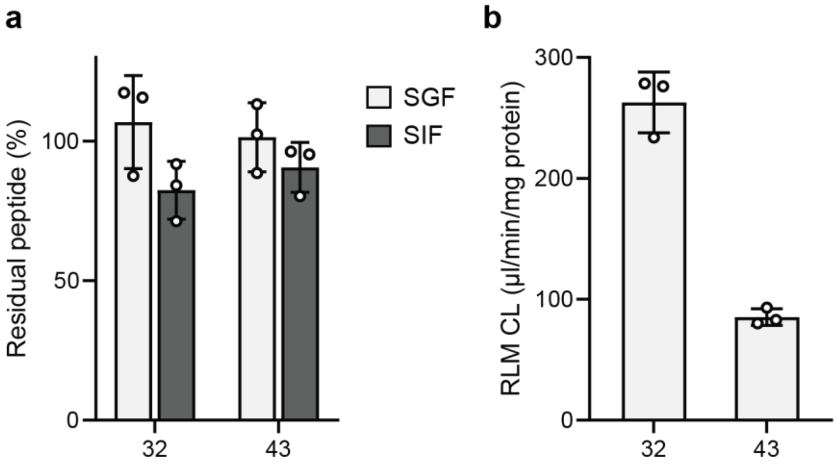

Supplementary Fig. 13 │Stability of cyclic peptide 43. a, Proteolytic stability. The peptide **43** was incubated in simulated gastric fluid (SGF) or simulated intestinal fluid (SIF) for 8 h at 37°C and residual peptide analyzed by LC-MS. Mean values and SDs from three independent measurements are shown. **b,** Comparison of metabolic clearance of dithioether peptide **32** and RCM peptide **43**. The peptides were incubated with rat liver microsomes and samples analyzed by LC-MS at different time points. Clearance in rat liver microsomes (RLM CL) was calculated based on the peptide half-live and the amount of protein in the incubation. Mean values and SDs from three independent measurements are shown.

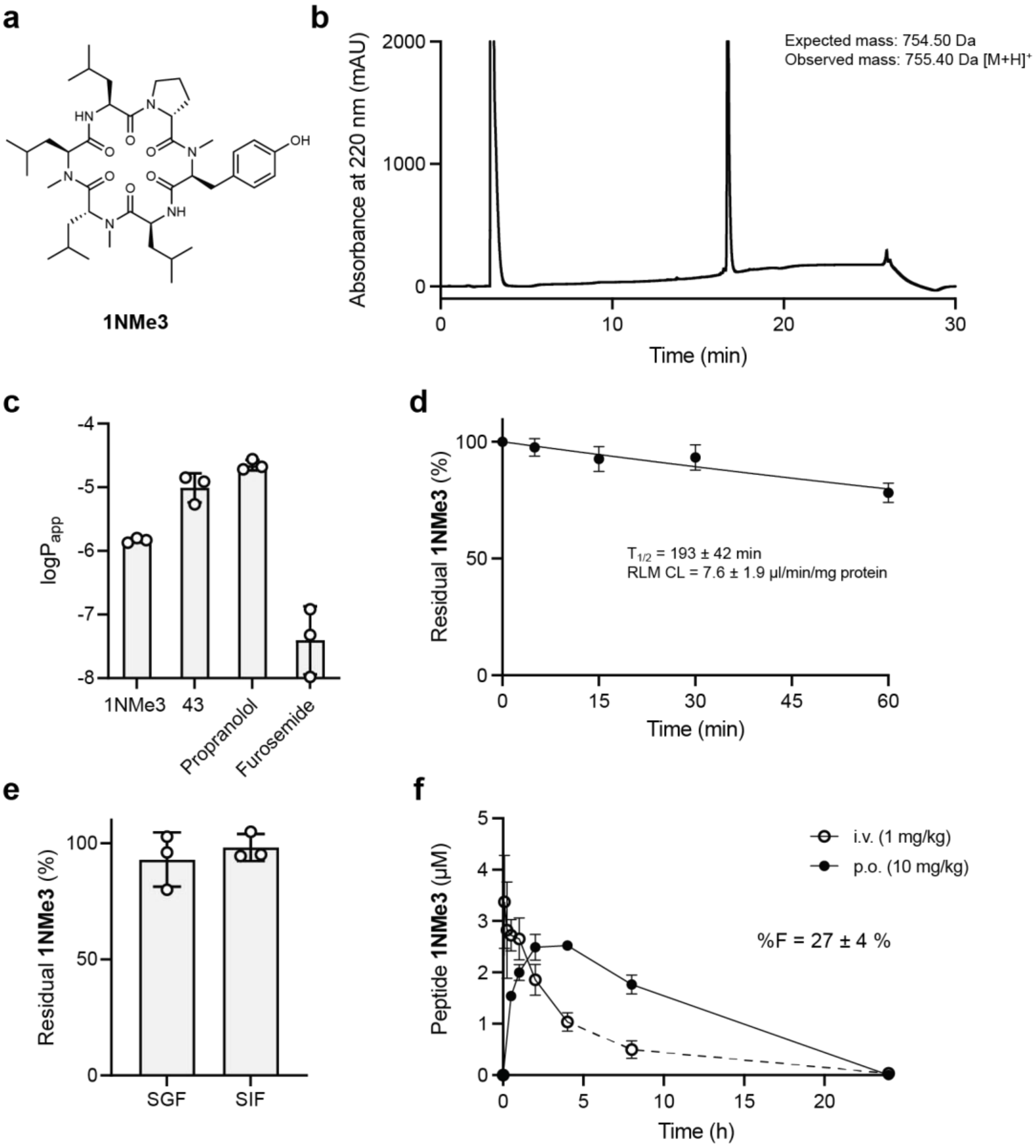

Supplementary Fig. 14 | Characterization and oral availability of the cyclic peptide 1NMe3. a, Structure of 1NMe3**. b,** RP-HPLC chromatogram. **c,** PAMPA permeability comparison of **1NMe3** and **43**. Pe value threshold of 15 nm/s (logPe = -5.82) was used to distinguish high and low permeability compounds. Mean values and SDs from three independent measurements are shown. **d,** Metabolic stability of **1NMe3** in rat liver microsomes. Mean values and SDs from three independent measurements are shown. **e,** Proteolytic stability of **1NMe3** in simulated gastric fluid (SGF) and simulated intestinal fluid (SIF). Mean values and SDs from three independent measurements are shown. **f,** Oral bioavailability (%F) of **1NMe3**. Plasma sample analysis was done by LC-MS after both oral (p.o.) and intravenous (i.v.) administration and the AUC of the resulting curves used for calculation of the oral availability. Mean values and SDs from three independent

measurements are shown. Since the plasma concentration after i.v. administration of **1NMe3** did not return to the baseline after the final 4 h time point of plasma sample collection, theoretical 8 h and 24 h time points were calculated (dashed line) using the equation for a one phase decay in Graphpad Prism 9 and the measured half-live of 1.83 h.

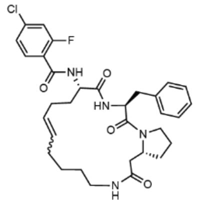

45

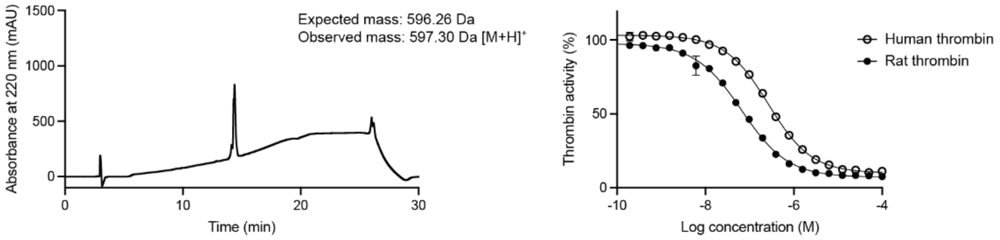

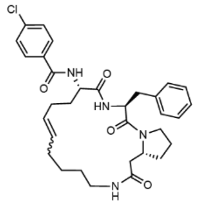

46

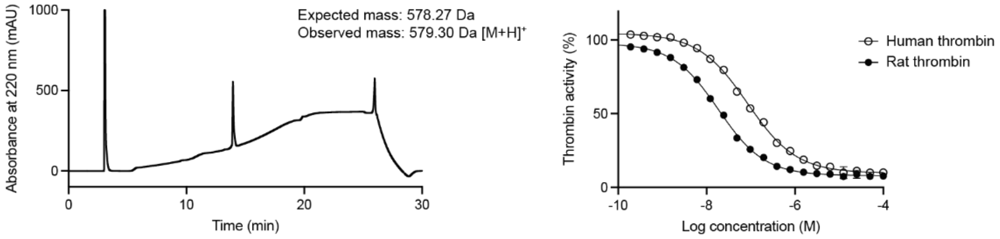

Supplementary Fig. 15 ⎸Characterization of RCM thrombin inhibitors with diverse *N*-acylation. Chemical structures, RP-HPLC chromatograms, and IC_{50} profiles (left to right) of cyclic peptide scaffolds **45** and **46**. Mean values and SDs from three independent measurements for the thrombin inhibition are shown.

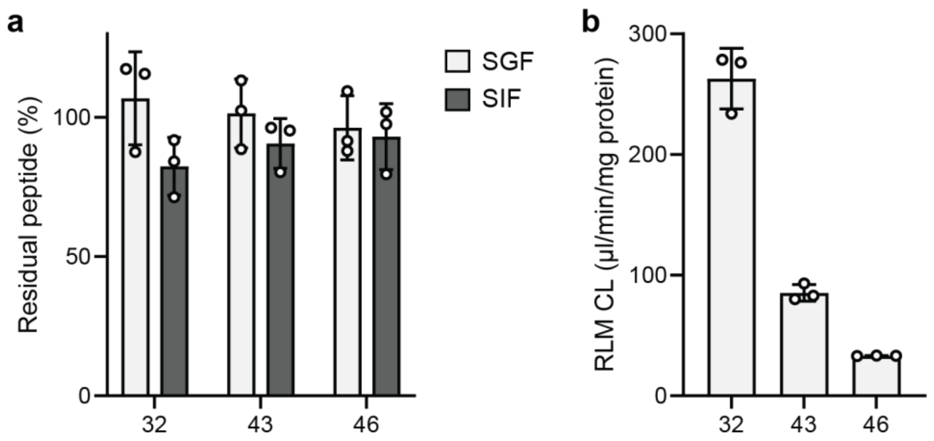

Supplementary Fig. 16 │Stability of RCM thrombin inhibitors with diverse *N*-acylation. a, Proteolytic stability of cyclic peptide **46** after 8 h incubation at 37°C in SGF and SIF. Mean values and SDs from three independent measurements are shown. **b,** Comparison of the metabolic stability of cyclic peptide scaffolds **32**, **43**, and **46**. Clearances were measured in rat liver microsomes. Mean values and SDs from three independent measurements are shown.

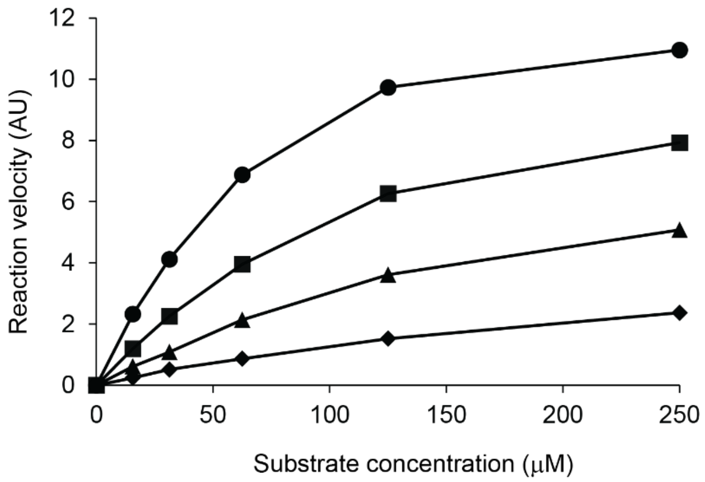

Supplementary Fig. 17 | Binding mode of peptide 46 assessed by measuring the Michaelis-Menten kinetics. The reaction velocity (y-axis) is indicated as arbitrary units that are proportional to the fluorescence intensity and thus the amount of substrate cleaved. The reactions were performed without inhibitor (circle) and with 50 nM (square), 150 nM (triangle) and 450 nM (diamond) of peptide **46**. At increasing inhibitor concentration, the Michaelis-Menten constant K_m became larger, while the maximal velocity V_{max} remained constant, pointing to a competitive inhibition and indicating that the inhibitor binds to the enzyme's active site. The activity of thrombin was measured once for each substrate and inhibitor concentration combination.

Supplementary Tables

Supplementary Table 1 │Cyclic peptide high-throughput screen.

Category	Parameter	Description
Assay	Type of assay	In vitro
	Target	Human α thrombin
	Primary measurement	Proteolytic cleavage of fluorogenic substrate
	Key reagents	Z-Gly-Gly-Arg-AMC (Bachem)
	Assay protocol	Described in Materials section "*Thrombin library synthesis and screening*"
	Additional comments	-
Library	Library size	8,448
	Library composition	Cyclic peptides
	Source	Combinatorial synthesis. Described in Methods section "*Thrombin library synthesis and screening*"
	Additional comments	-
Screen	Format	384 well microtiter plates (Greiner, 781076)
	Concentration(s) tested	10 μM
	Plate controls	20 wells DMSO, 4 wells human α thrombin inhibitor R-11-25-2 (DOI: 10.1039/d0sc01944e)
	Reagent/ compound dispensing system	Echo 650 (Beckman Coulter), CERTUS FLEX (Gyger)
	Detection instrument and software	Tecan Infinite M200 Pro microplate reader
	Assay validation/QC	Z' score: 0.86
	Correction factors	-
	Normalization	Percent fluorescent gain over time as compared to DMSO
	Additional comments	-
Post-HTS analysis	Hit criteria	Higher than 50% inhibition as compared to DMSO
	Hit rate	0.9%
	Additional assay(s)	Resynthesis and retesting of 20 best hits in original assay
	Confirmation of hit purity and structure	Compounds were resynthesized and verified analytically
	Additional comments	-

Supplementary Table 2 │*In vitro* properties of peptides and reference compounds. Mean and SD values for three independent measurements are shown.

Peptide/ Compound	Metabolic stability		Proteolytic stability		PAMPA permeability	
	Half life $T_{1/2}$ (min)	RLM CL (ul/min/mg)	SIF (8 h) % remaining	SGF (8 h) % remaining	% permeability	$logP_{app}$
1	11.5 ± 1.7	123 ± 18	-	-	-	-
2	133 ± 21	10.7 ± 1.9	-	-	-	-
3	16.9 ± 2.5	84 ± 11	-	-	-	-
4	35 ± 4	41 ± 5	-	-	-	-
5	101 ± 15	14.1 ± 2.3	-	-	-	-
6	100 ± 35	16.6 ± 7.6	-	-	-	-
7	31 ± 5	45 ± 6	-	-	-	-
8	37 ± 10	40 ± 9	-	-	-	-
9	22 ± 4	64 ± 11	-	-	-	-
10	80 ± 11	17.6 ± 2.6	-	-	-	-
11	17.3 ± 2.3	82 ± 10	14.0 ± 4.4	36 ± 7	1.23 ± 0.72	-7.15 ± 0.26
12	18.2 ± 2.0	77 ± 9	96 ± 6	99 ± 5	0.35 ± 0.03	-7.64 ± 0.03
13	9.5 ± 1.8	151 ± 29	96 ± 11	104 ± 8	2.0 ± 1.7	-6.97 ± 0.31
14	12.7 ± 3.6	117 ± 28	87 ± 11	95 ± 7	1.27 ± 0.67	-7.11 ± 0.18
15	17.0 ± 1.0	82 ± 5	88 ± 18	99 ± 2	20 ± 10	-5.87 ± 0.24
16	24 ± 2	58 ± 5	6.3 ± 1.9	99 ± 7	8.6 ± 3.0	-6.24 ± 0.12
17	12.1 ± 2.0	119 ± 22	92 ± 9	98 ± 2	0.86 ± 0.09	-7.24 ± 0.04
18	13.5 ± 1.2	103 ± 10	99 ± 9	100 ± 2	2.73 ± 1.02	-6.76 ± 0.14
19	53 ± 38	41 ± 23	62 ± 15	95 ± 4	5.7 ± 1.3	-6.42 ± 0.08
20	53 ± 4	27 ± 2	93 ± 13	97 ± 5	0.99 ± 0.28	-7.19 ± 0.10
21	17.6 ± 4.0	82 ± 17	73 ± 14	97 ± 2	17 ± 5	-5.92 ± 0.10
22	5.4 ± 0.2	260 ± 10	96 ± 9	96 ± 2	16 ± 7	-5.98 ± 0.18
23	18.8 ± 0.7	74 ± 3	91 ± 9	99 ± 3	63 ± 5	-5.19 ± 0.05
24	18.9 ± 1.0	74 ± 4	95 ± 6	99 ± 3	2.0 ± 1.7	-7.31 ± 0.86
25	14.8 ± 2.4	96 ± 15	93 ± 9	101 ± 8	0.62 ± 0.14	-7.39 ± 0.08
26	13.7 ± 3.7	108 ± 25	88 ± 5	97 ± 2	4.3 ± 1.6	-6.56 ± 0.15
27	25 ± 2	55 ± 5	92 ± 9	99 ± 2	2.5 ± 1.2	-6.82 ± 0.18
28	16.4 ± 1.4	85 ± 8	94 ± 12	95 ± 4	30 ± 7	-5.64 ± 0.10
29	183 ± 47	8.1 ± 2.0	92 ± 16	101 ± 8	0.16 ± 0.08	-8.03 ± 0.21
30	19.7 ± 2.5	72 ± 9	98 ± 11	90 ± 10	27 ± 1	-5.67 ± 0.01
31	27 ± 1	51 ± 2	76 ± 8	92 ± 6	47 ± 3	-5.21 ± 0.03
32	5.3 ± 0.4	260 ± 20	82 ± 10	107 ± 17	47 ± 12	-5.21 ± 0.13
33	23 ± 3	61 ± 8	69 ± 6	95 ± 6	45 ± 10	-5.23 ± 0.11
34	19.0 ± 2.0	74 ± 8	63 ± 6	99 ± 8	37 ± 1	-5.34 ± 0.02
35	16.2 ± 1.0	86 ± 5	31 ± 3	99 ± 3	21 ± 8	-5.65 ± 0.15
36	21 ± 1	67 ± 4	42 ± 5	87 ± 6	26 ± 9	-5.54 ± 0.16
37	14.7 ± 1.7	95 ± 11	83 ± 14	106 ± 12	31 ± 10	-5.46 ± 0.16
38	-	-	-	-	32 ± 6	-5.46 ± 0.08
39	-	-	-	-	70 ± 4	-4.94 ± 0.04
40	-	-	-	-	42 ± 11	-5.31 ± 0.14
41	-	-	-	-	64 ± 11	-5.03 ± 0.11
42	-	-	-	-	70 ± 22	-4.95 ± 0.23
43	16.3 ± 1.0	85 ± 6	91 ± 9	101 ± 12	66 ± 19	-5.01 ± 0.18
44	-	-	-	-	48 ± 19	-5.25 ± 0.22
45	-	-	-	-	66 ± 14	-4.97 ± 0.14
46	33 ± 1	43 ± 1	93 ± 12	96 ± 12	55 ± 8	-5.11 ± 0.08
1NMe3	193 ± 42	7.6 ± 1.9	98 ± 6	93 ± 12	22 ± 8	-5.79 ± 0.13
Verapamil	22 ± 4	66 ± 4	-	-	-	-
Diltiazem	13.4 ± 1.1	104 ± 8	-	-	-	-
Testosterone	2.2 ± 0.1	620 ± 10	-	-	88.2 ± 1.1	-4.85 ± 0.02
Warfarin	26000 ± 18000	0.91 ± 1.23	-	-	50 ± 5	-5.34 ± 0.05
Propranolol	-	-	-	-	71 ± 9	-4.91 ± 0.09
Furosemide	-	-	-	-	0.90 ± 0.84	-7.40 ± 0.44

Supplementary Notes

Supplementary Note 1 ︱NMR analysis of peptide 46. Two regioisomers are present in a 1:2 ratio (E:Z) stemming from isomerism around the alkene. **a,** Chemical structure with atoms labeled. **b,** ^{1}H NMR (800 MHz, $CDCl_3$) of peptide **46**. *Impurity. **c,** ^{13}C NMR (151 MHz, $CDCl_3$) of peptide **46**. **d,** NMR peak assignments. *overlap with solvent peak. **e,** Zoomed peaks highlighting the ratio between the two isomers. **f,** Zoomed region showing the coupling pattern around the alkene-protons and identifying the E-alkene as the minor isomer.

a

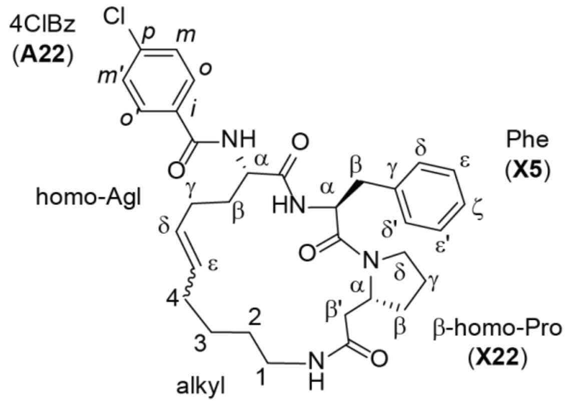

b

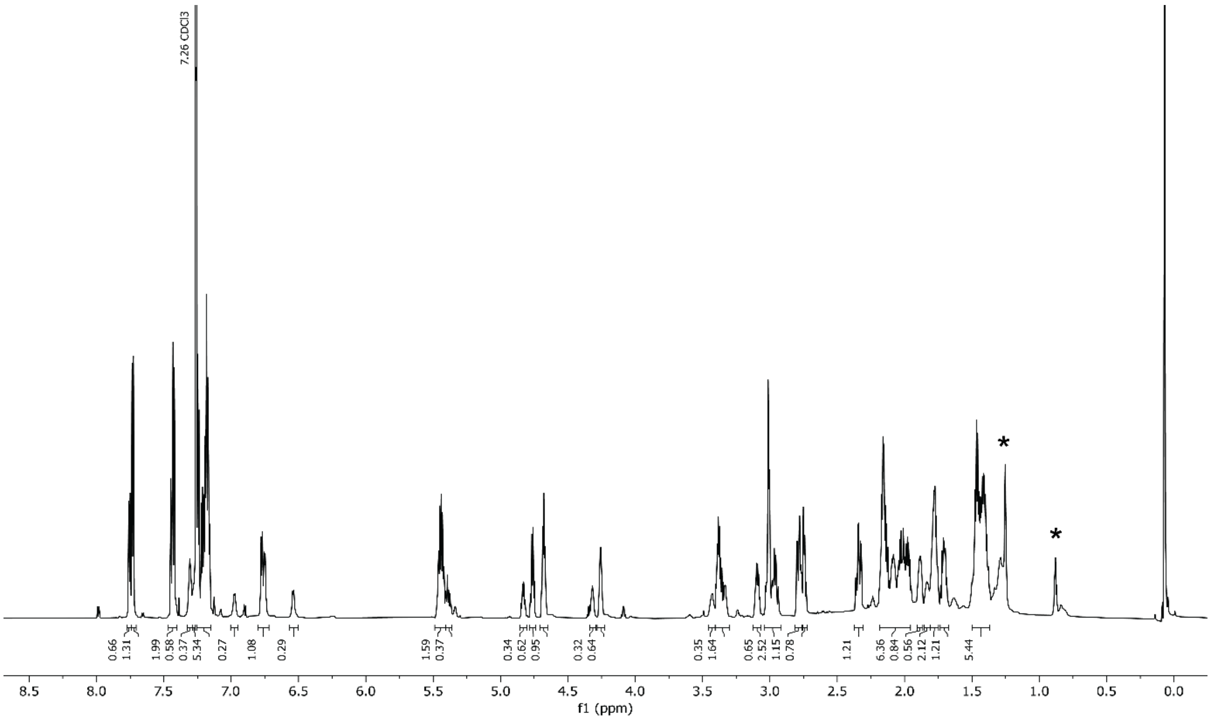

c

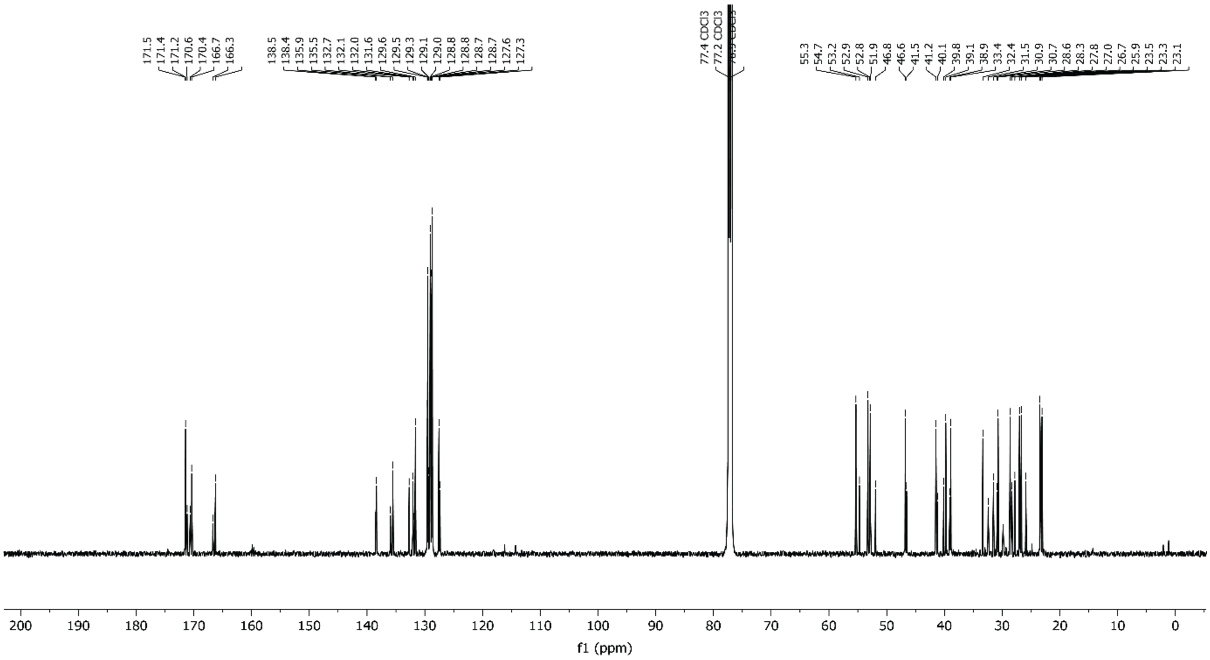

d

Residue	Position	Major regioisomer (*Z*)		Minor regioisomer (*E*)	
		^{1}H (m, *J* in Hz)	^{13}C	^{1}H (m, *J* in Hz)	^{13}C
D–β-homo-Pro (**X22**)	α	4.28–4.23 (m)	55.3	4.33–4.29 (m)	54.7
	β	a. 1.91–1.85 (m)<br>b. 1.73–1.67 (m)	30.7	a. 1.85–1.81 (m)<br>b. 1.73–1.67 (m)	30.9
	β'	a. 2.83–2.75 (m)<br>b. 2.38–2.30 (m)	41.5	a. 2.83–2.75 (m)<br>b. 2.38–2.30 (m)	41.2
	γ	a. 1.81–1.75 (m)<br>b. 1.52–1.34 (m)	23.5	a. 1.81–1.75 (m)<br>b. 1.52–1.34 (m)	23.3
	δ	a. 3.41–3.31 (m)<br>b. 2.75–2.71 (m)	46.8	a. 3.41–3.31 (m)<br>b. 2.75–2.71 (m)	46.6
	CO	–	171.4	–	171.5–171.4
Phe (**X5**)	α	4.76<br>(q, 7.3)	53.2	4.83<br>(q, 7.8)	52.9
	β	3.04–2.92 (m)	38.9	3.04–2.92 (m)	39.1
	γ	–	135.5	–	135.9
	δ / δ'	7.26–7.16 (m)	129.5	7.26–7.16 (m)	129.6
	ε / ε'	7.26–7.16 (m)	128.8–128.7	7.26–7.16 (m)	128.8–128.7
	ζ	7.26–7.16 (m)	127.6	7.26–7.16 (m)	127.3
	NH	6.75 (d, 7.1)	–	6.97 (d, 7.6)	–
	CO	–	170.4	–	170.6
homo-Agl	α	4.71–4.64 (m)	52.8	4.71–4.64 (m)	51.9
	β	a. 2.18–1.95 (m)<br>b. 1.81–1.75 (m)	33.4	a. 2.18–1.95 (m)<br>b. 1.81–1.75 (m)	32.4
	γ	2.18–1.95 (m)	23.1	2.18–1.95 (m)	25.9

	δ	5.48–5.41 (m)	128.8–128.7	5.48–5.41 (m)	129.3
	ε	5.48–5.41 (m)	131.6	5.38<br>(dt, 15.4, 6.6)	132.7
	NH	6.77 (d, 7.7)	–	6.54 (d, 8.1)	–
	CO	–	171.5	–	171.2
alkyl	1	a. 3.41–3.31 (m)<br>b. 3.14–3.07 (m)	39.8	a. 3.46–3.41 (m)<br>b. 3.04–2.92 (m)	40.1
	2	1.52–1.34 (m)	28.6	1.52–1.34 (m)	28.3
	3	1.52–1.34 (m)	26.7	1.52–1.34 (m)	27.8
	4	2.18–1.95 (m)	27.0	2.18–1.95 (m)	31.5
	NH	7.31<br>(t, 5.8)	–	7.28–7.25*<br>(t, 5.8, 1H)	–
4ClBz (**A22**)	*i*	–	132.1	–	132.0
	o / *o'*	7.43<br>(d, 8.2)	129.0	7.44<br>(d, 8.5)	129.1
	m / *m'*	7.73<br>(d, 8.1)	128.8–128.7	7.76<br>(d, 8.0)	128.8–128.7
	p	–	138.4	–	138.5
	CO	–	166.3	–	166.7

e

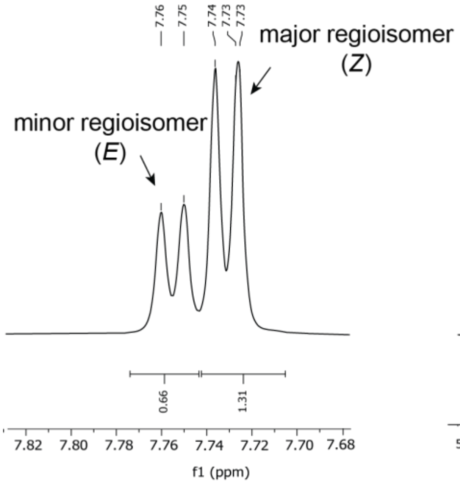

f

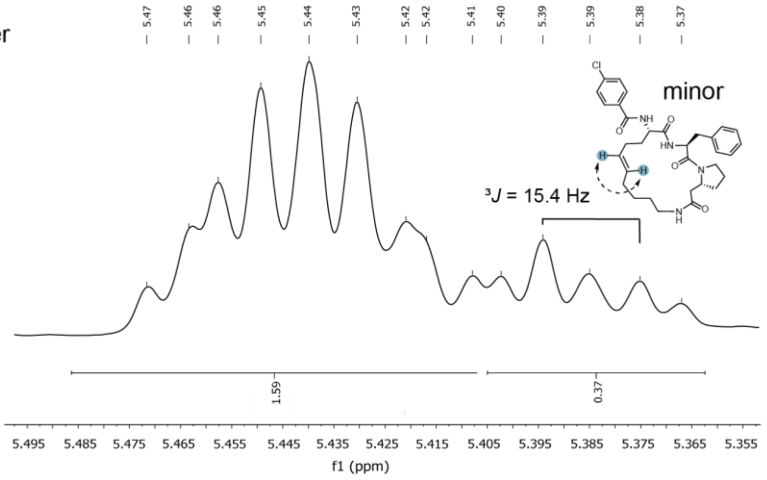

Supplementary Note 2 | High-resolution mass spectra of peptides 11, 32, 43 and 46.

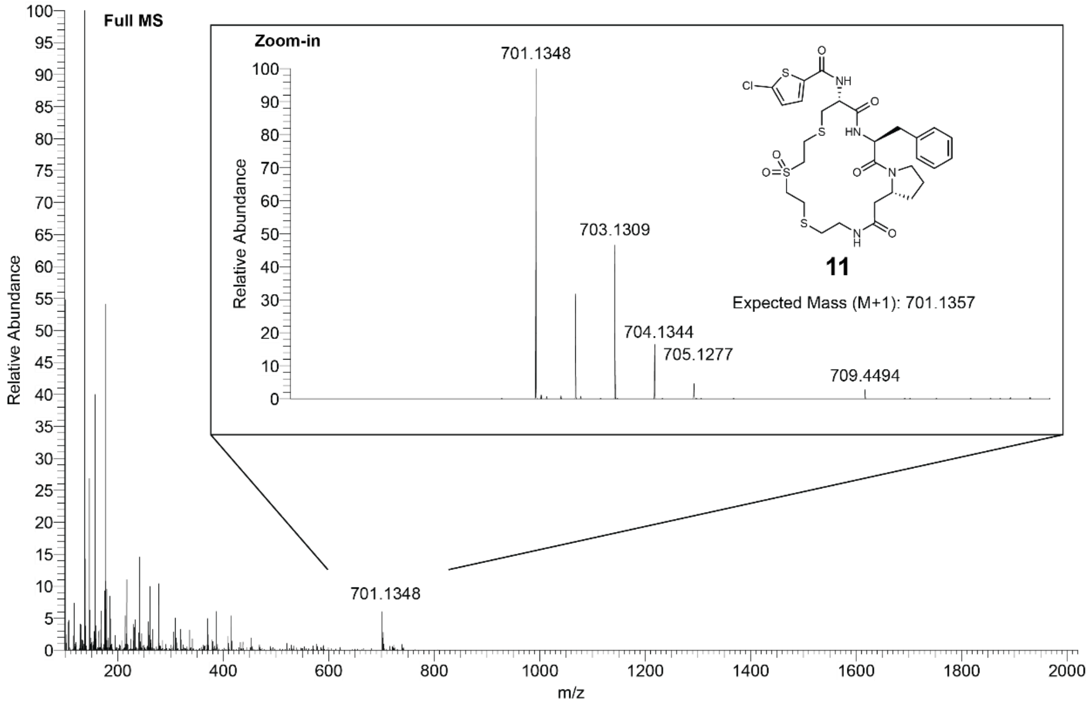

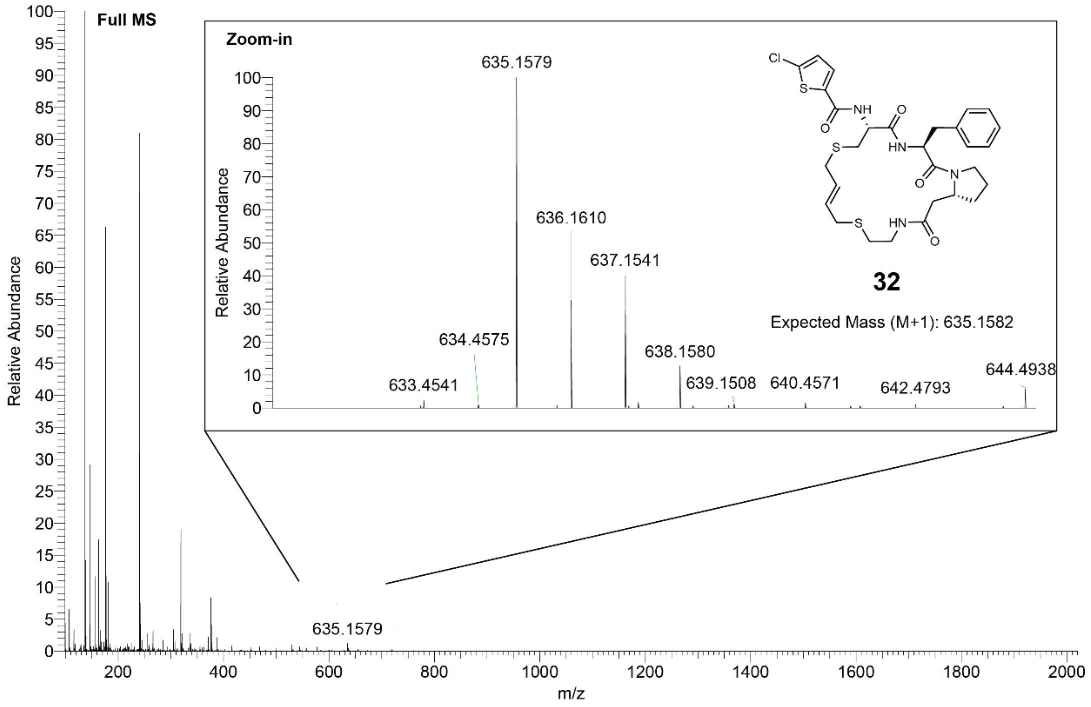

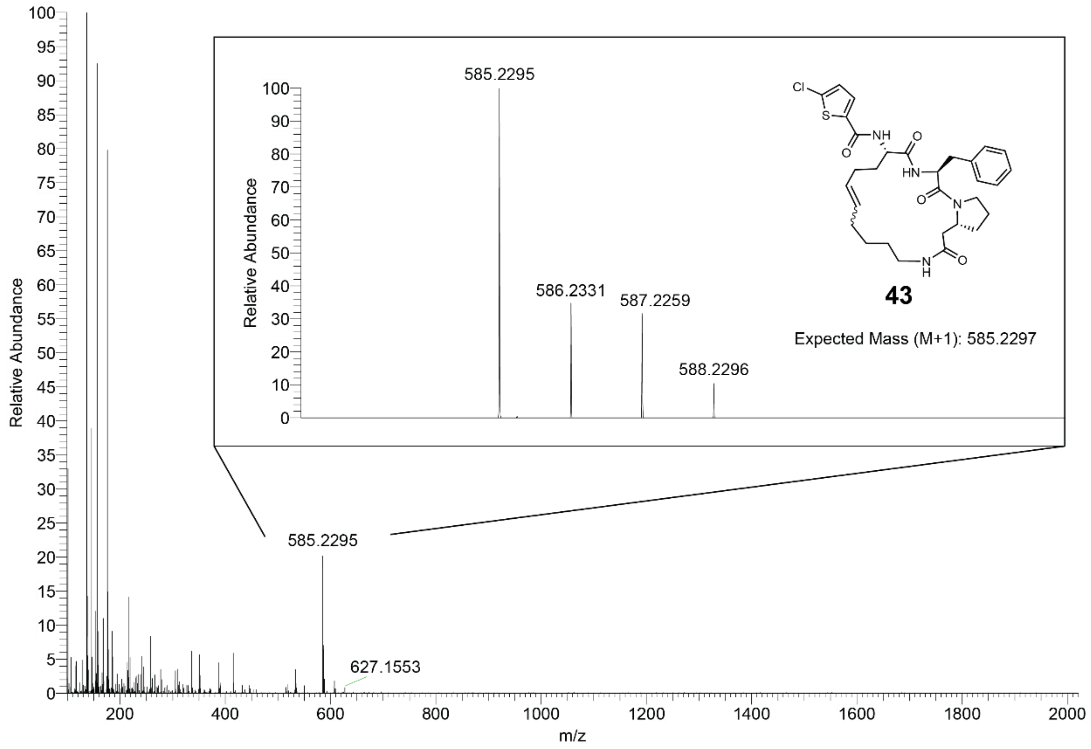
585.2295
586.2331
587.2259
588.2296
43
Expected Mass (M+1): 585.2297
585.2295
627.1553
Relative Abundance
m/z

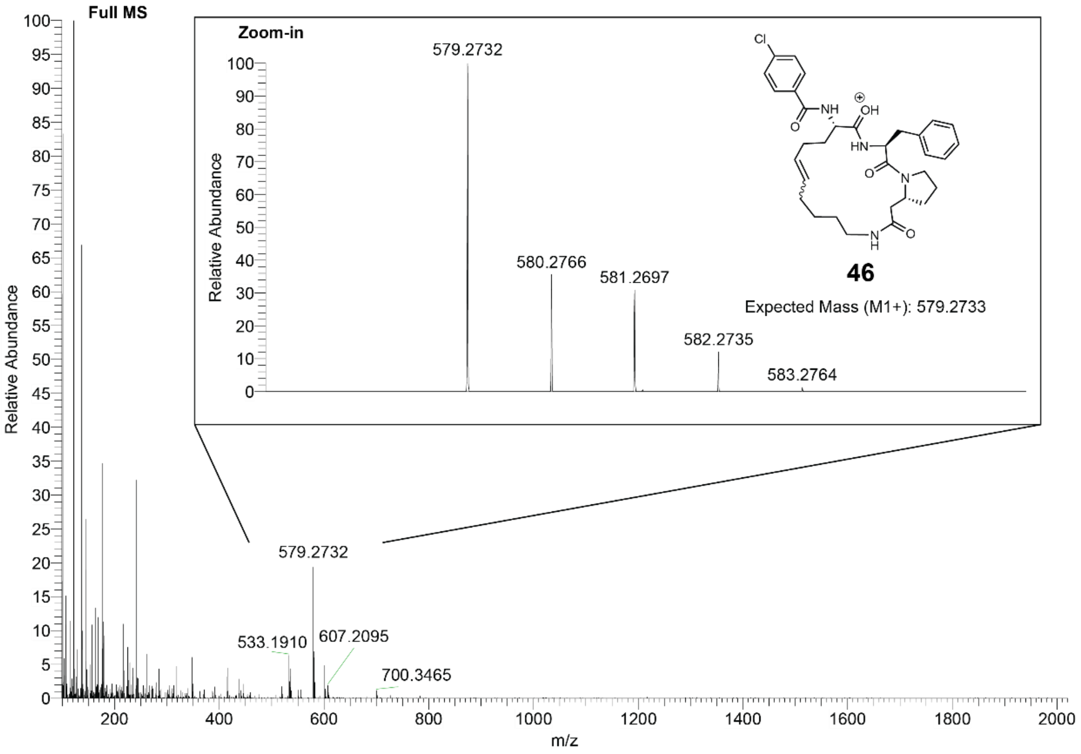
Full MS
Zoom-in
579.2732
580.2766
581.2697
582.2735
583.2764
46
Expected Mass (M1+): 579.2733
579.2732
533.1910
607.2095
700.3465
Relative Abundance
m/z

References

1. Lipinski, C. A., Lombardo, F., Dominy, B. W. & Feeney, P. J. Experimental and computational approaches to estimate solubility and permeability in drug discovery and development settings. *Adv Drug Deliv Rev* **46**, 3–26 (2001).

2. Veber, D. F. *et al.* Molecular Properties That Influence the Oral Bioavailability of Drug Candidates. *J Med Chem* **45**, 2615–2623 (2002).
