# Supplementary material for: De novo development of small cyclic peptides that are orally bioavailable
Source: Nat Chem Biol. 2023 Dec 28;20(5):624–33. doi: 10.1038/s41589-023-01496-y (PMC11062899; doi:10.1038/s41589-023-01496-y)
Supplement: Supplementary file 2 — Reporting Summary [file 41589_2023_1496_MOESM2_ESM.pdf]

nature portfolio

Corresponding author(s): Christian Heinis

Last updated by author(s): Oct 18, 2023

Reporting Summary

Nature Portfolio wishes to improve the reproducibility of the work that we publish. This form provides structure for consistency and transparency in reporting. For further information on Nature Portfolio policies, see our Editorial Policies and the Editorial Policy Checklist.

Statistics

For all statistical analyses, confirm that the following items are present in the figure legend, table legend, main text, or Methods section.

n/a	Confirmed	
☐	☒	The exact sample size (*n*) for each experimental group/condition, given as a discrete number and unit of measurement
☐	☒	A statement on whether measurements were taken from distinct samples or whether the same sample was measured repeatedly
☒	☐	The statistical test(s) used AND whether they are one- or two-sided<br>*Only common tests should be described solely by name; describe more complex techniques in the Methods section.*
☒	☐	A description of all covariates tested
☒	☐	A description of any assumptions or corrections, such as tests of normality and adjustment for multiple comparisons
☐	☒	A full description of the statistical parameters including central tendency (e.g. means) or other basic estimates (e.g. regression coefficient) AND variation (e.g. standard deviation) or associated estimates of uncertainty (e.g. confidence intervals)
☒	☐	For null hypothesis testing, the test statistic (e.g. *F*, *t*, *r*) with confidence intervals, effect sizes, degrees of freedom and *P* value noted<br>*Give P values as exact values whenever suitable.*
☒	☐	For Bayesian analysis, information on the choice of priors and Markov chain Monte Carlo settings
☒	☐	For hierarchical and complex designs, identification of the appropriate level for tests and full reporting of outcomes
☒	☐	Estimates of effect sizes (e.g. Cohen's *d*, Pearson's *r*), indicating how they were calculated

Our web collection on statistics for biologists contains articles on many of the points above.

Software and code

Policy information about availability of computer code

Data collection	DataWarrior software (version 5.2.1), LC-MS Shimadzu 2020 instrument software (LabSolutions, version 5), Tecan Infinite M200 Pro instrument software (iControl, version 1.12), PHERAstar FSX software (version 5.70, Edition 8), HR-MS Exploris 240 Mass Spectrometer software Chipsoft (Advion BioScience, version 8.3.1)
Data analysis	Excel (version 2016), Graphpad Prism (version 9), LC-MS Shimadzu 2020 instrument software (LabSolutions, version 5), Tecan Infinite M200 Pro instrument software (iControl, version 1.12), Thermo Scientific XCalibur software (version 4.1), Thermo Scientific Mass Frontier Spectral Interpretation Software (version 8)

For manuscripts utilizing custom algorithms or software that are central to the research but not yet described in published literature, software must be made available to editors and reviewers. We strongly encourage code deposition in a community repository (e.g. GitHub). See the Nature Portfolio guidelines for submitting code & software for further information.

Data

Policy information about availability of data

All manuscripts must include a data availability statement. This statement should provide the following information, where applicable:

- Accession codes, unique identifiers, or web links for publicly available datasets
- A description of any restrictions on data availability
- For clinical datasets or third party data, please ensure that the statement adheres to our policy

Two supplementary tables, 17 supplementary figures and 2 supplementary notes are provided in the Supplementary Information. Raw data shown in graphics in Figures 1—6 and Extended Data Figures 1—3 are provided as Source data files.

Human research participants

Policy information about studies involving human research participants and Sex and Gender in Research.

Reporting on sex and gender	n/a
Population characteristics	n/a
Recruitment	n/a
Ethics oversight	n/a

Note that full information on the approval of the study protocol must also be provided in the manuscript.

Field-specific reporting

Please select the one below that is the best fit for your research. If you are not sure, read the appropriate sections before making your selection.

☒ Life sciences ☐ Behavioural & social sciences ☐ Ecological, evolutionary & environmental sciences

For a reference copy of the document with all sections, see nature.com/documents/nr-reporting-summary-flat.pdf

Life sciences study design

All studies must disclose on these points even when the disclosure is negative.

Sample size	In vitro characterization: parameters such as inhibition activity, proteolytic stability, membrane permeability, and metabolic stability were measured in three independent experiments. No sample size was calculated. The sample size of 3 was chosen to allow determination of standard deviations (SDs). The SDs obtained were small compared to the mean values which showed that the sample size of 3 was sufficiently large.<br>In vivo studies: the oral availability of each cyclic peptide was measured in groups of three rats (n=3) as the variation for the applied methods (injection, blood collection, LC-MS peptide quantification) is rather small (SDs typically < 30% of mean value). No sample size calculation was performed. The SDs obtained indicated that the sample size chosen was sufficiently large.
Data exclusions	For the IV injections into tail veins of rats (pharmacokinetic studies), data was excluded in cases where the pharmacokinatic profile indicated injection into tail tissue and thus a non-perfect IV application (as easily seen by a strongly delayed clearance profile).
Replication	All data reported in this study could be reproduced in replication experiments. Replication experiments were performed in independent experiments. The number of replicates for the different experiments were as follows:<br>Half-lives of random cyclic peptides in rat liver microsomes: Mean and SD values of three independent measurements.<br>Clearance of random cyclic peptides and two oral drugs in rat liver microsomes: Mean and SD values of three independent measurements.<br>Activities of purified cyclic peptides of Group 1: Mean and SD values of three independent measurements.<br>Thrombin inhibition of peptides 11–46: Mean and SD values of three independent measurements.<br>Proteolytic stability of peptides 11–37: Mean and SD values of three independent measurements.<br>Passive membrane permeability PAMPA of peptides 11–46: Mean and SD values of three independent measurements.<br>Metabolic stability of peptides 11–37, 43 and 46: Mean and SD values of three independent measurements.
Randomization	Rats used for the pharmacokinetic studies were randomly assigned to groups of three animals.
Blinding	Blinding was not applied in this study as all measurements were performed by instruments and the outcome could thus not be biased.

Reporting for specific materials, systems and methods

We require information from authors about some types of materials, experimental systems and methods used in many studies. Here, indicate whether each material, system or method listed is relevant to your study. If you are not sure if a list item applies to your research, read the appropriate section before selecting a response.

Materials & experimental systems

n/a	Involved in the study
☒	☐ Antibodies
☒	☐ Eukaryotic cell lines
☒	☐ Palaeontology and archaeology
☐	☒ Animals and other organisms
☒	☐ Clinical data
☒	☐ Dual use research of concern

Methods

n/a	Involved in the study
☒	☐ ChIP-seq
☒	☐ Flow cytometry
☒	☐ MRI-based neuroimaging

Animals and other research organisms

Policy information about studies involving animals; ARRIVE guidelines recommended for reporting animal research, and Sex and Gender in Research

Laboratory animals	Male Wistar Han rats (250-350 g) obtained from Charles River Laboratories (France) at an age of 9-10 weeks. Experiments were performed at an age of 10-11 weeks.
Wild animals	None
Reporting on sex	The pharmacokinetic properties are expected to be equal in rats of different sex.
Field-collected samples	None
Ethics oversight	All animal experiments in rats were carried out according to the terms of the Swiss animal protection law and were ethically reviewed and approved by the cantonal veterinary services (Canton of Vaud, Switzerland; license number: VD3606, national license number 32669).

Note that full information on the approval of the study protocol must also be provided in the manuscript.
